# Unstable Vortices, Sharp Non-Uniqueness with Forcing, and Global Smooth Solutions for the SQG Equation

**DOI:** 10.1007/s00205-026-02252-x

**Published:** 2026-10-03

**Authors:** Ángel Castro, Daniel Faraco, Francisco Mengual, Marcos Solera

**Affiliations:** 1 Instituto de Ciencias Matemáticas, CSIC-UAM-UC3M-UCM, 28049 Madrid, Spain; 2 Departamento de Matemáticas, Universidad Autónoma de Madrid, Instituto de Ciencias Matemáticas, CSIC-UAM-UC3M-UCM, 28049 Madrid, Spain; 3 Max Planck Institute for Mathematics in the Sciences, 04103 Leipzig, Germany; 4 Departament d’Anàlisi Matemàtica, Universitat de València, 46100 Burjassot, Spain

## Abstract

We prove that non-uniqueness of weak solutions to the forced α-SQG equation with Sobolev regularity $W^{s,p}$ in the supercritical regime $s < \alpha + \frac{2}{p}$, covering the 2D Euler equation (α=0), the Surface Quasi-Geostrophic equation (α=1), and the intermediate cases. A key step is the construction of smooth, compactly supported vortices that exhibit non-linear instability. As a by-product, we show that existence of global smooth solutions to the (unforced) α-SQG equation that are neither rotating nor traveling.

## Introduction and Main Results

We address the non-uniqueness of weak solutions to the generalized Surface Quasi-Geostrophic (α-SQG) equation

1.1a $$\begin{aligned} \partial _t\theta + v\cdot \nabla \theta =f, \end{aligned}$$

1.1b $$\begin{aligned} v=-\nabla ^\perp \Lambda ^{\alpha -2}\theta ,\end{aligned}$$

1.1c $$\begin{aligned} \theta |_{t=0} =\theta ^\circ , \end{aligned}$$

posed on $\mathbb {R}^2$, for some given external force *f* and initial datum $\theta ^\circ$, where $\Lambda =(-\Delta )^{\nicefrac {1}{2}}$. The velocity *v*(*t*, *x*) is recovered from the scalar θ(t,x) through (1.1b), which we find convenient to refer to here as the α-*Biot-Savart law*

1.2 $$\begin{aligned} v(t,x) =C_\alpha \int _{\mathbb {R}^2}\frac{(x-y)^\perp }{|x-y|^{2+\alpha }}\theta (t,y)\,\textrm{d}y, \end{aligned}$$

where $$C_\alpha =\frac{2^{\alpha }}{2\pi }\frac{\Gamma (1+\frac{\alpha }{2})}{\Gamma (1-\frac{\alpha }{2})}>0$$.

The α-SQG model was proposed by Córdoba, Fontelos, Mancho and Rodrigo [42] as an interpolation between the 2D Euler equation (α=0) and the SQG equation (α=1). The Euler equation describes the motion of an ideal, incompressible fluid, capturing vorticity evolution (see e.g. [107]). The SQG equation extends this concept to geophysical fluids, modeling temperature evolution in quasi-geostrophic atmospheric and oceanic flows (see, e.g. [116]). This family of active scalar equations has attracted significant interest since the seminal work of Constantin, Majda, and Tabak [40], which highlighted analogies between SQG and the 3D Euler equation. We will generally refer to θ as the temperature, unless specifically discussing 2D Euler, where $\theta =\nabla ^\perp \cdot v$ is the vorticity.

The potential lack of uniqueness is a central issue in fluid mechanics, relevant to both the question of determinism and its relationship with chaos and turbulence (see e.g. [89, 103, 106, 135]). From a mathematical point of view, given a parametrized family of function spaces $X^s$, where *s* typically quantifies the regularity of the solution θ, a natural question is to determine that

1.3 $$\begin{aligned} s_U\equiv \text { threshold for Uniqueness}. \end{aligned}$$

Notice that (1.3) depends on α and the baseline space. In terms of Sobolev regularity $\theta \in W^{s,p}$, we have the following natural conjecture:

1.4 $$\begin{aligned} s_U(\alpha ,p)=\alpha +\frac{2}{p}. \end{aligned}$$

The well-posedness of the α-SQG equation has been proved in the subcritical regime $s>\alpha +\frac{2}{p}$: globally for the 2D Euler equation by Bourguignon and Brezis [12], locally for the SQG equation by Chae [32], and locally, at least for p=2, in the intermediate cases by Chae and Wu [35]. Heuristically, this is because θ is transported by a velocity $v\in W^{s+1-\alpha ,p}\subset C^{1+}$. These results, based on standard energy methods, readily extend to the case with forcing in the natural space

1.5 $$\begin{aligned} f\in L^1([0,T],W^{s,p}\cap \dot{H}^{\frac{\alpha -2}{2}}), \end{aligned}$$

where $\dot{H}^{\frac{\alpha -2}{2}}$ is the energy space associated with the Hamiltonian of the α-SQG equation.

In the celebrated work [135], Yudovich proved that the 2D Euler equation (α=0) is also globally well-posed at the critical point (s,p)=(0,∞). In fact, Yudovich’s energy method can be adapted to prove uniqueness, assuming existence, along the critical line for α=0, and other critical spaces such as $H^{\alpha +1}$ for $0 \le \alpha \le 1$. We caution that well-posedness implies uniqueness, but the converse is not true. Counterexamples by Elgindi and Jeong [64], Córdoba and Martínez-Zoroa [45], and Jeong and Kim [98] show critical exponents where ill-posedness occurs, with solutions losing regularity instantaneously in spaces where uniqueness holds. See also [44, 47, 48, 97] for ill-posedness in supercritical spaces, as well as [6, 17, 37] for the related issue of propagation of regularity.

Overall, the main obstruction for proving Conjecture (1.4) is to establish non-uniqueness in the supercritical regime $s<\alpha +\frac{2}{p}$. In this work, we show that this holds in the case with forcing (1.5).

Conjecture (1.4) remains an outstanding problem in the case f=0. Remarkably, the construction of solutions that violate both uniqueness and Hamiltonian conservation has been successfully addressed in recent years through the method of convex integration, introduced into fluid dynamics by De Lellis and Székelyhidi [53]. See Sects. 1.5 and 1.6 for discussions on convex integration and conservation laws, as well as the reviews [22, 54–56]. Regarding the 2D Euler equation, θ is the vorticity, and the Hamiltonian equals the kinetic energy at the velocity level. In terms of Hölder regularity, Giri and Radu [80] recently solved the 2D Onsager conjecture by constructing non-trivial Euler flows $v\sim \Lambda ^{-1}\theta \in C^{\nicefrac {1}{3}-}$. More recently, Bruè, Colombo, and Kumar [16] constructed the first non-trivial Euler flows with integrable vorticity $\theta \in L^{p}$ for some small p>1 below the Onsager threshold. Regarding the SQG equation, the construction of non-trivial solutions was first established by Buckmaster, Shkoller, and Vicol [21] and was recently improved to $\Lambda ^{-1} \theta \in C^{1-}$ by Dai, Giri, and Radu [51], and by Isett and Looi [94], thereby solving the Onsager conjecture for SQG. More recently, Zhao [136] extended this result to $\Lambda ^{-1} \theta \in C^{\frac{1+2\alpha }{3}-}$ for $0\le \alpha \le 1$. Notice that all the above convex integration constructions lie within the regime where the Hamiltonian does not need to be conserved. Since this is strictly contained within the broader regime where non-uniqueness is expected, it leaves a significant gap in the theory of non-uniqueness.

In the case with forcing, similar non-uniqueness results have been established using convex integration by Bulut, Huynh, and Palasek [25], and by Dai and Peng [52]. Indeed, for the 3D Euler equ. [24] it is possible to surpass the Onsager threshold up to $v\in C^{\nicefrac {1}{2}-}$. However, to the best of our knowledge, non-unique solutions with some Sobolev or Hölder regularity at the level of θ had not been constructed until now for the α-SQG equation, even in the case with forcing.

This work is devoted to exploring another mechanism for non-uniqueness, based on the self-similar instability approach introduced by Jia and Šverák in the context of the 3D Navier-Stokes equ. [99]. In a remarkable pair of papers [129, 130], Vishik successfully established non-uniqueness for the forced 2D Euler equation (α=0) below the Yudovich well-posedness class (s,p)=(0,∞). The present paper extends Vishik’s non-uniqueness theorem to the forced α-SQG equations for the full supercritical Sobolev regime and $0 \le \alpha \le 1$.

### Theorem 1.1

(Sobolev non-uniqueness) Let $0\le \alpha \le 1$, s≥0 and $1\le p\le \infty$ satisfy

1.6 $$\begin{aligned} s<\alpha +\frac{2}{p}. \end{aligned}$$

There exist T>0 and a force (1.5) such that there are uncountably many solutions

1.7 $$\begin{aligned} \theta _\epsilon \in L^\infty ([0,T],W^{s,p}\cap \dot{H}^{\frac{\alpha -2}{2}}) \end{aligned}$$

to the α-SQG equation (1.1) starting from $\theta ^\circ =0$.

As we will explain in more detail in Sect. 3, Vishik’s result (Theorem 3.2) corresponds to the specific case α=s=0. Indeed, it is a particular case of a refined version of Theorem 1.1, namely, our Theorem 3.1.

### Improvements and Corollaries

The regularity of the force and the solutions can be described in various other function spaces. Thus, Theorem 1.1 allows for certain improvements and by-products, which we summarize below and will later state as theorems and corollaries in the introduction.

Firstly, let us remark that both the force *f* and the main solution $\theta _0$ from Theorem 1.1 belong to $C_c^\infty$ for positive times. Moreover, for every $k\in \mathbb {N}$, the other solutions $\theta _\epsilon$ can be upgraded to be in $C_c^{k}$ for positive times. These estimates inevitably deteriorate as t→0. Although spatial regularity cannot exceed the critical line, temporal regularity can easily be improved to Lipschitz, or even enhanced with additional derivatives, by choosing the parameter *a* appropriately in Sect. 2.1.

As a consequence of the smoothness of our solutions, Theorem 1.1 can be generalized to any space that is scaling-supercritical with respect to the α-SQG equation and, at the same time, includes smooth, compactly supported functions, such as Besov or Triebel–Lizorkin spaces. In this work, we state the theorems in the context of classical Sobolev spaces for the sake of simplicity.

In the scale of Lebesgue spaces (s=0), Theorem 1.1 shows non-uniqueness within the DiPerna-Majda existence class for the 2D Euler equation [60], which corresponds to Vishik’s non-uniqueness theorem, as well as within the Resnick-Marchand existence class for the SQG equation [108, 118], and the Chae-Constantin-Córdoba-Gancedo existence class for the generalized SQG equation [33], all in the presence of forcing.

In the scale of Hölder spaces, which we express as $\Lambda ^{-1}\theta \in C^\gamma$ to facilitate comparison with the Onsager exponent, we have the following natural conjecture

1.8 $$\begin{aligned} \gamma _U(\alpha )=1+\alpha . \end{aligned}$$

Similarly to the Sobolev case, in the subcritical regime $\gamma > 1 + \alpha$, we would have $v \sim \Lambda ^{\alpha - 1} \theta \in C^{1+}$. Well-posedness in subcritical Hölder spaces has been established for the 2D Euler equation by Wolibner [133] and Hölder [88], and for the SQG equation by Wu [134] and by Ambrose, Cozzi, Erickson, and Kelliher [3]. Uniqueness can be deduced, assuming existence, in some critical Hölder spaces as a consequence of the weak-strong uniqueness principle (see e.g. [132]). In contrast, there are known counterexamples of such critical Hölder spaces that exhibit strong ill-posedness. This was first established by Bourgain and Li for the Euler equation [11], and also by Elgindi and Masmoudi [65]. These results were later extended to both the SQG and generalized SQG equations by Córdoba and Martínez-Zoroa [45, 46].

By the extension of Morrey’s inequality to fractional Sobolev spaces [58], we deduce, as a corollary of Theorem 1.1, non-uniqueness for the full supercritical Hölder regime in the case with forcing.

#### Corollary 1.1

(Hölder non-uniqueness) In the setting of Theorem 1.1, for every fixed

$$ \gamma <1+\alpha , $$

the solutions and the force can be chosen to satisfy

$$ \Lambda ^{-1}\theta _\epsilon \in L^\infty ([0,T],C^\gamma ), \quad \quad \Lambda ^{-1}f\in L^1([0,T],C^\gamma ). $$

Finally, we emphasize that our solutions satisfy both the Hamiltonian identity (Corollary 1.4) and the renormalization property (Corollary 1.5) in the presence of forcing. See Sects. 1.5 and 1.6 for the precise definitions of the Hamiltonian (1.11) and the renormalization (1.19), respectively.

Up to this point, we have discussed the improvements and corollaries regarding non-uniqueness with forcing. A key intermediate step of independent mathematical and physical interest involves the explicit construction of smooth, compactly supported vortices that exhibit non-linear instability (Theorem 1.2 and Corollary 1.2). As a corollary, we show the existence of global smooth solutions to the (unforced) α-SQG equation that are *n*-fold symmetric, non-stationary, and converge to a radial temperature as $t\rightarrow \infty$ (Corollary 1.3). See Sect. 2 for the precise definition of vortices and *n*-fold symmetry.

### Main Novelties

The starting point of our work is the groundbreaking result of Vishik on the non-uniqueness of solutions to the forced 2D Euler equ. See [129, 130] for the original papers and the book [2] by Albritton, Bruè, Colombo, De Lellis, Giri, Janisch, and Kwon for a careful and detailed exposition. Vishik’s work raised the question of the validity of analogous results for the more singular generalized SQG equations, and more importantly, for the SQG equation itself. However, it appears quite challenging to implement Vishik’s argument for these non-linear and singular counterparts of the 2D Euler equation. Motivated by this, we devised an alternative approach for the 2D Euler equ. in [31], which not only substantially simplifies Vishik’s proof but also appears robust enough to be adapted to other situations. This is what we have accomplished in the current paper. However, essentially every step of the construction requires new ideas, with the SQG case being particularly delicate due to the non-compact relationship between the velocity and the advected scalar. We will next explain the parallels and innovations we consider noteworthy.

The proof of Theorem 1.1 follows the general strategy presented in [31], which is that of [2, 129, 130] except for the construction of the unstable vortex. This approach consists of four steps:

1. Construct a piecewise constant unstable vortex.
2. Regularize the unstable vortex mantaining instability.
3. Prove that the regularized vortex is self-similarly unstable.
4. Prove that the regularized vortex is non-linearly unstable.

Let us summarize the main differences with respect to our proof in the 2D Euler case:

1. While for α=0 Step (1) reduces to an elementary computation, for α>0 the non-locality introduced by the α-Biot-Savart law necessitates dealing with a parametric integral that cannot be computed. Despite this lack of explicitness, we are able to show that there are piecewise constant unstable vortices by obtaining precise asymptotics in Sect. 4.1.
2. Step (2) requires correctly expanding the linear stability equation into a leading term and a lower-order term that behaves like $\frac{\varepsilon ^{1-\alpha }}{1-\alpha }$. In the limiting case α=1, the roles of these operators are reversed, completely changing the nature of the problem. In Sect. 5.4, we show that even in this situation it is possible to handle the operators and apply a fixed point argument to find a regularized unstable vortex.
3. Step (3) involves decomposing the linearization into an operator that generates a contraction semigroup and a compact operator *K*. However, this operator *K* loses compactness for SQG. We bypass this obstacle in Sect. 6.6 by decomposing *K* into a skew-adjoint operator, which preserves the growth bound of the semigroup, along with a compact commutator.
4. Step (4) is carried out in [2, 31, 129, 130] through energy estimates on first-order derivatives in polar coordinates, applied in the correct order. Here, achieving the full supercritical regime $s < \alpha + \frac{2}{p}$ requires working with higher-order derivatives. The resulting energy space consists of functions that vanish at the origin. Moreover, to manage the many terms, we introduce an inductive argument in Sect. 7.5, which significantly streamlines the proof. In addition, a delicate estimate involving the commutator is necessary in the SQG case.

During the finalization of this work, a clever trick discovered by Golovkin in the 1960s [82] was highlighted by Dolce and Mescolini [61], showing that by absorbing the quadratic interaction of the linear instability into the forcing term, it is possible to bypass the non-linear instability step and construct two distinct solutions (instead of uncountably many). In Sect. 3.3, we revisit this trick in the context of the α-SQG equation.

Nonetheless, our non-linear instability approach has several remarkable by-products. First, it rigorously proves the existence of non-linearly unstable vortices for the unforced α-SQG equation. Second, it provides a robust framework for transitioning from linear to non-linear instability in arbitrarily high Sobolev regularity. Third, by reversing the arrow of time, it shows the existence of global smooth solutions that are neither rotating nor traveling. See Sects. 1.3 and 1.4 for more details. Additionally, it could be useful for showing the potential lack of uniqueness in the absence of forcing, as it offers a more precise representation of the α-SQG dynamics.

### Unstable Vortices

The stability analysis of simple steady states in the 2D Euler equ. is a classical problem that dates back to the works of Kelvin [126], Rayleigh [117], Orr [114], Taylor [125], and many others. Although some explicit shear flows were known to be unstable (see e.g. [62]), to the best of our knowledge, the first rigorous construction of an unstable vortex was achieved by Vishik (see also [2]). Vishik’s approach is based on bifurcation from neutral modes, a method that traces back to Tollmien’s work on shear flows [127]. This technique was later rigorously applied by Lin to shear flows in a bounded channel and in $\mathbb {R}^2$, and to rotating flows in an annulus [104].

Although there was numerical evidence supporting the existence of unstable vortices for the α-SQG equation (see e.g. [87]), to the best of our knowledge, they had not yet been rigorously constructed for $0<\alpha \le 1$. We refer to the work of Friedlander and Shvydkoy [76] for the analysis of the spectrum of SQG and the existence of unstable eigenvalues for an oscillating shear flow.

In this work, we adapt our construction of smooth, compactly supported, unstable vortices for the 2D Euler equ. [31] to the full range $0\le \alpha \le 1$. In particular, this rigorously proves the existence of non-linearly unstable vortices for the α-SQG equation with f=0. Moreover, we prove a stronger property, namely that the vortex exhibits non-uniqueness at t=-∞ in the sense of the following statement:

#### Theorem 1.2

(Non-uniqueness at t=-∞) Let $0\le \alpha \le 1$ and n≥2. There exists a vortex

$$ \bar{\Theta }\in C_c^\infty \cap \dot{H}^{\frac{\alpha -2}{2}}, $$

such that, for every m>α+1, there is an *n*-fold symmetric solution to the (unforced) α-SQG equation

$$ \theta \in C((-\infty ,0],H^m\cap \dot{H}^{\frac{\alpha -2}{2}}), $$

satisfying

$$ \Vert \theta (t)-\bar{\Theta }\Vert _{H^m}\sim e^{\Re \lambda t}, $$

as $t\rightarrow -\infty$, for some $\lambda \in \mathbb {C}$ with $\Re \lambda >0$. In particular, $\Vert \theta (t)\Vert _{\dot{H}^{\frac{\alpha -2}{2}}} =\Vert \bar{\Theta }\Vert _{\dot{H}^{\frac{\alpha -2}{2}}}$ for all t≤0.

Above, the complex number λ corresponds to the eigenvalue of the linear instability associated with $\bar{\Theta }$. By starting at a finite time $t_0<0$ sufficiently far back in time, the non-uniqueness at t=-∞ (Theorem 1.2) implies the non-linear instability as it is usually stated (Corollary 1.2). Notice that, by shifting the time interval, we can take that $0=t_0<T$.

#### Corollary 1.2

(Non-linearly unstable vortex) Let $0\le \alpha \le 1$ and n≥2. There exists δ>0 such that, for every ε>0 and m>α+1, there exist T>0 and an *n*-fold symmetric solution to the (unforced) α-SQG equation

$$ \theta \in C([0,T],H^m\cap \dot{H}^{\frac{\alpha -2}{2}}), $$

satisfying

1.9 $$\begin{aligned} \Vert \theta (0)-\bar{\Theta }\Vert _{H^m} \le \varepsilon , \end{aligned}$$

and

1.10 $$\begin{aligned} \Vert \theta (T)-\bar{\Theta }\Vert _{H^m}\ge \delta , \end{aligned}$$

where $\bar{\Theta }$ is the vortex from Theorem 1.2.

Corollary 1.2 shows energy pump of certain solutions to the α-SQG equation, aligning with the work of Kiselev and Nazarov [101] for SQG. Double exponential growth of the gradient of the vorticity was established by Kiselev and Šverák for the 2D Euler equation [102] in the unit disc. Exponential growth of the gradient of the vorticity was provided for 2D Euler in the torus by Zlatoš [137]. For SQG, He and Kiselev [86] showed exponential growth of the $C^{1,\gamma }$-norm of the temperature in $\mathbb {R}^2$.

As we introduced in Sect. 1.2, the construction of $\bar{\Theta }$ is divided into several steps. The first step involves constructing a piecewise constant unstable vortex. While this idea was inspired by previous constructions for shear flows in the 2D Euler equation (see e.g. [62, Chapter 4]), we remark that our construction operates at the level of the temperature, in contrast to the classical Rayleigh stability equation, which deals with the stream function. Secondly, we find a suitable regularized unstable vortex through a fixed-point argument. The idea that regularized unstable shear flows retain their instability was proposed by Grenier for the Euler equation [84]. In this work, we rigorously prove that this is the case for vortices in the α-SQG equation. Finally, the last step consists of proving that the non-linear effects are negligible within a certain time interval.

In [84] Grenier developed a program to transition from linear to non-linear instability. In particular, Grenier showed that, given a linearly unstable smooth shear flow for the Euler equation, it is possible to deduce the properties (1.9) and (1.10). The transition from linear to non-linear instability has been also analyzed by Bulut and Dong [23] for the SQG equation with dissipation. Grenier’s strategy for the Euler equation appears robust enough to be applied to other equations, such as the α-SQG equation. Thus, by combining this approach with our Theorem 3.3, it is plausible that this could provide an alternative proof of Corollary 1.2. In order to upgrade the non-linear instability to non-uniqueness at t=-∞ (Theorem 1.2), we apply a delicate bootstrapping argument to obtain additional exponential decay uniformly in time.

Recently, Dolce and Mescolini [61] presented another flexible strategy for constructing linearly unstable vortices for the 2D Euler equation. An interesting question is whether a similar variety of unstable vortices also exists for α>0. It is worth noting that the non-uniqueness of Leray-Hopf solutions to the forced 3D Navier-Stokes equation was established by Albritton, Bruè, and Colombo [1] by carefully adapting Vishik’s unstable vortex in the axisymmetric setting. The unstable vortices constructed in [31, 61], while different from Vishik’s, satisfy the conditions of [1], offering an alternative example of non-unique Leray-Hopf solutions with forcing.

### Global Smooth Solutions

A significant distinction between the 2D Euler equation (α=0) and the SQG equation (α=1) is the potential lack of global well-posedness in the SQG case, as well as in the intermediate cases (0<α<1). Whether the SQG equation develops finite-time singularities remains an outstanding open problem in mathematical fluid mechanics (see e.g. [34, 40, 50]).

In this regard, the construction of global smooth solutions that exhibit non-trivial dynamics has attracted considerable attention from the mathematical community in recent years. In [27], the first author, in collaboration with Córdoba and Gómez-Serrano, constructed the first non-trivial family of global smooth solutions to the SQG equation, consisting of rotating 3-fold symmetric solutions with $C^4$ regularity bifurcating from a smooth annular profile. The argument required a computer-assisted proof, which could potentially be adapted to produce more regular *n*-fold symmetric solutions. Smooth rotating solutions bifurcating from vortex points can be found in [4]. The construction of smooth traveling solutions has been carried out by Gravejat and Smets [83], by Godard-Cadillac [81], as well as by Ao, Dávila, del Pino, Musso, and Wei [4].

In [43], Córdoba, Gómez-Serrano, and Ionescu constructed global solutions to the α-SQG patch equation consisting of non-stationary solutions which converge to the stationary half-plane patch as $t \rightarrow \infty$. In this line of thought, another approach to constructing families of global smooth solutions around steady states could involve the mechanism of inviscid damping. This has been successfully applied to the 2D Euler equation for perturbations of the Couette flow $\bar{\Theta }=-1$ by Bedrossian and Masmoudi [8], and by Ionescu and Jia [90], and later extended to general monotonic shear flows by Ionescu and Jia [91] and by Masmoudi and Zhao [109]. Unfortunately, recent work by Gómez-Serrano, Ionescu and Jia (see [92, Section 3]) suggests that the approach of inviscid damping, with the aim of producing globally smooth solutions, fails for the Couette flow for any α>0, due to a forward cascade that leads to a loss of regularity in finite time. Therefore, for α>0, it remains unclear whether a smooth steady state $\bar{\Theta }$ to the α-SQG equation can exist with a (small) open neighborhood of perturbations that converge to $\bar{\Theta }$ as $t\rightarrow \infty$, or even whether these perturbations produce global smooth solutions.

In this work, we show that by reversing the arrow of time in Theorem 1.2, there exist particular perturbations of the vortex $\bar{\Theta }$ that converge to it as $t \rightarrow \infty$. As a result, we establish the existence of global smooth solutions to the α-SQG equation that are neither rotating nor traveling.

#### Corollary 1.3

(Global smooth solutions) Let $0\le \alpha \le 1$, n≥2 and m>α+1. There exists a global solution to the (unforced) α-SQG equation

$$ \theta \in C([0,\infty ),H^m\cap \dot{H}^{\frac{\alpha -2}{2}}), $$

which is *n*-fold symmetric, non-stationary, and converges to the vortex $\bar{\Theta }\in C_c^\infty \cap \dot{H}^{\frac{\alpha -2}{2}}$ from Theorem 1.2 in $H^m$ as $t\rightarrow \infty$.

Notice that our solutions are defined for positive times, thus strictly speaking they are global *forward* in time. We remark that our approach allows for the construction of global solutions in $H^m$ for any fixed m>α+1. However, it is easy to see within the construction that a Beale-Kato-Majda argument implies that the solutions remain $C^\infty$ for all t≥0.

In these results we have used *m* (instead of *s*) to distinguish it from the supercritical case (s<α+1) and to facilitate comparison with Sect. 7, where everything is written in terms of the higher-order norms $H^m$. Although our non-linear analysis allows us to control any Sobolev norm, we chose to state the results in the Hilbert case for simplicity and because it is more commonly used in the α-SQG setting.

Recently, the existence of self-similar spirals was established by García and Gómez-Serrano [78] for the α-SQG equation, with 0<α<1, and by Shao, Wei, and Zhang for the 2D Euler equation [120], building on the previous work of Elling [67]. In these examples it holds that $\theta \in L^p$, leading to a promising scenario for non-uniqueness without forcing. Other promising scenarios based on symmetry breaking and self-similarity for the 2D Euler equation have been proposed recently by Bressan, Shen, and Murray [13, 14], as well as by Elgindi, Murray, and Said [66].

### Hamiltonian Identity

The α-SQG equation formally possesses a large class of conservation laws. The Hamiltonian $\mathcal {H}=\mathcal {H}_\alpha$,

1.11 $$\begin{aligned} \mathcal {H}(t) =\frac{1}{2}\Vert \theta (t)\Vert _{\dot{H}^{\frac{\alpha -2}{2}}}^2 =\frac{1}{2}\Vert v(t)\Vert _{\dot{H}^{-\frac{\alpha }{2}}}^2, \end{aligned}$$

is particularly meaningful because the α-SQG equation can be interpreted as geodesics with respect to the metric (1.11) on the Lie group of volume-preserving diffeomorphisms (see e.g. [5, 7, 21, 111, 131]). By a short energy estimate, it is straightforward to check that smooth solutions to the α-SQG equation satisfy the *Hamiltonian identity*

1.12 $$\begin{aligned} \frac{\textrm{d}}{\textrm{d}t}\mathcal {H} =\langle f,\theta \rangle _{\dot{H}^{\frac{\alpha -2}{2}}}. \end{aligned}$$

In particular, $\mathcal {H}$ is conserved for classical solutions in the case f=0. Similarly to the question of uniqueness, the lack of Hamiltonian conservation is a cornerstone in the theory of turbulence (see e.g. [77]). Analogously to (1.3), a natural question is to determine

1.13 $$\begin{aligned} s_H\equiv \text { threshold for Hamiltonian identity}, \end{aligned}$$

which again depends on α and the baseline space $X^s$.

In terms of Hölder regularity, which is usually expressed as $\Lambda ^{-1}\theta \in C^\gamma$ in the α-SQG setting, Onsager conjectured in the celebrated work [113] that $\gamma _H = \nicefrac {1}{3}$ for the Euler equation, which is dimensionless at the velocity level. The conservative part of Onsager’s conjecture was proved by Constantin, E, and Titi [38], building on a partial result by Eyink [69]. Onsager’s conjecture can be extended to the α-SQG equation as follows

1.14 $$\begin{aligned} \gamma _H(\alpha )=\frac{1+2\alpha }{3}. \end{aligned}$$

As we mentioned earlier in the introduction, the non-conservative part of the Onsager-type conjecture (1.14) has been recently proven using convex integration for the 2D Euler equation [80], the SQG equation [51, 94], and the intermediate cases [136]. The Onsager conjecture for the 3D Euler equation was previously solved by Isett [93], by Buckmaster, De Lellis, Székelyhidi, and Vicol [19] for dissipative solutions, and by Giri, Kwon, and Novack [79] for locally dissipative solutions in Besov spaces. Additionally, see the work of Masmoudi, Novack, and Vicol [20, 112] on the Hilbert case. We also refer to the works for Scheffer [119] and Shnirelman [121] for the first constructions of non-unique Euler flows. Notice that all these results prove non-uniqueness up to $\gamma _H=\frac{1+2\alpha }{3}$, leaving a gap in the conjectured $\gamma _U=1+\alpha$ in the case without forcing.

In the context of Lebesgue spaces $\theta \in L^p$, Duchon and Robert were the first to prove that 2D Euler vorticities with $p > \nicefrac {3}{2}$ conserve the Hamiltonian [63]. This was extended to the borderline case $p = \nicefrac {3}{2}$ by Cheskidov, Lopes Filho, Nussenzveig Lopes, and Shvydkoy [36]. These results lead to the natural conjecture for $p_H(\alpha )$ in the case of the 2D Euler equation (α=0)

1.15 $$\begin{aligned} p_H(0)=\nicefrac {3}{2}. \end{aligned}$$

The first non-conservative results in weak Lebesgue spaces were obtained by Bruè and Colombo in Lorentz spaces [15], and by Modena and Buck in Hardy spaces [18]. Remarkably, Bruè, Colombo, and Kumar [16] recently constructed non-trivial 2D Euler vorticities $\theta \in L^{1 + \varepsilon }$. As already pointed out by the authors, this yields the first example of 2D Euler flows with uniformly integrable vorticity that are not vanishing viscosity solutions. A natural question is whether this new convex integration scheme can be extended to reach (1.15).

As we discussed for the Hölder case, even if the Onsager exponent (1.15) can be achieved with these techniques, there would still be a gap to the conjectured Yudovich exponent $p_U(0) = \infty$ in the case without forcing. In this regard, the third author constructed initial data $\theta ^\circ \in L^p$, for any fixed p<∞, admitting infinitely many solutions with non-increasing Hamiltonian [110], thus showcasing the sharpness of the weak-strong uniqueness principle and Yudovich’s proof of uniqueness. Unfortunately, the vorticity information is lost for positive times in [110], as the weak solutions are only understood at the velocity level.

Concerning the SQG equation (α=1) Isett and Vicol [96] proved Hamiltonian conservation provided that $\theta \in L^3$. On the one hand, this implies that $p_H(1)\le 3$. On the other hand, since $C^0\subset L^3$ in $\mathbb {T}^2$, this solves the conservative part of the Onsager-type conjecture (1.14) for α=1. See also the recent work of Isett and Ma [95] on conservation laws for α-SQG.

By the Sobolev embedding theorem, a natural extension of Conjecture (1.15) for 2D Euler vorticities $\theta \in W^{s,p}$ would be $s_H(0, p) = \frac{2}{p} - \frac{4}{3}$. A similar exponent $s_H(\alpha ,p)$ could be derived for the general case $0 \le \alpha \le 1$. The problem of constructing non-unique solutions with some Sobolev regularity remains widely open for the α-SQG equation without forcing.

The Hamiltonian structure of α-SQG makes it a rare and significant example of active scalars. Notably, the method of convex integration has produced much more regular solutions when applied to other active scalars that lack a Hamiltonian structure. A key prototype is the IPM equation, known for its gradient flow structure [115] and its role as a model to address fluid instabilities through convex integration. In this regard see e.g. [26, 28–30, 41, 75, 124]. In fact, very general classes of active scalars, explicitly excluding SQG, can be treated using variants of convex integration, yielding bounded [122] and even continuous [96, 136] turbulent solutions.

Finally, we remark that vanishing viscosity solutions to the 2D Navier-Stokes equations with uniform integrability are known to conserve the Hamiltonian. This was proved in [36] for p>1, and recently extended to the borderline case p=1 by De Rosa and Park [57]. A similar result was obtained by Constantin and Ignatova in $$ L^2$$ for the dissipative SQG equation in $$\mathbb {T}^2$$ [39], and previously in $$\mathbb {R}^2$$ by Berselli [10]. This discrepancy in Hamiltonian conservation between ideal solutions and ideal limits has also been observed in other fluid models, such as 3D Magnetohydrodynamics (MHD) by the second author and Lindberg [71].

Therefore, the search for physically realizable solutions with uniformly integrable θ for the 2D Euler and SQG equations involves imposing that the Hamiltonian is conserved, or more generally the Hamiltonian identity in the presence of forcing. As a consequence of the regularity of the solutions in Theorem 3.1, we deduce that our solutions satisfy this property. Thus, satisfying the Hamiltonian identity is not a criterion for uniqueness, at least in the case with forcing.

#### Corollary 1.4

In the setting of Theorem 1.1, the solutions $\theta _\epsilon$ and the force *f* satisfy the Hamiltonian identity (1.12).

### Renormalization Property

The Hamiltonian is not the only conserved quantity in α-SQG. Since this is a transport equation, any functional of the temperature is formally preserved by the flow. Given a classical solution θ and a function β, the *temperature Casimir*

1.16 $$\begin{aligned} C_\beta (t):=\int _{\mathbb {R}^2}\beta (\theta (t,x))\,\textrm{d}x, \end{aligned}$$

satisfies the identity

1.17 $$\begin{aligned} \frac{\,\textrm{d}}{\,\textrm{d}t} C_\beta =\int _{\mathbb {R}^2}\beta '(\theta )f\,\textrm{d}x. \end{aligned}$$

In particular, $C_\beta$ is conserved in the case f=0. The Casimir temperature associated with the function $\beta =\frac{1}{q}|\cdot |^q$ for $1\le q<\infty$ corresponds to the *q*-energy (or momentum)

$$ \mathcal {E}_q(t) =\frac{1}{q}\Vert \theta (t)\Vert _{L^q}^q. $$

In this regard, a solution to the α-SQG equation θ satisfies the *q*-*energy balance* if

1.18 $$\begin{aligned} \frac{\,\textrm{d}}{\,\textrm{d}t} \mathcal {E}_q =\int _{\mathbb {R}^2}\theta |\theta |^{q-2}f\,\textrm{d}x. \end{aligned}$$

For the 2D Euler equation, $\mathcal {E}=\mathcal {E}_2$ represents the enstrophy, which plays a crucial role in 2D turbulence (see e.g. [37, 70, 105]). Indeed, in turbulent regimes, it is plausible for the Hamiltonian $\mathcal {H}$ to be conserved, energy $\mathcal {E}_q$ to dissipate, and non-uniqueness to arise. The existence of two quadratic invariants, both preserved by smooth solutions−one more robust than the other−forms the precise framework of the celebrated self-organization conjecture [85]. Vorticity evolution in 2D Euler is a prime example of self-organization, and the α-SQG equation aligns with this theory, at least at a formal level.

Recent studies have implemented convex integration schemes compatible with conservation of magnetic helicity in MHD [9, 72–74], which represents another canonical example of self-organization. Following this perspective, it is plausible that convex integration could be effective in regimes where the Hamiltonian is conserved for the α-SQG equation but dissipation of *q*-energies is feasible. Thus, whether convex integration can be applied in the Hamiltonian conservation regime, or whether it can be shown to be impossible, remains a compelling and challenging open problem.

The Casimirs balance can be described locally through the concept of renormalized solutions. A solution to the α-SQG equation θ satisfies the *renormalization property* if

1.19 $$\begin{aligned} \partial _t\beta (\theta ) +v\cdot \nabla (\beta (\theta )) =\beta '(\theta )f \end{aligned}$$

holds in the sense of distributions for every $\beta \in C_c^1(\mathbb {R})$; that is,

$$ \int _0^T\int _{\mathbb {R}^2}(\beta (\theta )(\partial _t\varphi +v\cdot \nabla \varphi )+\beta '(\theta )f\varphi )\,\textrm{d}x\,\textrm{d}t =-\int _{\mathbb {R}^2}\beta (\theta ^\circ (x))\varphi (0,x)\,\textrm{d}x $$

for all test function $\varphi \in C_c^1([0,T)\times \mathbb {R}^2)$. Notice that the concept of weak (or distributional) solution corresponds to $\beta \rightarrow \textrm{id}$. By integrating (1.19) and applying that *v* is weakly divergence-free, we recover the Casimir identity (1.17). Formally, ignoring the singularity at zero and its growth at infinity, we can also recover the *q*-energy balance (1.18).

Analogously to (1.13), a natural question is to determine the threshold for the renormalization property, as well as the *q*-energy balance. For the 2D Euler equation, Crippa and Spirito shown that vanishing viscosity limits are renormalized [49] provided that $\theta \in L^p$ with p>1. In fact, by the DiPerna-Lions theory [59] any weak solution with vorticity in $L^2$ is renormalized. However, it might be that large moments are dissipated in the vanishing viscosity limit [37].

As a consequence of the regularity of the solutions of Theorem 3.1, they can be shown to be renormalized, and thus being renormalized is not a criteria for uniqueness, at least in the case with forcing. Moreover, they satisfy the *q*-energy balance, provided that $\mathcal {E}_q$ is well-defined.

#### Corollary 1.5

In the setting of Theorem 1.1, the solutions $\theta _\epsilon$ and the force *f* satisfy the renormalization property, and also the *q*-energy balance for *q* moments as long as they are well defined.

As we already mentioned, both Corollaries 1.4 and 1.5 follow from the regularity obtained in Theorem 3.1, by taking ε>0 small enough if necessary, along with the conservative results discussed in Sects. 1.5 and 1.6. Alternatively, they can be deduced more directly as follows. On the one hand, the force and the solutions are classical for positive times, so they trivially satisfy all the conservation laws for t>0. On the other hand, the solutions $\theta _\epsilon$ converge to zero as t→0 in the corresponding Sobolev spaces (Proposition 2.2), which is sufficient to verify that the conservation laws are also satisfied at t=0.

Finally, we remark that most of the literature mentioned in this section refers to the periodic box $\mathbb {T}^2$, while our theorems are stated in $\mathbb {R}^2$. Although our solutions can be shown to have compact support, the corresponding velocity does not necessarily. A natural problem is to extend our construction to the periodic box, as well as to bounded domains.

### Organization of the Paper

We start Sect. 2 by deriving the stability equation in self-similar coordinates. Next, we outline the proof of the main theorems in Sect. 3. In Sect. 4 we construct the piecewise constant unstable vortex, and we find a regularization in Sect. 5. Finally, in Sects. 6 and 7 we prove that the vortex is also self-similarly and non-linearly unstable, respectively.

## Stability Equation in Self-Similar Coordinates

In this section, we provide some preliminary definitions and results that will be used throughout the paper. Firstly, we rewrite the α-SQG equation in self-similar coordinates. Secondly, we derive both the linear and non-linear stability equations around vortices.

### Self-Similar Coordinates

In this section we write the α-SQG equation (1.1) in *self-similar coordinates*

2.1 $$\begin{aligned} \tau =\frac{1}{ab}\log t, \quad \quad X=\frac{x}{(abt)^{\nicefrac {1}{a}}}, \end{aligned}$$

in terms of two parameters 0<a,b≤1, to be determined. Notice that the logarithmic time interval is $\mathbb {R}$ (instead of [0,∞)). In particular, the physical initial time t=0 corresponds to the logarithmic time $\tau =-\infty$. In the next proposition we derive the *self-similar* α-*SQG equation*.

#### Proposition 2.1

The pair (θ,f) given by the change of variables

2.2a $$\begin{aligned} \theta (t,x)&=(abt)^{\frac{\alpha }{a}-1}\Theta (\tau ,X),\end{aligned}$$

2.2b $$\begin{aligned} f(t,x)&=(abt)^{\frac{\alpha }{a}-2}F(\tau ,X), \end{aligned}$$

is a solution to the α-SQG equation if and only if (Θ,F) solves the self-similar α-SQG equation

2.3 $$\begin{aligned} \partial _\tau \Theta +V\cdot \nabla \Theta -b\left( (a-\alpha )+X\cdot \nabla \right) \Theta =F. \end{aligned}$$

The corresponding velocities are linked by

2.4 $$\begin{aligned} v(t,x)=(abt)^{\frac{1}{a}-1}V(\tau ,X), \end{aligned}$$

that is, (*v*, *V*) are recovered from $(\theta ,\Theta )$ through the α-Biot-Savart law (1.2), respectively.

#### Proof

Firstly, we deduce the relation (2.4)

$$\begin{aligned} v(t,x)&=C_\alpha \int _{\mathbb {R}^2}\frac{(x-y)^\perp }{|x-y|^{2+\alpha }}\theta (t,y)\,\textrm{d}y =(abt)^{\frac{\alpha }{a}-1}C_\alpha \int _{\mathbb {R}^2}\frac{(x-y)^\perp }{|x-y|^{2+\alpha }}\Theta (\tau ,Y)\,\textrm{d}y\\&=(abt)^{\frac{1}{a}-1}C_\alpha \int _{\mathbb {R}^2}\frac{(X-Y)^\perp }{|X-Y|^{2+\alpha }}\Theta (\tau ,Y)\,\textrm{d}Y =(abt)^{\frac{1}{a}-1} V(\tau ,X), \end{aligned}$$

where we made the change of variables $y=(abt)^{\nicefrac {1}{a}}Y$ with $\,\textrm{d}y=(abt)^{\nicefrac {2}{a}}\,\textrm{d}Y$. On the one hand,

$$ \partial _t\theta =(abt)^{\frac{\alpha }{a}-2} \left( \partial _\tau \Theta +b(\alpha -a)\Theta -bX\cdot \nabla \Theta \right) . $$

On the other hand,

$$ v\cdot \nabla \theta = (abt)^{\frac{\alpha }{a}-2}V\cdot \nabla \Theta . $$

The last two identities imply the equivalence between (1.1) and (2.3) through the self-similar change of variables (2.1). □

#### Remark 2.1

Following the notation in [1, 31], given an object related to the α-SQG equation, the corresponding object in self-similar coordinates will be denoted by the same letter in uppercase. The only exception is the vortex (3.4). Nonetheless, notice that the case b=0 in (2.3) agrees with α-SQG in the original coordinates, while b>0 represents the equation in self-similar coordinates.

In the next proposition we show how the $W^{s,p}$ norm behaves under the self-similar change of variables (2.1). We recall that, for any $s\in \mathbb {R}$ and $1\le p\le \infty$, the fractional Sobolev space $W^{s,p}=W^{s,p}(\mathbb {R}^2)$ is defined by the normwhere $\hat{f}$ denotes the Fourier transform

$$ \hat{f}(k)=\int _{\mathbb {R}^2}f(x)e^{-ix\cdot k}\,\textrm{d}x, $$

and $\check{f}$ denotes the inverse Fourier transform. We also consider the seminormIn the Hilbert case (p=2) we will denote $H^s=W^{s,2}$ and $\dot{H}^s=\dot{W}^{s,2}$ as usual. In general, we will consider s≥0, except when dealing with the Hamiltonian.

#### Proposition 2.2

Let (θ,f) and (Θ,F) be linked by (2.1). Then,

2.5a $$\begin{aligned} \Vert \theta (t)\Vert _{\dot{W}^{s,p}}&=(abt)^{\frac{\alpha +\frac{2}{p}-s}{a}-1} \Vert \Theta (\tau )\Vert _{\dot{W}^{s,p}}, \end{aligned}$$

2.5b $$\begin{aligned} \Vert f(t)\Vert _{\dot{W}^{s,p}}&=(abt)^{\frac{\alpha +\frac{2}{p}-s}{a}-2} \Vert F(\tau )\Vert _{\dot{W}^{s,p}}. \end{aligned}$$

#### Proof

Firstly, we write θ in the Fourier side,

$$\begin{aligned} \hat{\theta }(t,k)&=\int _{\mathbb {R}^2}\theta (t,x)e^{-ix\cdot k}\,\textrm{d}x =(abt)^{\frac{\alpha }{a}-1}\int _{\mathbb {R}^2}\Theta (\tau ,X)e^{-ix\cdot k}\,\textrm{d}x\\&=(abt)^{\frac{2+\alpha }{a}-1}\int _{\mathbb {R}^2}\Theta (\tau ,X)e^{-iX\cdot K}\,\textrm{d}X =(abt)^{\frac{2+\alpha }{a}-1}\hat{\Theta }(\tau ,K), \end{aligned}$$

where $$ K=(abt)^{1/a}k. $$ Thus,

$$ g(t,k) =(abt)^{\frac{2+\alpha -s}{a}-1}G(\tau ,K), $$

where $g=|k|^s\hat{\theta }$ and $G=|K|^s\hat{\Theta }$. Analogously, we compute

$$\begin{aligned} \check{g}(t,x)&=\frac{1}{(2\pi )^2}\int _{\mathbb {R}^2}g(t,k)e^{ik\cdot x}\,\textrm{d}k =\frac{(abt)^{\frac{2+\alpha -s}{a}-1}}{(2\pi )^2}\int _{\mathbb {R}^2}G(\tau ,K)e^{ik\cdot x}\,\textrm{d}k\\&=\frac{(abt)^{\frac{\alpha -s}{a}-1}}{(2\pi )^2}\int _{\mathbb {R}^2}G(\tau ,K)e^{iK\cdot X}\,\textrm{d}K =(abt)^{\frac{\alpha {-}s}{a}-1}\check{G}(\tau ,X). \end{aligned}$$

Therefore,

$$\begin{aligned} \Vert \theta (t)\Vert _{\dot{W}^{s,p}}^p&=\int _{\mathbb {R}^2} |\check{g}(t,x)|^p\,\textrm{d}x =(abt)^{p\left( \frac{\alpha -s}{a}-1\right) } \int _{\mathbb {R}^2} |\check{G}(\tau ,X)|^p\,\textrm{d}x\\&=(abt)^{p\left( \frac{\alpha +\frac{2}{p}-s}{a}-1\right) } \int _{\mathbb {R}^2} |\check{G}(\tau ,X)|^p\,\textrm{d}X =(abt)^{p\left( \frac{\alpha +\frac{2}{p}-s}{a}-1\right) } \Vert \Theta (\tau )\Vert _{\dot{W}^{s,p}}^p. \end{aligned}$$

The proof for *f* is analogous. □

#### Definition 2.1

We say that $(\theta _0,f)$ is a *self-similar solution* to the α-SQG equation if the corresponding pair $(\bar{\Theta },F)$ given by the self-similar change of variables (2.1) is a stationary solution of the self-similar α-SQG equation, that is,

2.6 $$\begin{aligned} \bar{V}\cdot \nabla \bar{\Theta } -b\left( (a-\alpha )+X\cdot \nabla \right) \bar{\Theta } =F, \end{aligned}$$

where $\bar{V}$ is recovered from $\bar{\Theta }$ through the α-Biot-Savart law (1.2).

#### Remark 2.2

Observe that any $\bar{\Theta }$ becomes a solution by defining the force *ad hoc* as in equation (2.6). This degree of freedom is the main advantage of considering the forced equation, enabling the consideration of functions for which the stability analysis becomes feasible, although it still requires considerable effort.

In view of Proposition 2.2, the integrability conditions (1.5) and (1.7) for self-similar solutions require to take *a* in the regime

$$ 0<a<\alpha +\frac{2}{p}-s, $$

which is possible if and only if *s* satisfies

2.7 $$\begin{aligned} s<\alpha +\frac{2}{p}, \end{aligned}$$

as stated in Theorem 1.1. From now on, we consider these parameters to be fixed in this regime

2.8 $$\begin{aligned} 0<a<\varepsilon \le \alpha +\frac{2}{p}-s, \end{aligned}$$

where $0<\varepsilon <1+\alpha$ is the parameter in Theorem 3.1.

#### Corollary 2.1

Let $(\theta _0,f)$ be a self-similar solution with $(\bar{\Theta },F)$ smooth and compactly supported. It holds that

2.9a $$\begin{aligned} \Vert \theta _0(t)\Vert _{\dot{W}^{s,p}}&=(abt)^{\frac{\alpha +\frac{2}{p}-s}{a}-1} \Vert \bar{\Theta }\Vert _{\dot{W}^{s,p}}, \end{aligned}$$

2.9b $$\begin{aligned} \int _0^t \Vert f(t')\Vert _{\dot{W}^{s,p}}\,\textrm{d}t'&=\frac{(abt)^{\frac{\alpha +\frac{2}{p}-s}{a}-1}}{ab\left( \frac{\alpha +\frac{2}{p}-s}{a}-1\right) } \Vert F\Vert _{\dot{W}^{s,p}}. \end{aligned}$$

In particular, $$\theta _0|_{t=0}=0$$.

#### Proof

It follows by applying Definition 2.1 and Proposition 2.2. □

### Linear Stability Equation

In this section we derive the linear stability equation around a (self-similar) solution. Given a background steady temperature $\bar{\Theta }$, a perturbation

$$ \Theta _\epsilon =\bar{\Theta }+\epsilon \tilde{\Theta }, $$

is a second solution to the (self-similar) α-SQG equ. (2.3) with the same initial data and force if and only if the deviation $\tilde{\Theta }$ solves

2.10 $$\begin{aligned} (\partial _\tau -L_b)\tilde{\Theta } +\epsilon (\tilde{V}\cdot \nabla \tilde{\Theta })=0, \end{aligned}$$

coupled with the initial condition

2.11 $$\begin{aligned} \tilde{\Theta }|_{\tau =-\infty }=0. \end{aligned}$$

The operator $L_b$ is the linearization of the (self-similar) α-SQG equation around $\bar{\Theta }$. This can be decomposed into

2.12 $$\begin{aligned} L_b =b(a-\alpha ) +T_b+K, \end{aligned}$$

where

$$\begin{aligned} T_b\Theta&=(bX-\bar{V})\cdot \nabla \Theta ,\\ K\Theta&=-V\cdot \nabla \bar{\Theta }. \end{aligned}$$

The velocities $(V,\bar{V})$ are recovered from $(\Theta ,\bar{\Theta })$ respectively through the α-Biot-Savart law (1.2).

#### Remark 2.3

Notice that $L_b$, $T_b$ and *K* depend on *a*, α and $\bar{\Theta }$. Since the parameter *a* has already been fixed in (2.8), we omit this dependence. The dependence on α and $\bar{\Theta }$ will be made explicit in the notation when necessary.

As usual in Stability theory, as $\epsilon \rightarrow 0$ one is lead to study the linear equation

2.13 $$\begin{aligned} (\partial _\tau -L_b)\Theta ^{\text {lin}}=0, \end{aligned}$$

and seek for solutions that grow exponentially in time, that is,

2.14 $$\begin{aligned} \Theta ^{\text {lin}}(\tau ,X)=\Re (e^{\lambda \tau }W(X)), \end{aligned}$$

with $\Re \lambda >0$. For solutions of this form, equation (2.13) is equivalent to the eigenvalue problem

2.15 $$\begin{aligned} L_bW=\lambda W. \end{aligned}$$

In other words, linear instability translates into the existence of eigenvalues λ in the right half plane $\mathbb {C}_+$, and eigenfunctions *W* in some suitable Hilbert space, of the linear operator $L_b=L_{b,\alpha ,\bar{\Theta }}$.

An obvious but crucial observation is that

$$ e^{\lambda \tau } =t^{\frac{\lambda }{ab}} \rightarrow 0, $$

as $\tau \rightarrow -\infty$ (or equivalently t→0), and therefore

2.16 $$\begin{aligned} \Theta ^{\text {lin}}|_{\tau =-\infty }=0, \end{aligned}$$

which leads to non-uniqueness at the linear level.

#### Vortices

We consider the special case of radially symmetric temperatures, called *vortices*. In polar coordinates $X=Re^{i\phi }$, this corresponds to

2.17 $$\begin{aligned} \bar{\Theta }(X) =\bar{\Theta }(R). \end{aligned}$$

In Lemma 2.1, we show that the corresponding velocity $\bar{V}$, recovered from $\bar{\Theta }$ through the α-Biot-Savart law (1.2), has only an angular component $\bar{V}_\phi$. Moreover, we show that $\bar{V}_\phi$ is expressed in terms of the operator $V_{1,\alpha }$, defined below. We take the opportunity to introduce this operator more generally as $V_{n,\alpha }$, since it will appear in the eigenspace $U_n$ in the following subsection.

Given $n\in \mathbb {Z}$, we define the operator

2.18 $$\begin{aligned} V_{n,\alpha }[f](R) :=C_\alpha \int _0^\infty I_{n,\alpha }\left( \frac{R}{S}\right) f(S)S^{1-\alpha }\,\textrm{d}S, \end{aligned}$$

where $C_\alpha$ is the constant in (1.2), and $I_{n,\alpha }$ is the kernel

2.19 $$\begin{aligned} I_{n,\alpha }(\sigma ) :=\frac{1}{\alpha }\int _{-\pi }^{\pi }\frac{\cos (n\beta )}{|\sigma -e^{i\beta }|^{\alpha }}\,\textrm{d}\beta , \end{aligned}$$

whenever these expressions make sense.

##### Remark 2.4

A priori, (2.19) seems ill defined for α=0. However, an integration by parts (see (2.22)) shows that $I_{n,\alpha }$ can be expressed as

$$ I_{n,\alpha }(\sigma ) =\frac{\sigma }{n}K_{n,\alpha }(\sigma ), $$

where

$$ K_{n,\alpha }(\sigma ) :=\int _{-\pi }^{\pi }\frac{\sin (\beta )\sin (n\beta )}{|\sigma -e^{i\beta }|^{2+\alpha }}\,\textrm{d}\beta , $$

which is well defined for α=0 as well. Since this case corresponds to the 2D Euler equation, which has already been analyzed in [31], we mostly deal with (2.19) for the sake of convenience. However, at certain points, it will be more convenient to use $K_{n,\alpha }$.

##### Lemma 2.1

It holds that

$$ \bar{V}(X) =\bar{V}_\phi (R)e_\phi , \quad \text {with} \quad \bar{V}_\phi =- V_{1,\alpha }[\partial _R\bar{\Theta }], $$

where $V_{1,\alpha }$ is defined in (2.18).

##### Proof

By applying

$$ \nabla \bar{\Theta }(X) =\partial _R\bar{\Theta }(R)e^{i\phi }, $$

we compute ($$\beta =\phi -\varphi $$)

$$\begin{aligned} \bar{V}(X) =-\frac{C_\alpha }{\alpha }\int _{\mathbb {R}^2}\frac{\nabla ^\perp \bar{\Theta }(Y)}{|X-Y|^{\alpha }}\,\textrm{d}Y&=-\frac{C_\alpha }{\alpha }\int _{-\pi }^{\pi }\int _0^\infty \frac{ie^{i\varphi }\partial _R\bar{\Theta }(S)}{|Re^{i\phi }-Se^{i\varphi }|^\alpha }S\,\textrm{d}S\,\textrm{d}\varphi \\&=-ie^{i\phi }\frac{C_\alpha }{\alpha }\int _0^\infty \int _{-\pi }^{\pi } \frac{e^{-i\beta }}{|R{-}Se^{i\beta }|^\alpha }\,\textrm{d}\beta \partial _R\bar{\Theta }(S) S\,\textrm{d}S\\&=-ie^{i\phi }\frac{C_\alpha }{\alpha }\int _0^\infty \int _{-\pi }^{\pi } \frac{\cos \beta }{|R{-}Se^{i\beta }|^\alpha }\,\textrm{d}\beta \partial _R\bar{\Theta }(S) S\,\textrm{d}S. \end{aligned}$$

This concludes the proof. □

##### Corollary 2.2

It holds that (here *V* is recovered from *W* through the α-Biot-Savart law (1.2))

$$\begin{aligned} T_bW&=\left( bR\partial _R-\frac{\bar{V}_\phi }{R}\partial _\phi \right) W,\\ KW&=-V_R\partial _R\bar{\Theta }. \end{aligned}$$

##### Proof

On the one hand,

$$ X\cdot \nabla W=R\partial _R W. $$

On the other hand,

$$ \bar{V}\cdot \nabla W=\frac{\bar{V}_\phi }{R}\partial _\phi W, \quad \quad V\cdot \nabla \bar{\Theta } =V_R\partial _R\bar{\Theta }, $$

where we applied (2.17) and Lemma 2.1. □

#### Purely *n*-fold Symmetric Temperatures

In order to ensure that $\bar{\Theta }$ has finite Hamiltonian for α=0, we will work in the space $U_0$ of vortices in $L^2(\mathbb {R}^2)$ with zero-mean,

2.20 $$\begin{aligned} U_0 :=\left\{ \bar{\Theta }\in L^2 \,:\, \bar{\Theta }(X)=\bar{\Theta }(R)\, ,\ \int _0^\infty \bar{\Theta }(R)R\,\textrm{d}R=0\right\} . \end{aligned}$$

Although the zero-mean condition is not strictly necessary for α>0 for having finite Hamiltonian, it appears to be more convenient for producing unstable vortices (see Remark 3.3). In this context, given $0\ne n\in \mathbb {Z}$, it is natural to seek eigenfunctions in the space of *purely* *n*-*fold symmetric* temperatures,

2.21 $$\begin{aligned} U_n:=\{W\in L^2\,:\,W(X)=W_n(R)e^{in\phi }\}. \end{aligned}$$

Notice that any element of $U_n$ has zero-mean. Since $U_{-n}=U_n^*$, we will consider without loss of generality the case $n\in \mathbb {N}$.

##### Lemma 2.2

For every $$W\in U_n$$,

$$ V_R(X) =in\frac{V_{n,\alpha }[W_n](R)}{R}e^{in\phi }, $$

where $V_{n,\alpha }$ is defined in (2.18).

##### Proof

By writing the α-Biot-Savart law (1.2) in polar coordinates ($\beta =\phi -\varphi$)

$$\begin{aligned} V(X)&=C_\alpha \int _0^\infty \int _{-\pi }^{\pi } \frac{(Re^{i\phi }-Se^{i\varphi })i}{|Re^{i\phi }-Se^{i\varphi }|^{2+\alpha }}W(Se^{i\varphi }) \,\textrm{d}\varphi S\,\textrm{d}S\\&=ie^{i\phi } C_\alpha \int _0^\infty \int _{-\pi }^{\pi } \frac{R-Se^{-i\beta }}{|R-Se^{i\beta }|^{2+\alpha }}W(Se^{i(\phi -\beta )}) \,\textrm{d}\beta S\,\textrm{d}S, \end{aligned}$$

we get

$$\begin{aligned} V_R =\Re (Ve^{-i\phi })&=-C_\alpha \Im \int _0^\infty \int _{-\pi }^{\pi } \frac{R-Se^{-i\beta }}{|R-Se^{i\beta }|^{2+\alpha }}W(Se^{i(\phi -\beta )}) \,\textrm{d}\beta S\,\textrm{d}S\\&=-C_\alpha \int _0^\infty \int _{-\pi }^{\pi } \frac{\sin \beta }{|R-Se^{i\beta }|^{2+\alpha }}W(Se^{i(\phi -\beta )})\,\textrm{d}\beta S^2\,\textrm{d}S. \end{aligned}$$

We remark that this is the expression for the real operator $V_R$ (accordingly for $KW=-V_R\partial _R\bar{\Theta }$). Next, we consider $V_R$ acting on complex-valued *W*’s. Hence, for $W(X)=W_n(R)e^{in\phi }$, we have

$$\begin{aligned} V_R&=-e^{in\phi }C_\alpha \int _0^\infty \int _{-\pi }^{\pi } \frac{\sin \beta e^{-in\beta }}{|R-Se^{i\beta }|^{2+\alpha }}\,\textrm{d}\beta W_n(S) S^2\,\textrm{d}S \\&=ie^{in\phi }C_\alpha \int _0^\infty K_{n,\alpha }\left( \frac{R}{S}\right) W_n(S)S^{-\alpha }\,\textrm{d}S, \end{aligned}$$

where

2.22 $$\begin{aligned} \begin{aligned} K_{n,\alpha }(\sigma ) =\int _{-\pi }^{\pi }\frac{\sin \beta \sin (n\beta )}{|\sigma -e^{i\beta }|^{2+\alpha }}\,\textrm{d}\beta&=-\frac{1}{\alpha \sigma } \int _{-\pi }^{\pi }\partial _\beta \left( \frac{1}{|\sigma -e^{i\beta }|^{\alpha }}\right) \sin (n\beta )\,\textrm{d}\beta \\&=\frac{n}{\alpha \sigma } \int _{-\pi }^{\pi }\frac{\cos (n\beta )}{|\sigma -e^{i\beta }|^{\alpha }}\,\textrm{d}\beta =\frac{n}{\sigma }I_{n,\alpha }(\sigma ). \end{aligned} \end{aligned}$$

This concludes the proof. □

##### Corollary 2.3

For every $W\in U_n$, it holds that

$$\begin{aligned} T_bW&=\left( bR\partial _R-in\frac{\bar{V}_\phi }{R}\right) W_n e^{in\phi },\\ KW&=-in\frac{V_{n,\alpha }[W_n]}{R}\partial _R\bar{\Theta }e^{in\phi }. \end{aligned}$$

##### Proof

It follows from Corollary 2.2, definition (2.21) and Lemma 2.2. □

Therefore, the eigenvalue problem $L_bW=\lambda W$ for $W\in U_n$ can be rewritten as

2.23 $$\begin{aligned} b(a-\alpha )W_n +\left( bR\partial _R-in\frac{\bar{V}_\phi }{R}\right) W_n -in\partial _R\bar{\Theta }\frac{V_{n,\alpha }[W_n]}{R} =\lambda W_n, \end{aligned}$$

which we will refer to as the *linear stability equation*. We recall that the case b=0 corresponds to the original coordinates, while b>0 corresponds to the self-similar coordinates.

##### Remark 2.5

As mentioned, for b=0, equation (2.23) corresponds to the stability equation in the original coordinates. In particular, for α=0, this represents the Rayleigh stability equation for the 2D Euler equation at the vorticity level.

##### Definition 2.2

We say that the vortex $\bar{\Theta }$ is *unstable* if, for some $n\in \mathbb {N}$, there exists $0\ne W\in U_n$ satisfying $L_{0,\bar{\Theta }}W=\lambda W$ with $\Re \lambda >0$. Similarly, we say that $\bar{\Theta }$ is *self-similarly unstable* if, for some $n\in \mathbb {N}$ and b>0, there exists $0\ne W\in U_n$ satisfying $L_{b,\bar{\Theta }}W=\lambda W$ with $\Re \lambda >0$.

### Non-Linear Stability Equation

Let us assume that such a (self-similarly) unstable vortex $\bar{\Theta }$ exists. We decompose the deviation into

$$ \tilde{\Theta }=\Theta ^{\text {lin}} +\epsilon \Theta ^{\text {cor}}, $$

where $\Theta ^{\text {lin}}(\tau ,X)=\Re (e^{\lambda \tau }W(X))$, and $\Theta ^{\text {cor}}$ is the non-linear correction, to be determined. Namely, $\Theta ^{\text {cor}}$ must satisfy the equation

2.24 $$\begin{aligned} (\partial _\tau - L_b)\Theta ^{\text {cor}} +\underbrace{(V^{\text {lin}}+\epsilon V^{\text {cor}})\cdot \nabla (\Theta ^{\text {lin}}+\epsilon \Theta ^{\text {cor}})}_{\mathcal {F}} =0, \end{aligned}$$

which we will refer to as the *non-linear stability equation*, coupled with the initial condition

2.25 $$\begin{aligned} \Theta ^{\text {cor}}|_{\tau =-\infty }=0. \end{aligned}$$

If we interpret $\mathcal {F}$ as a forcing term, since the linear part decays as $e^{\Re \lambda \tau }$ and the contribution of the quadratic term is expected to be negligible for short times, one can naively expect to gain a slightly faster exponential decay by exploiting the Duhamel formula. We recall that the case b=0 corresponds to the original coordinates, while b>0 corresponds to the self-similar coordinates.

#### Definition 2.3

We say that the (self-similarly) unstable vortex $\bar{\Theta }$ is also *(self-similarly) non-linearly unstable* if there exists $\Theta ^{\text {cor}}$ solving (2.24) and (2.25) with

2.26 $$\begin{aligned} \Vert \Theta ^{\text {cor}}(\tau )\Vert _{L^2} =o(e^{\Re \lambda \tau }), \end{aligned}$$

as $$\tau \rightarrow -\infty $$.

#### Space of *n*-fold Symmetric Temperatures

In the previous sections we considered the linearization $L_b$ acting on the invariant subspace $U_n$ of purely *n*-fold symmetric temperatures. However, for the non-linear instability we will need to take into account the quadratic term, which introduces multiples of the original frequency *n*. For this reason, it is convenient to analyze $L_b$ within the larger space $L_n^2$ of temperatures $\Theta \in L^2$ which have zero mean and are *n*-fold symmetric

2.27 $$\begin{aligned} \Theta (X)=\Theta (e^{\frac{2\pi i}{n}}X). \end{aligned}$$

By writing the Fourier expansion of Θ in polar coordinates $X=Re^{i\theta }$,

$$ \Theta (X)=\sum _{k\in \mathbb {Z}}\Theta _k(R)e^{ik\theta }, \quad \quad \Theta _k(R) =\frac{1}{2\pi }\int _0^{2\pi }\Theta (Re^{i\theta })e^{-ik\theta }\,\textrm{d}\theta , $$

it follows that the *n*-fold symmetry (2.27) is equivalent to the vanishing of the indices *k* that are not multiples of *n*, and the zero mean condition for k=0

$$ \int _0^\infty \Theta _0(R)R\,\textrm{d}R=0. $$

Therefore, we can decompose $L_n^2$ into the orthogonal direct sum

2.28 $$\begin{aligned} L_n^2=\bigoplus _{j\in \mathbb {Z}}U_{jn} \end{aligned}$$

of the invariant subspaces $U_0$ and $U_{jn}$ given in (2.20) and (2.21), respectively. More precisely, by the Plancherel identity

2.29 $$\begin{aligned} \Vert \Theta \Vert _{L^2}^2 =2\pi \sum _{j\in \mathbb {Z}} \int _0^\infty |\Theta _{jn}(R)|^2R\,\textrm{d}R, \end{aligned}$$

we consider sums of elements $\Theta _{jn}$ for which (2.29) is finite.

## Sketch of the Proof

In this section, we prove Theorem 1.2, as well as the following stronger version of Theorem 1.1.

### Theorem 3.1

(Refined Sobolev non-uniqueness) Let $0\le \alpha \le 1$ and $0<\varepsilon <1+\alpha$. There exist T>0, a force *f* and uncountably many solutions $\theta _\epsilon$ to the α-SQG equation with forcing *f* and initial datum $\theta ^\circ =0$ such that

3.1 $$\begin{aligned} f\in L^1([0,T],W^{s,p}\cap \dot{H}^{\frac{\alpha -2}{2}}) \quad \text {and} \quad \theta _\epsilon \in L^\infty ([0,T],W^{s,p}\cap \dot{H}^{\frac{\alpha -2}{2}}) \end{aligned}$$

for every s≥0 and $1\le p\le \infty$ satisfying $s\le \alpha +\frac{2}{p}-\varepsilon$.

### Remark 3.1

**Fig. 1:**
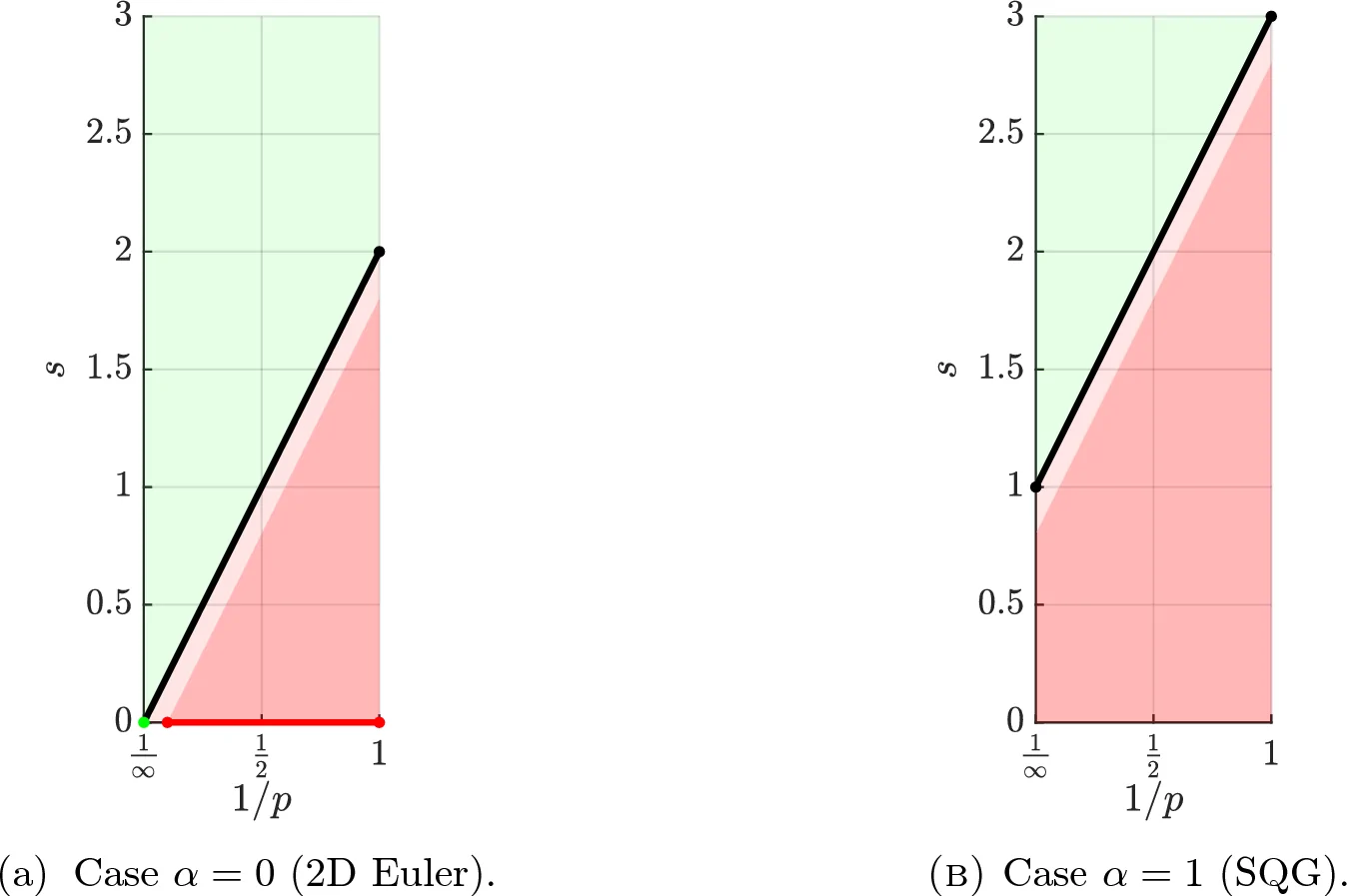
Representation of the Sobolev spaces $W^{s,p}$ in terms of the variables 1/*p* and *s*. The green region corresponds to the well-posedness regime s>α+2/p. The black segment corresponds to the critical line s=α+2/p. The light red region corresponds to the (expected) non-uniqueness regime s<α+2/p. The red area $s\le \alpha +2/p-\varepsilon$ corresponds to the non-uniqueness with forcing, as established in Theorem 3.1, where ε>0 is arbitrarily small. The red segment corresponds to Vishik’s non-uniqueness theorem (α=s=0). In this context, the green point (1/p,s)=(0,0) corresponds to the Yudovich well-posedness class

The improvement is that, in Theorem 3.1, the force and the solutions belong simultaneously to all the Sobolev spaces corresponding to the red region in Figure 1. The condition $\varepsilon < 1 + \alpha$ will appear frequently in the proofs. Moreover, it guarantees that $\theta \in L^2$, which is sufficient to properly define the weak formulation at the level of θ.

We take the opportunity to recall here Vishik’s result to facilitate comparison. Notice that, for α=0, the solutions can be extended globally in time and thus (3.1) hold for all T<∞.

### Theorem 3.2

(Vishik’s non-uniqueness theorem) Let 2<p<∞. There exists a force

3.2 $$\begin{aligned} f\in L^1([0,\infty ),\dot{H}^{-1}\cap L^1\cap L^p), \end{aligned}$$

such that there are uncountably many solutions

3.3 $$\begin{aligned} \theta _\epsilon \in L^\infty ([0,\infty ),\dot{H}^{-1}\cap L^1\cap L^p), \end{aligned}$$

to the 2D Euler equation starting from $\theta ^\circ =0$.

We begin with a general overview of the strategy. In short, the key observation is that a vortex $\bar{\Theta }$ that is self-similarly non-linearly unstable implies non-uniqueness. We first explain this idea succinctly and then outline the steps for constructing the vortex.

Firstly, we take the main solution $\theta _0$ as the self-similar vortex corresponding to $\bar{\Theta }$. According to Definition 2.1, this means that the pair $(\theta _0,f)$ is given by the self-similar change of variables

3.4a $$\begin{aligned} \theta _0(t,x)&=(abt)^{\frac{\alpha }{a}-1}\bar{\Theta }(X), \end{aligned}$$

3.4b $$\begin{aligned} f(t,x)&=(abt)^{\frac{\alpha }{a}-2}F(X), \end{aligned}$$

in terms of $\bar{\Theta }$. The force *F* is defined *ad-hoc* by

3.5 $$\begin{aligned} F=-b\left( (a-\alpha )+R\partial _R\right) \bar{\Theta }. \end{aligned}$$

### Remark 3.2

In Sect. 5, we choose a regularization parameter ε for $\bar{\Theta }$, so that $\partial _R \bar{\Theta }$ scales as $\varepsilon ^{-1}$. In Sect. 6, we show that the parameter *b*, which characterizes the separation rate of the nonunique solutions, can be chosen arbitrarily small. It is worth noting that making *F* small in (3.5) requires a trade-off between ε and *b*. This should be compared with the scenario in [2].

Secondly, according to Definition 2.2, for some $n\in \mathbb {N}$ and b>0, there exists $0\ne W\in U_n$ satisfying $L_bW=\lambda W$ with $\Re \lambda >0$. We define

$$ \theta ^{\text {lin}}(t,x) =(abt)^{\frac{\alpha }{a}-1}\Theta ^{\text {lin}}(\tau ,X), $$

where

$$ \Theta ^{\text {lin}} =\Re (e^{\lambda \tau }W). $$

Thirdly, according to Definition 2.3, there exists a correcting term

$$ \theta ^{\text {cor}}(t,x) =(abt)^{\frac{\alpha }{a}-1}\Theta ^{\text {cor}}(\tau ,X), $$

with $\Theta ^{\text {cor}}$ satisfying (2.24) and

$$ \Theta ^{\text {cor}}=o(e^{\lambda \tau }). $$

Therefore, the temperature

$$ \theta _\epsilon =\theta _0+\epsilon \theta ^{\text {lin}}+\epsilon ^2 \theta ^{\text {cor}}, $$

yields a different solution to the α-SQG equation for $\epsilon \ne 0$. In Sect. 3.1, we rigorously prove Theorem 3.1 as a consequence of the properties of $\bar{\Theta }$, $\Theta ^{\text {lin}}$, and $\Theta ^{\text {cor}}$, established in Theorems 3.4 and 3.5, along with the Sobolev scaling given in Proposition 2.2. Similarly, in Sect. 3.2, we prove Theorem 1.2 as a consequence of Theorems 3.3 and 3.5.

As introduced in Sect. 1.2, the proof of the existence of a self-similarly non-linearly unstable vortex $\bar{\Theta }$ is divided into three main steps: 1. Eulerian, 2. Self-similar, and 3. Non-linear instability. Furthermore, Step 1. is further divided into two intermediate steps: 1.1. Construction of a piecewise constant unstable vortex and 1.2. Regularization.

Next, we outline these steps, which allow us to establish Theorems 3.3–3.5. The proofs of these theorems form the core of this work and will be presented rigorously in Sects. 4–7.

### Step 1. Instability in the Eulerian coordinates

The first step involves constructing an unstable vortex that is smooth and compactly supported in the original coordinates. In other words, we must solve the stability equation (2.23) for b=0. Since the construction of these vortices has its own physical and mathematical interest, and in order to maintain consistency in notation, we will adopt the convention of using lowercase letters until we return to the self-similar variables.

Let $\bar{\theta }$ be a vortex with corresponding velocity $\bar{v}$. By recalling Lemmas 2.1 and 2.2, the stability equation (2.23) for b=0 and $w\in U_n$ reads as

3.6 $$\begin{aligned} \frac{1}{r}\int _0^\infty \left( I_{n,\alpha }\left( \frac{r}{s}\right) w_n(s)\partial _r\bar{\theta }(r) -I_{1,\alpha }\left( \frac{r}{s}\right) w_n(r)\partial _r\bar{\theta }(s)\right) s^{1-\alpha }\,\textrm{d}s =zw_n(r),\nonumber \\ \end{aligned}$$

where we have replaced $\lambda =-in C_\alpha z$, being $C_\alpha$ the constant in the α-Biot-Savart law (1.2). Observe that $\Re \lambda >0$ translates to ℑz>0. Therefore, (3.6) implies that $w_n(r)$ must vanish wherever $\partial _r\bar{\theta }(r)=0$. Thus, we can write

$$ w_n=h\partial _r\bar{\theta }, $$

in terms of some profile *h*. For that *h*, the equation (3.6) reads as

3.7 $$\begin{aligned} \frac{1}{r}\int _0^\infty \left( I_{n,\alpha }\left( \frac{r}{s}\right) h(s) -I_{1,\alpha }\left( \frac{r}{s}\right) h(r)\right) \partial _r\bar{\theta }(s)s^{1-\alpha }\,\textrm{d}s =zh(r), \quad r{\in }\textrm{supp}(\partial _r\bar{\theta }).\nonumber \\ \end{aligned}$$

Thus, linear instability reduces to finding a vortex $\bar{\theta }$ that admits a nontrivial eigenpair (*h*, *z*) of (3.7) with ℑz>0. We will show that this is indeed the case and therefore land in our first main result (recall Definition 2.2).

#### Theorem 3.3

For every n≥2, there exists an unstable vortex $\bar{\Theta }\in C_c^\infty \cap U_0$ such that the corresponding eigenfunction satisfies $W\in C_c^\infty \cap U_n$.

Our proof of Theorem 3.3 is split into two steps.

### Step 1.1. Piecewise constant unstable vortex

In Sect. 4 we construct an unstable vortex of the form

$$ \bar{\theta } =c 1_{[0,r_1)} - 1_{[r_1,r_2)}, $$

in terms of some parameters $0<r_1<r_2<\infty$ and c>0, to be determined. For the sake of simplicity we fix

$$ r_1=\sigma , \quad \quad r_2=1, $$

for some 0<σ<1, to be determined. Firstly, we choose *c* making the mean of $\bar{\theta }$ equal zero, and thus $\bar{\theta }\in U_0$. This is equivalent to

3.8 $$\begin{aligned} (1+c)\sigma ^2=1. \end{aligned}$$

Secondly, since

$$ \textrm{supp}(\partial _r\bar{\theta })=\{\sigma ,1\}, $$

the linear stability equ.(3.7) turns into two conditions for the vector h=(h(σ),h(1)). This can be written as a linear system

3.9 $$\begin{aligned} A h=zh, \end{aligned}$$

where the matrix $A\in \mathbb {R}^{2\times 2}$ depends on the parameter σ and the frequency *n*, namely

3.10 $$\begin{aligned} A= \left[ \begin{array}{cc} \sigma ^{-(2+\alpha )}J_{n,\alpha }(1)-\sigma ^{-1}I_{1,\alpha }(\sigma ) &  \sigma ^{-1}I_{n,\alpha }(\sigma ) \\ -\sigma ^{-1} I_{n,\alpha }(\sigma ) &  -J_{n,\alpha }(1) +\sigma ^{-1} I_{1,\alpha }(\sigma ) \end{array} \right] , \end{aligned}$$

with $J_{n,\alpha }=I_{1,\alpha }-I_{n,\alpha }$. By linear algebra, there exists an eigenvector h≠0 with corresponding eigenvalue *z* if and only if *z* is a root of the characteristic polynomial det(A-z).

For the 2D Euler equation (α=0) the coefficients of the characteristic polynomial can be computed explicitly. This allows us to obtain the existence of a non-trivial *h* by an elementary explicit computation. The case α>0 is considerably more intricate since the coefficients of the characteristic polynomial are not explicit, being given in terms of the integral $I_{n,\alpha }$ evaluated at σ and 1. Furthermore, the situation is more delicate for SQG as $I_{n,1}$ diverges at 1. Fortunately, in this case there is an extra cancellation in $J_{n,\alpha }$ that allows us to consider not only SQG but also the complete regime $0\le \alpha <2$. In Sect. 4.1, we explicitly compute certain values and derive the precise asymptotics of $I_{n,\alpha }$ in terms of the Gamma function. These results allow us to show that the discriminant $\Delta (\sigma )$ of the characteristic polynomial satisfies (see Proposition 4.1)

$$ \Delta (1)=\Delta '(1)=0, \quad \quad \Delta ''(1)<0, $$

for all $0\le \alpha <2$ and n≥2. Therefore, $\Delta (\sigma )<0$ for some 0<σ<1, and thus ℑz>0.

#### Remark 3.3

The zero-mean condition is imposed in the 2D Euler equations to ensure that the Hamiltonian remains finite. Although this condition is not strictly necessary for α>0, it can be shown that the discriminant for a general vortex satisfies $\Delta (1) \ge 0$ and vanishes if and only if the vortex has zero mean. Therefore, to complete our argument, it is convenient to work within the class $U_0$ for the full range of α values.

### Step 1.2. Regularization

In Sect. 5 we prove that there exists a smooth vortex $\bar{\theta }^\varepsilon$, obtained by suitably regularizing $\bar{\theta }$ from Step 1.1, which is also unstable for some small ε>0. Similarly to Step 1.1, now we need to solve the linear stability equ. (3.7) in the intervals $B_\varepsilon (r_j)$ for j=1,2. To this aim, we first rescale variables around $r_j$ writing $r=r_j+\varepsilon \rho$ with $\rho \in I=(-1,1)$. Next, we make the asymptotic expansions for eigenfunctions and eigenvalues. Namely, we write

$$ h^\varepsilon (r) =h(r_j)+\varepsilon g_j(\rho ), \quad \quad z^\varepsilon =z+\varepsilon y, $$

for some profiles $g=(g_1,g_2)\in L^2(I)^2$, and a constant $y\in \mathbb {C}$, to be determined. The expansion of the operator

$$A^\varepsilon g =\frac{1}{r}\int _0^\infty \left( I_{n,\alpha }\left( \frac{r}{s}\right) g(s) -I_{1,\alpha }\left( \frac{r}{s}\right) g(r)\right) \partial _r\bar{\theta }^\varepsilon (s)s^{1-\alpha }\,\textrm{d}s, $$

requires a more involved analysis compared to the one presented in [31]. We split $A^\varepsilon$ into

$$ A^\varepsilon =A_0^\varepsilon +A_1^\varepsilon . $$

On the one hand, the regular part $A_0^\varepsilon$ can be expanded uniformly in α, namely (see Lemma 5.3)

$$ A_0^\varepsilon =A_0+\varepsilon B_0, $$

for some linear operator $B_0=B_0^\varepsilon$ bounded in $L^2(I)^2$. On the other hand, the singular part $A_1^\varepsilon$ can be expanded for α<1 as (see Lemma 5.4)

3.11 $$\begin{aligned} A_1^\varepsilon =A_1+\frac{\varepsilon ^{1-\alpha }}{1-\alpha } B_1, \end{aligned}$$

for another linear operator $B_1=B_1^\varepsilon$ bounded in $L^2(I)^2$. Therefore, for α<1 we can follow [31]. We recall the argument here for the reader’s convenience. The linear stability equation

$$ (A^\varepsilon -z^\varepsilon )h^\varepsilon =0, $$

is rewritten as

3.12 $$\begin{aligned} (A-z)g =(y-B_0)h+ \left( \varepsilon y-\frac{\varepsilon ^{1-\alpha }}{1-\alpha }B\right) g, \end{aligned}$$

where

$$ A=A_0+A_1, \quad \quad B=(1-\alpha )\varepsilon ^\alpha B_0+B_1. $$

Now the first step consists in “inverting” the operator (A-z). Unfortunately, since $z,z^*$ are the eigenvalues of *A*, the kernel and the image of (A-z) are given by $\textrm{Ker}(A-z)=\textrm{span}(h)$ and $\textrm{Im}(A-z)=\textrm{span}(h^*)$. We bypass this obstacle by choosing *y* in such a way that the right hand side of (3.12) becomes “parallel” to $h^*$ (see Lemma 5.5). This allows rewriting the equation (3.12) appropriately to apply a fixed-point argument (see Proposition 5.1).

Notice that the expansion (3.11) degenerates as $\alpha \rightarrow 1$, inverting the roles of the main term and the error. In fact, we prove that, for α=1 (see Lemma 5.6),

$$ A_1^\varepsilon =(\log \varepsilon )A_1+B_1, $$

where now

$$ (A_1g)_j = \frac{2c_j}{r_j} \left( g_j-\int _{-1}^{1}g_j\eta \,\textrm{d}\rho \right) $$

and $B_1=B_1^\varepsilon$ is a linear operator bounded in $L^2(I)^2$. Here, η is a suitably chosen mollifier (see Lemma 5.1). By decomposing

$$\begin{aligned} g=\mu +\frac{f}{\log \varepsilon }, \end{aligned}$$

where $\mu \in \mathbb {C}^2$ and $f\in L^2(I)^2$, the stability equation takes the following form (see Corollary 5.3)

$$\begin{aligned} A_1f =(y-B_0)h-(A_0-z)\mu +\varepsilon (y-B_0)\mu +\frac{1}{\log \varepsilon }((z-B_1)+\varepsilon y)f. \end{aligned}$$

Thus, in order to invert $A_1$, we now need to ensure that the right-hand side is compatible with the zero-mean condition of $A_1$. By selecting *y* and μ appropriately, we show that this condition holds (see Lemma 5.7). Finally, we apply a fixed-point argument (see Proposition 5.2).

Once ε>0 is fixed, Theorem 3.3 holds for

$$ \bar{\Theta }=\bar{\theta }^\varepsilon . $$

From now on $\bar{\Theta }\in C_c^\infty \cap U_0$ is fixed and thus we will omit ε for the sake of simplicity. Finally, we check that the eigenfunction satisfies $W\in C_c^\infty \cap U_n$ in Proposition 5.4.

### Step 2. Instability in self-similar coordinates

In order to prove that the unstable vortex $\bar{\Theta }$ is also self-similarly unstable, we follow Vishik’s argument. This requires decomposing the operator $L_b$ acting on $L_n^2$, as

$$ L_b=A_b+C, $$

where $(A_b)$ is a family of linear operators that generate contraction semigroups and possess certain continuity with respect to the parameter *b*, and *C* is compact (See Proposition 6.2). By classical operator theory, this implies that the spectrum $\sigma (L_b)$ satisfies that, for any b≥0 and w>0,

$$ \sigma (L_b)\cap \{\Re \lambda >w\} $$

is finite and consists of isolated eigenvalues. Now, Step 1 provides an eigenvalue $\lambda _0$ with positive real part for b=0. Then, using the continuity with respect to the parameter *b*, it is possible to show that there must also be eigenvalues $\lambda _b$ (of $L_b$) near $\lambda _0$ for sufficiently small b>0.

For the 2D Euler equation (α=0) this argument is applied to the decomposition

$$ A_b=b(a-\alpha ) + T_b, \quad \quad C=K, $$

where $T_b$ and *K* are defined in (2.12). In this work, we check that *K* remains compact for all $0\le \alpha <1$ (see Lemma 6.5) and thus Vishik’s argument can be applied. For α=1, the operator *K* is bounded but not compact. We bypass this obstacle by decomposing *K* into (see Proposition 6.3)

K=S+C,

where the operator *S* is skew-adjoint, thus preserving the growth of the semigroup, and the operator *C* is a commutator that can be shown to be compact (see Lemma 6.6). Therefore, we can apply Vishik’s argument to the decomposition

$$ A_b=b(a-\alpha ) + T_b +S, \quad \quad C=K-S. $$

As a consequence, we obtain the following result for $0\le \alpha \le 1$ (recall Definition 2.2):

#### Theorem 3.4

For every $m\in \mathbb {N}$, there exist b>0 and j≠0 such that the vortex $\bar{\Theta }$ from Theorem 3.3 is also self-similarly unstable and the corresponding eigenfunction satisfies $W\in C_c^{m}\cap U_{jn}$.

The properties of the eigenfunction are proved in Proposition 6.4. Indeed, this proposition states that $W\in C_c^{m+2}$, as we address the possible loss of derivatives in the next step.

### Step 3. Non-linear instability

The last step entails proving that $\bar{\Theta }$ is also non-linearly unstable. To this end, it suffices to prove that there exists $\Theta ^{\text {cor}}$ solving (2.24) and (2.25) with

3.13 $$\begin{aligned} \Vert \Theta ^{\text {cor}}(\tau )\Vert _{H_\omega ^m}\le e^{(1+\delta _0)\Re \lambda \tau }, \end{aligned}$$

for all $-\infty <\tau \le \bar{\tau }$, for some $\delta _0>0$ and $\bar{\tau }>-\infty$. Notice that we have replaced the $L^2$ space in (2.26) with the stronger weighted Hilbert space $H_\omega ^m$ given by the norm

$$ \Vert f\Vert _{H_\omega ^m} :=\Vert f\omega \Vert _{L^2} +\sum _{0<|K|\le m}\Vert \partial _X^K f\omega \Vert _{L^2}, $$

where ω is the standard radial weight

$$ \omega (X) =\langle X\rangle ^2 =1+R^2. $$

Moreover, we restrict $H_\omega ^m$ to the space of *n*-fold symmetric temperatures, that is, $H_\omega ^m\subset L_n^2$. The purpose of introducing the weight ω is to control our solutions in general Sobolev spaces by taking *m* big enough (see Lemma 7.6). In the remainder of this section, we discuss the strategy for proving (3.13). The proof starts by making some a priori energy estimates. Namely, the Duhamel formula

$$ \Theta ^{\text {cor}}(\tau ) = -\int _{-\infty }^\tau e^{(\tau -\tau ')L_b} \mathcal {F}(\tau ')\,\textrm{d}\tau ', $$

where recall

$$ \mathcal {F} =(V^{\text {lin}}+\epsilon V^{\text {cor}}) \cdot \nabla (\Theta ^{\text {lin}}+\epsilon \Theta ^{\text {cor}}), $$

gives that

$$ \Vert \Theta ^{\text {cor}}(\tau )\Vert _{L^2}\le e^{2 \Re \lambda \tau }, $$

over the time interval for which

3.14 $$\begin{aligned} \Vert V^{\text {cor}}(\tau )\Vert _{L^\infty }\le e^{\Re \lambda \tau }, \quad \quad \Vert \nabla \Theta ^{\text {cor}}(\tau )\Vert _{L^2}\le e^{\Re \lambda \tau }. \end{aligned}$$

This requires choosing *m* so that $\Vert \Theta ^{\text {cor}}\Vert _{H_\omega ^m}$ controls $\Vert V^{\text {cor}}\Vert _{L^\infty }$ and $\Vert \nabla \Theta ^{\text {cor}}\Vert _{L^2}$. Hence, at this point, it would suffice to take m>1. Note that we need to prove that $\Theta ^{\text {cor}}$ actually exists in the space $C((-\infty ,\bar{\tau }];H_{\omega }^m)$. Following the approach in [2, 31, 129, 130], we initialize the (self-similar) α-SQG equation at time τ=-k, for which both the initial data

$$ \bar{\Theta }+\epsilon \Theta ^{\text {lin}}|_{\tau =-k}, $$

and the forcing term are sufficiently smooth to define a (local in time) solution

$$ \Theta _\epsilon =\bar{\Theta }+\epsilon \Theta ^{\text {lin}} +\epsilon ^2\Theta ^{\text {cor}}. $$

In other words, for every $k\in \mathbb {N}$, we consider the unique solution $\Theta _k^{\text {cor}}$ to (2.24) coupled with the initial condition (instead of (2.25))

$$ \Theta _k^{\text {cor}}|_{\tau =-k} =0. $$

Thus, we need $H_{\omega }^m$ to be a well-posedness function space for α-SQG, which requires m>1+α. Consequently, we obtain that (3.13) holds in this space, at least on a time interval $[-k,\tau _k]$.

To prove that the time of existence $\tau _k$ does not diverge to -∞ when $k\rightarrow \infty$, a bootstrapping argument is used in [2, 31, 129, 130]. As noted in the previous works, the bootstrapping argument does not work in Cartesian coordinates, but it does in polar coordinates, provided that the partial derivatives are estimated in the correct order. In this work, we realize that these inductive energy estimates can be extended to spaces with higher-order derivatives.

To this end, we need to express Cartesian derivatives in terms of polar coordinates. In fact, we use the relation

$$ \partial _X^K f =\sum _{0<|J|\le |K|}p_J^K\frac{\partial _R^{j_1}\partial _\phi ^{j_2}f}{R^{|K|-j_1}}, $$

where $p_J^K=p_J^K(\cos \phi ,\sin \phi )$ are polynomials (see Appendix A.2). These partial derivatives need to be estimated in the following order

$$ \begin{array}{cccc} \displaystyle \frac{\partial _\phi }{R^m} &  \displaystyle \frac{\partial _R}{R^{m-1}} &  &  \\ \displaystyle \frac{\partial _\phi ^2}{R^m} &  \displaystyle \frac{\partial _R\partial _\phi }{R^{m-1}} &  \displaystyle \frac{\partial _R^2}{R^{m-2}} &  \\ \displaystyle \frac{\partial _\phi ^3}{R^m} &  \displaystyle \frac{\partial _R\partial _\phi ^2}{R^{m-1}} &  \displaystyle \frac{\partial _R^2\partial _\phi }{R^{m-2}} &  \displaystyle \frac{\partial _R^3}{R^{m-3}} \\ \cdots \end{array} $$

This suggests to consider the space $Y^m\subset L_n^2$ endowed with the norm

$$ \Vert f\Vert _{Y^m} :=\Vert f\omega \Vert _{L^2} +\sum _{0<|J|\le m}\left\| \frac{\partial _R^{j_1}\partial _\phi ^{j_2}f}{R^{m-j_1}}\omega \right\| _{L^2}, $$

which turns out to be equivalent to the norm (see Lemma 7.5)

$$ \Vert f\Vert _{Z^m} :=\Vert f\omega \Vert _{L^2} +\sum _{0<|K|\le m}\left\| \frac{\partial _X^K f}{R^{m-|K|}}\omega \right\| _{L^2}. $$

Notice that, although $Y^1=H_\omega ^1$, in general $Y^m\subsetneq H^m_\omega$ for m>1+α due to the weight at the origin (see Lemma 7.4).

In the previous works for the 2D Euler equation [2, 31, 129, 130], the energy estimates were conducted for the global smooth solution starting at time τ=-k. However, for α>0 the lack of global well-posedness makes it necessary to regularize the system in order to justify the energy estimates. Furthermore, standard regularization using mollifiers does not fit well with the weighted energy space $Y^m$. Instead, we used the regularization that comes from linearizing the equation. We consider the following linearized system. Starting from $\Theta _k^{(0)}=0$, we define recursively $\Theta _k^{(q)}$, for every $q\in \mathbb {N}$, as the solution to

$$ (\partial _\tau - L_b)\Theta _k^{(q)} +\underbrace{(V^{\text {lin}}+\epsilon V_k^{(q-1)})\cdot \nabla (\Theta ^{\text {lin}}+\epsilon \Theta _k^{(q)})}_{\mathcal {F}_k^{(q)}} =0. $$

This iteration defines a sequence of global solutions, which can be shown to lie in $Y^{m+1}$ (see Proposition 7.1). Then, we apply the inductive energy estimates on $Y^m$ to gain extra decay (see Proposition 7.3). With this, we deduce that the time of existence in our iteration scheme does not collapse (see Corollary 7.1). Finally, by a standard argument, we verify that the sequence converges to a solution as $q \rightarrow \infty$ and later $k \rightarrow \infty$. We obtain the following result (recall Definition 2.3):

#### Theorem 3.5

The vortex $\bar{\Theta }$ from Theorem 3.3 (respectively Theorem 3.4) is also (self-similarly) non-linearly unstable. Moreover, there exist $\delta _0>0$ and $\bar{\tau }>-\infty$ such that the correcting term is *n*-fold symmetric and satisfies $\Theta ^\text {cor}\in C((-\infty ,\bar{\tau }],H_{\omega }^m\cap \dot{H}^{\frac{\alpha -2}{2}})$ with

$$ \Vert \Theta ^{\text {cor}}(\tau )\Vert _{H_\omega ^m}\le e^{(1+\delta _0)\Re \lambda \tau } $$

for all $$-\infty <\tau \le \bar{\tau }$$.

### Proof of Theorem 3.1

Coming back to the original coordinates, Theorems 3.4 and 3.5 imply that the temperature

$$ \theta _\epsilon =\theta _0 +\epsilon \theta ^{\text {lin}} +\epsilon ^2\theta ^{\text {cor}}, $$

where we recall that

$$\begin{aligned}&\theta _0(t,x) =(abt)^{\frac{\alpha }{a}-1}\bar{\Theta }(X), \quad \quad \theta ^{\text {lin}}(t,x) =(abt)^{\frac{\alpha }{a}-1}\Theta ^{\text {lin}}(\tau ,X), \\&\theta ^{\text {cor}}(t,x) =(abt)^{\frac{\alpha }{a}-1}\Theta ^{\text {cor}}(\tau ,X) \end{aligned}$$

is a solution to the α-SQG equation with the same initial datum $\theta ^\circ =0$ and (radial) force

$$ f(t,x) =-b(abt)^{\frac{\alpha }{a}-2}\left( (a-\alpha )+R\partial _R\right) \bar{\Theta }(R). $$

By applying the regularity obtained in Theorems 3.4 and 3.5, the Sobolev scaling (Proposition 2.2) and the embedding $H_{\omega }^m\hookrightarrow W^{s,p}$ (Lemma 7.6) applied to $s\le \alpha +\frac{2}{p}-\varepsilon \le 3\le m-2$, we get

$$\begin{aligned}&\Vert \theta ^{\text {lin}}(t)\Vert _{\dot{W}^{s,p}} = (abt)^{\frac{\alpha +\frac{2}{p}-s}{a}-1}\Vert \Re (e^{\lambda \tau }W)\Vert _{\dot{W}^{s,p}}, \\  &\Vert \theta ^{\text {cor}}(t)\Vert _{\dot{W}^{s,p}} =(abt)^{\frac{\alpha +\frac{2}{p}-s}{a}-1}o(e^{\Re \lambda \tau }). \end{aligned}$$

These equalities, in combination with (2.8) and Corollary 2.1, imply (3.1) with $T=e^{ab\bar{\tau }}$. By the reverse triangle inequality for p=2 and s=0, we deduce that for different values of ϵ, we obtain different solutions

$$ (abt)^{1-\frac{\alpha +1}{a}}e^{-\Re \lambda \tau }\Vert (\theta _\epsilon -\theta _{\bar{\epsilon }})(t)\Vert _{L^2} \ge |\epsilon -\bar{\epsilon }| (\Vert \Re (e^{i\Im \lambda \tau }W)\Vert _{L^2} -o(1)). $$

If $\Im \lambda \ne 0$, the right-hand side is positive in a sequence of times $\tau _k^{(q)}=\tau _0-\frac{2\pi k}{\Im \lambda }\rightarrow -\infty$ as $k\rightarrow \infty$. If $\Im \lambda =0$, we can assume from the beginning that *W* is real-valued. Otherwise, it would suffice to take its imaginary part instead. This allows us to conclude that $\theta _\epsilon \ne \theta _{\bar{\epsilon }}$ whenever $\epsilon \ne \bar{\epsilon }$.

### Proof of Theorem 1.2

We consider the *n*-fold symmetric temperature

$$\theta =\bar{\Theta }+\Theta ^{\text {lin}}+\Theta ^{\text {cor}} \in C((-\infty ,\bar{\tau }],H^m\cap \dot{H}^{\frac{\alpha -2}{2}}),$$

as obtained from Theorems 3.3 and 3.5. Here we have b=0, and thus f=0. It follows that θ satisfies the conditions in Theorem 1.2. By shifting the time interval, we can assume that $\bar{\tau }=0$.

### The Golovkin Trick

In a recent preprint [61], Dolce and Mescolini revived a clever trick from Golovkin [82] which allows proving non-uniqueness once self-similar instability is established at the linear level. As the authors point out, the trick appears to work for quadratic PDEs. Here, we revisit this trick in the context of the α-SQG equation for the reader’s convenience.

Once Steps (1)-(3) have been completed, we have a self-similarly unstable vortex $\bar{\Theta }\in C_c^\infty$ and the corresponding eigenvalue $\lambda \in \mathbb {C}_+$ and eigenfunction $W\in C_c^m$ solving $L_b W=\lambda W$, with which we define $\Theta ^{\text {lin}}=\Re (e^{\lambda \tau }W)$. Then, we consider the two temperatures

3.15 $$\begin{aligned} \Theta _\pm =\bar{\Theta }\pm \Theta ^{\text {lin}}. \end{aligned}$$

It is straightforward to check that $\Theta _+$ and $\Theta _-$ solve the (self-similar) α-SQG equation (2.3) with the same initial condition

$$ \Theta _{\pm }|_{\tau =-\infty }=\bar{\Theta }, $$

and the Golovkin force

$$ G=F+V^{\text {lin}}\cdot \nabla \Theta ^{\text {lin}}, $$

where *F* is the Vishik force (3.5). The smoothness of the solutions allows us to conclude Theorem 3.1 through the Sobolev scaling (Proposition 2.2). Vishik’s and Golovkin’s forces differ in several aspects. On the one hand, *F* is radially symmetric and self-similar, while *G* is *n*-fold symmetric and depends on τ due to the quadratic term. On the other hand, F=0 for b=0, while *G* is not.

## Piecewise Constant Unstable Vortex

In this section we construct a piecewise constant unstable vortex. To this end, we need to solve the linear stability equation (3.7)

4.1 $$\begin{aligned} \frac{1}{r} \int _0^\infty \left( I_{n,\alpha }\left( \frac{r}{s}\right) h(s){-}I_{1,\alpha }\left( \frac{r}{s}\right) h(r)\right) \partial _r\bar{\theta }(s)s^{1-\alpha }\,\textrm{d}s =zh(r) \quad r{\in }\text {supp}(\partial _r\bar{\theta }),\nonumber \\ \end{aligned}$$

for some profile *h* and eigenvalue *z* with ℑz>0, to be determined.

### The kernel $I_{n,\alpha }$

In this section we analyze the parametric integral

4.2 $$\begin{aligned} I_{n,\alpha }(\sigma ) =\frac{1}{\alpha }\int _{-\pi }^{\pi }\frac{\cos (n\beta )}{|\sigma -e^{i\beta }|^{\alpha }}\,\textrm{d}\beta =\frac{\sigma }{n}\int _{-\pi }^{\pi }\frac{\sin (\beta )\sin (n\beta )}{|\sigma -e^{i\beta }|^{2+\alpha }}\,\textrm{d}\beta ; \end{aligned}$$

see Remark 2.4 for the last equality. The function (4.2) is well defined and smooth for all $\sigma \ge 0$ with $\sigma \ne 1$. Notice that $I_{n,\alpha }(\sigma )\rightarrow 0$ as $\sigma \rightarrow 0,\infty$. However, the analysis of the behavior near σ=1 is rather cumbersome and takes a few technical lemmas. For our purposes, it will be enough to focus on the range

$$ |1-\sigma |\le \frac{1}{2}. $$

The proofs of the following lemmas will be carried out for α>0. However, in the statements, we will also include the case α=0 since the formulas extend to this case. In fact, the kernel $I_{n,0}$ can be computed explicitly (see [31, Appendix])

4.3 $$\begin{aligned} I_{n,0}(\sigma ) =\frac{\pi }{n}\min \{\sigma ,\sigma ^{-1}\}^n. \end{aligned}$$

In the first lemma, we compute the limit of $I_{n,\alpha }(\sigma )$ as $\sigma \rightarrow 1$ explicitly. The proof is simpler for n=2, but we include the general case to address any *n*-fold symmetry in Theorem 1.2. Continuity follows immediately from the dominated convergence theorem, but computing $I_{n,\alpha }(1)$ is more involved and requires the use of certain combinatorial identities, as well as properties of the Beta and Gamma functions.

#### Lemma 4.1

Let $n\in \mathbb {N}$. For $0\le \alpha <1$, the function $I_{n,\alpha }(\sigma )$ is continuous at σ=1. Moreover,

$$ I_{n,\alpha }(1) =\frac{D_\alpha }{1-\alpha }F_n(\alpha )>0, $$

where

$$ D_\alpha =\frac{2\sqrt{\pi }}{2^\alpha } \frac{\Gamma \left( \frac{3-\alpha }{2}\right) }{\Gamma \left( 2-\frac{\alpha }{2}\right) }, $$

$$F_1=1$$ and, for n≥2,

$$ F_n(\alpha ) =\prod _{k=1}^{n-1}f_k(\alpha ) \quad \text {with}\quad f_k(\alpha ) =\frac{2k+\alpha }{2k+(2-\alpha )}. $$

For $1\le \alpha <2$, it holds that

$$ \lim _{\sigma \rightarrow 1}I_{n,\alpha }(\sigma )=\infty . $$

#### Proof

For 0<α<1, the integrand inside $I_{n,\alpha }(\sigma )$ is integrable at σ=1, namely, by elementary trigonometric identities,

4.4 $$\begin{aligned} I_{n,\alpha }(1)= &   \frac{2}{\alpha }\int _0^{\pi } \frac{\cos (n\beta )}{|1-e^{i\beta }|^\alpha }\,\textrm{d}\beta =\frac{2}{\alpha }\int _0^{\pi }\frac{\cos (n\beta )}{(2\sin (\beta /2))^\alpha }\,\textrm{d}\beta \nonumber \\= &   \frac{2^{2-\alpha }}{\alpha } \int _0^{\pi /2}\frac{\cos (2n\beta )}{(\sin \beta )^\alpha }\,\textrm{d}\beta . \end{aligned}$$

Then, we apply that $\cos (2n\beta )=T_{2n}(\cos \beta )$, where

$$ T_{2n}(x) =\sum _{j=0}^n\left( {\begin{array}{c}2n\\ 2j\end{array}}\right) (x^2-1)^jx^{2(n-j)}, $$

is the Chebyshev polynomial of degree 2*n*. Hence, by making the changes of variables $u=\sin \beta$ and $v=u^2$, we get

4.5 $$\begin{aligned} \begin{aligned} (4.4)&=\frac{2^{2-\alpha }}{\alpha } \sum _{j=0}^n\left( {\begin{array}{c}2n\\ 2j\end{array}}\right) (-1)^j \int _0^1\frac{u^{2j}(1-u^2)^{n-j}}{u^\alpha }\frac{\,\textrm{d}u}{\sqrt{1-u^2}}\\&=\frac{2^{2-\alpha }}{\alpha } \sum _{j=0}^n\left( {\begin{array}{c}2n\\ 2j\end{array}}\right) (-1)^j \int _0^1 v^{j-\frac{\alpha }{2}}(1-v)^{n-j-\frac{1}{2}}\frac{\,\textrm{d}v}{2\sqrt{v}}\\&=\frac{2^{1-\alpha }}{\alpha } \sum _{j=0}^n\left( {\begin{array}{c}2n\\ 2j\end{array}}\right) (-1)^j B\left( j+\frac{1-\alpha }{2},n-j+\frac{1}{2}\right) , \end{aligned} \end{aligned}$$

where *B* is the Beta function. By applying the identity

$$ B(x+j,y-j) =B(x,y)\prod _{k=1}^j \frac{x+k-1}{y-k}, $$

we get,

4.6 $$\begin{aligned} (4.5) =\frac{2^{1-\alpha }}{\alpha } B\left( \frac{1-\alpha }{2},n+\frac{1}{2}\right) p_n(\alpha ), \end{aligned}$$

where $p_n$ is the following polynomial of degree *n*

$$\begin{aligned} p_n(\alpha )&=\sum _{j=0}^n\left( {\begin{array}{c}2n\\ 2j\end{array}}\right) (-1)^j \prod _{k=1}^j \frac{\frac{1-\alpha }{2}+k-1}{n+\frac{1}{2}-k}\\&=\sum _{j=0}^n\left( {\begin{array}{c}2n\\ 2j\end{array}}\right) (-1)^j \prod _{k=1}^j \frac{2k-1-\alpha }{2(n-k)+1}\\&=\sum _{j=0}^n\left( {\begin{array}{c}n\\ j\end{array}}\right) (-1)^j \prod _{k=1}^j \frac{2k-1-\alpha }{2k-1}. \end{aligned}$$

In the last equality, we have applied that

$$ \left( {\begin{array}{c}2n\\ 2j\end{array}}\right) =\prod _{i=0}^{2j-1}\frac{2n-i}{i+1} =\prod _{k=0}^{j-1} \frac{2(n-k)}{2k+1} \frac{2(n-k)-1}{2(k+1)} =\left( {\begin{array}{c}n\\ j\end{array}}\right) \prod _{k=1}^{j} \frac{2(n-k)+1}{2k-1}, $$

where we have separated the cases i=2k and i=2k+1 for k=0,…,j-1 in the second equality.

Next, we claim that we can express $p_n$ simply as

4.7 $$\begin{aligned} p_n(\alpha )=c_n q_n(\alpha ) \quad \text {with}\quad q_n(\alpha )=\prod _{m=0}^{n-1}(2m+\alpha ), \end{aligned}$$

where $c_n$ is a constant, to be determined. Since both $p_n$ and $q_n$ are polynomials of degree *n*, it is sufficient to check that the roots of $p_n$ are the same as $q_n$, namely $\alpha _m=-2m$ for m=0,…,n-1. Notice that we can write that

4.8 $$\begin{aligned} p_n(-2m) =\sum _{j=0}^n\left( {\begin{array}{c}n\\ j\end{array}}\right) (-1)^j \pi _m(j), \end{aligned}$$

where $\pi _0=1$ and, for any m=1,…,n,

$$ \pi _m(j) = \prod _{k=1}^j \frac{2k-1+2m}{2k-1} =\prod _{k=1}^m \frac{2k-1+2j}{2k-1} $$

is a polynomial of degree *m*. The last equality is trivial for j=m. The case j>m follows by checking that some numerators and denominators cancel out. The case j<m is symmetric.

Since the first (n-1) derivatives of the polynomial

$$ (1+x)^n=\sum _{j=0}^n\left( {\begin{array}{c}n\\ j\end{array}}\right) x^j $$

vanish at x=-1, it follows that

$$ \sum _{j=0}^n\left( {\begin{array}{c}n\\ j\end{array}}\right) (-1)^j \pi (j)=0, $$

for any polynomial π of degree strictly less that *n*. In particular, (4.8)=0 for any m=0,…,n-1. For m=n, since we can decompose

$$ \pi _{n} =\tilde{\pi }_n +\pi \quad \text {with}\quad \tilde{\pi }_n(j)=2^n \prod _{k=0}^{n-1}\frac{j-k}{2k+1}, $$

where π is a polynomial of degree strictly less that *n*, we deduce that

$$ p_n(-2n) =\sum _{j=0}^n\left( {\begin{array}{c}n\\ j\end{array}}\right) (-1)^j\tilde{\pi }_n(j) =(-2)^n\tilde{\pi }_n(n) =(-2)^n n!\prod _{k=0}^{n-1}\frac{1}{2k+1}. $$

Thus, since

$$ q_n(-2n) =\prod _{k=0}^{n-1}(2k-2n) =(-2)^nn!, $$

we determine the constant in (4.7)

$$ c_n=\frac{p_n}{q_n}(-2n) =\prod _{k=0}^{n-1}\frac{1}{2k+1}. $$

Therefore, we have

4.9 $$\begin{aligned} (4.6) =\frac{2^{1-\alpha }}{\alpha } B\left( \frac{1-\alpha }{2},n+\frac{1}{2}\right) \prod _{k=0}^{n-1}\frac{2k+\alpha }{2k+1}. \end{aligned}$$

Next, by applying the identities

$$ B(x,y) =\frac{\Gamma (x)\Gamma (y)}{\Gamma (x+y)}, \quad \quad \Gamma (n+x) =\Gamma (x)\prod _{k=0}^{n-1}(k+x), $$

we get

$$ B\left( \frac{1-\alpha }{2},n+\frac{1}{2}\right) =\frac{\Gamma \left( \frac{1-\alpha }{2}\right) \Gamma \left( n+\frac{1}{2}\right) }{\Gamma \left( n+1-\frac{\alpha }{2}\right) } =\frac{\Gamma \left( \frac{1-\alpha }{2}\right) \Gamma \left( \frac{1}{2}\right) }{\Gamma \left( 1-\frac{\alpha }{2}\right) } \prod _{k=0}^{n-1}\frac{2k+1}{2k+(2-\alpha )}. $$

This implies that

4.10 $$\begin{aligned} (4.9) =\frac{2^{1-\alpha }\sqrt{\pi }}{\alpha } \frac{\Gamma \left( \frac{1-\alpha }{2}\right) }{\Gamma \left( 1-\frac{\alpha }{2}\right) } \prod _{k=0}^{n-1}\frac{2k+\alpha }{2k+(2-\alpha )}. \end{aligned}$$

For $1\le \alpha <2$, by expanding in Taylor series

$$ I_{n,\alpha }(\sigma ) =\frac{1}{\alpha }\int _{-\pi }^{\pi } \frac{1}{|\sigma -e^{i\beta }|^\alpha }\,\textrm{d}\beta +\frac{1}{\alpha }\int _{-\pi }^{\pi } \frac{O(\beta ^2)}{|\sigma -e^{i\beta }|^\alpha }\,\textrm{d}\beta , $$

it is clear that the second term is finite, while the first one diverges to infinity as $\sigma \rightarrow 1$. □

In the next two lemmas we study the behavior of $I_{n,\alpha }(\sigma )$ near σ=1 for $0\le \alpha <1$ and α=1, respectively.

#### Lemma 4.2

Let $n\in \mathbb {N}$ and $0\le \alpha <1$. There exists C>0 such that

$$ |I_{n,\alpha }'(\sigma )| \le \frac{C}{|1-\sigma |^\alpha }, $$

for all $0<|\sigma -1|\le \frac{1}{2}$. Therefore,

$$ |I_{n,\alpha }(\sigma )-I_{n,\alpha }(1)| \le C\frac{|1-\sigma |^{1-\alpha }}{1-\alpha }. $$

#### Proof

For 0<α<1 and $0<|\sigma -1|\le \frac{1}{2}$, we can differentiate under the integral sign

$$ I_{n,\alpha }'(\sigma ) =-\int _{-\pi }^{\pi }\frac{\sigma -\cos \beta }{|\sigma -e^{i\beta }|^{2+\alpha }}\cos (n\beta )\,\textrm{d}\beta . $$

This can be bounded by

$$\begin{aligned} |I_{n,\alpha }' (\sigma )|&\le 2 \int _{0}^{\pi }\frac{1}{|\sigma -e^{i\beta }|^{1+\alpha }}\,\textrm{d}\beta = 4\int _{0}^{\pi /2} \frac{1}{\left( (1-\sigma )^2+\sigma (2\sin \beta )^2\right) ^{\frac{1+\alpha }{2}}}\,\textrm{d}\beta \\&= 4\int _{0}^{\pi /2} \frac{1-\cos \beta }{\left( (1-\sigma )^2+\sigma (2\sin \beta )^2\right) ^{\frac{1+\alpha }{2}}}\,\textrm{d}\beta \\&\quad +4\int _{0}^{\pi /2} \frac{\cos \beta }{\left( (1-\sigma )^2+\sigma (2\sin \beta )^2\right) ^{\frac{1+\alpha }{2}}}\,\textrm{d}\beta =A+B. \end{aligned}$$

The first term *A* can be bounded easily. For the bad term *B*, by making the changes of variables $u=\sin \beta$ and $w=\frac{2\sqrt{\sigma }}{|1-\sigma |}u$, we estimate

$$\begin{aligned} B&=4\int _{0}^{1} \frac{\,\textrm{d}u}{\left( (1-\sigma )^2+(2\sigma u)^2\right) ^{\frac{1+\alpha }{2}}} =\frac{2}{\sigma |1-\sigma |^\alpha }\int _{0}^{\frac{2\sqrt{\sigma }}{|1-\sigma |}} \frac{\,\textrm{d}w}{\left( 1+w^2\right) ^{\frac{1+\alpha }{2}}}\\&\le \frac{4}{|1-\sigma |^\alpha }\int _{0}^{\infty } \frac{\,\textrm{d}w}{\left( 1+w^2\right) ^{\frac{1+\alpha }{2}}} \le \frac{C}{\alpha |1-\sigma |^\alpha }. \end{aligned}$$

Notice that for α=0 the function $I_{n,0}(\sigma )$ is Lipschitz at σ=1 (see (4.3)). Finally, we apply the fundamental theorem of calculus. □

The next lemma was proven in [27, Lemma 4.12]. Here, we present a more direct proof for the reader’s convenience.

#### Lemma 4.3

Let $n\in \mathbb {N}$. There exists $R\in C^1$ such that

$$ I_{n,1}(\sigma )=-\frac{2}{\sqrt{\sigma }}\log |1-\sigma |+R(\sigma ) $$

for all $$0<|\sigma -1|\le \frac{1}{2}$$.

#### Proof

We split

$$\begin{aligned} I_{n,1}(\sigma )&=4\int _0^{\pi /2} \frac{\cos (2n\beta )}{\sqrt{(1-\sigma )^2+\sigma (2\sin \beta )^2}}\,\textrm{d}\beta \\&=4\int _0^{\pi /2} \frac{\cos (2n\beta )-\cos \beta }{\sqrt{(1-\sigma )^2+\sigma (2\sin \beta )^2}}\,\textrm{d}\beta \\&\quad +4\int _0^{\pi /2} \frac{\cos \beta }{\sqrt{(1-\sigma )^2+\sigma (2\sin \beta )^2}}\,\textrm{d}\beta =A+B. \end{aligned}$$

By the dominated convergence theorem, it is easy to check that $A\in C^1$. In fact, $A=-J_{2n,1}$ which is analyzed in more detail in Lemmas 4.4 and 4.5. For the bad term *B*, by making the changes of variables $u=\sin \beta$ and $w=\frac{2\sqrt{\sigma }}{|1-\sigma |}u$, we compute that

$$\begin{aligned} B=\frac{2}{\sqrt{\sigma }}\int _0^{\frac{2\sqrt{\sigma }}{|1-\sigma |}} \frac{\,\textrm{d}w}{\sqrt{1+w^2}} =\frac{2}{\sqrt{\sigma }}\textrm{arcsinh}\left( \frac{2\sqrt{\sigma }}{|1-\sigma |}\right) =-\frac{2}{\sqrt{\sigma }}\log |1-\sigma |+C, \end{aligned}$$

where *C* is a smooth function. In the last equality we have used the formula

$$\begin{aligned} \textrm{arcsinh}(x)=\log (x+\sqrt{x^2+1}), \end{aligned}$$

which implies that

$$ C=\frac{2}{\sqrt{\sigma }} \log (2\sqrt{\sigma } +\sqrt{4\sigma +(1-\sigma )^2}). $$

Finally, we take R=A+C. □

The rest of this section will be devoted to analyzing the operator

4.11 $$\begin{aligned} J_{n,\alpha }(\sigma ) =(I_{1,\alpha }-I_{n,\alpha })(\sigma ) =\frac{1}{\alpha }\int _{-\pi }^{\pi }\frac{\cos (\beta )-\cos (n\beta )}{|\sigma -e^{i\beta }|^{\alpha }}\,\textrm{d}\beta , \end{aligned}$$

which is now well defined at σ=1 for α<3 thanks to the extra cancellation in the numerator.

#### Lemma 4.4

Let n≥2. For $0\le \alpha <3$, the function $J_{n,\alpha }(\sigma )$ is continuous at σ=1. Moreover,

$$ J_{n,\alpha }(1)>0. $$

For $3\le \alpha <5$, it holds that

$$ \lim _{\sigma \rightarrow 1}J_{n,\alpha }(\sigma )=\infty . $$

#### Proof

For α<3, the integrand inside $J_{n,\alpha }$ is integrable at σ=1. As we saw in Lemma 4.1, we can compute that

$$ J_{n,\alpha }(1) =D_\alpha \frac{1-F_n(\alpha )}{1-\alpha }, $$

which is indeed valid in the full range $0\le \alpha <3$. On the one hand, $D_\alpha >0$. On the other hand, for 0<α<1, we have $f_k(\alpha )<1$ for all k=0,…,n-1, and thus

$$ F_n(\alpha )<1, $$

while for 1<α<3, we have $f_k(\alpha )>1$ for all k=0,…,n-1, and thus

$$ F_n(\alpha )>1. $$

For α=1, we have $f_k(1)=1$ for all k=0,…,n-1, and thus

$$ F_n(1)=1. $$

By applying the product rule, we deduce that

$$ F_n'(1) =\sum _{k=1}^{n-1}f_k'(1) =\sum _{k=1}^{n-1}\frac{4k+2}{(2k+2)^2}>0. $$

Hence,

$$ J_{n,1}(1) =D_1 F_n'(1)>0. $$

For $3\le \alpha <5$, by expanding the cosine functions in the integrands in Taylor series,

$$ J_{n,\alpha }(\sigma ) =\frac{n^2-1}{2\alpha }\int _{-\pi }^{\pi } \frac{\beta ^2}{|\sigma -e^{i\beta }|^\alpha }\,\textrm{d}\beta +\frac{1}{\alpha }\int _{-\pi }^{\pi } \frac{O(\beta ^4)}{|\sigma -e^{i\beta }|^\alpha }\,\textrm{d}\beta , $$

it is clear that the second term is finite, while the first one diverges to infinity as $\sigma \rightarrow 1$. □

#### Lemma 4.5

Let $n\in \mathbb {N}$. For $0\le \alpha <2$, the function $J_{n,\alpha }(\sigma )$ is differentiable at σ=1. Moreover,

$$ J_{n,\alpha }'(1) =-\frac{\alpha }{2}J_{n,\alpha }(1). $$

For $0\le \alpha <1$, the function $J_{n,\alpha }(\sigma )$ is twice differentiable at σ=1. Moreover,

$$ J_{n,\alpha }''(1) =-\alpha J_{n,2+\alpha }(1) +\frac{\alpha (2+\alpha )}{4}J_{n,\alpha }(1). $$

For $1\le \alpha <2$, it holds that

$$ \lim _{\sigma \rightarrow 1}J_{n,\alpha }''(\sigma )=-\infty . $$

#### Proof

For any $0<|\sigma -1|\le \frac{1}{2}$, we can differentiate under the integral sign

$$ J_{n,\alpha }'(\sigma ) =-\int _{-\pi }^{\pi }\frac{\sigma -\cos \beta }{|\sigma -e^{i\beta }|^{2+\alpha }}(\cos \beta -\cos (n\beta ))\,\textrm{d}\beta . $$

The integrand can be bounded by

$$ \left| \frac{\sigma -\cos \beta }{|\sigma -e^{i\beta }|^{2+\alpha }}(\cos \beta -\cos (n\beta ))\right| \lesssim |\sigma -e^{i\beta }|^{1-\alpha } \lesssim \sin (\beta /2)^{1-\alpha }, $$

which is integrable for any α<2. Thus, we can apply the dominated convergence theorem in the limit $\sigma \rightarrow 1$ to get

$$ J_{n,\alpha }'(1) =-\int _{-\pi }^{\pi }\frac{1-\cos \beta }{|1-e^{i\beta }|^{2+\alpha }}(\cos \beta -\cos (n\beta ))\,\textrm{d}\beta =-\frac{\alpha }{2}J_{n,\alpha }(1), $$

where in the last equality we have applied the identity $(1-\cos \beta )=\frac{1}{2}|1-e^{i\beta }|^2.$ By differentiating under the integral sign again, we get

4.12 $$\begin{aligned} J_{n,\alpha }''(\sigma ) =-\alpha J_{n,2+\alpha }(\sigma ) +(2+\alpha )\int _{-\pi }^{\pi }\frac{(\sigma -\cos \beta )^2}{|\sigma -e^{i\beta }|^{2+\alpha }}(\cos \beta -\cos (n\beta ))\,\textrm{d}\beta .\nonumber \\ \end{aligned}$$

For α<1, by applying Lemma 4.4 and the dominated convergence theorem, we deduce that

$$ J_{n,\alpha }''(1) =-\alpha J_{n,2+\alpha }(1) +\frac{\alpha (2+\alpha )}{4}J_{n,\alpha }(1). $$

For $1\le \alpha <2$, the second integral in (4.12) is finite, while the first term $J_{n,2+\alpha }(\sigma )$ diverges to infinity as $\sigma \rightarrow 1$ by Lemma 4.4. □

### Ansatz

Given some parameters $0<r_1<r_2<\infty$ and c>0 to be determined, we consider the piecewise constant profile

4.13 $$\begin{aligned} \bar{\theta }(r) = \left\{ \begin{array}{rl} c, &  0<r\le r_1, \\ -1, &  r_1<r\le r_2, \\ 0, &  r>r_2, \end{array} \right. \end{aligned}$$

where we have chosen *c* satisfying

4.14 $$\begin{aligned} (1+c)r_1^2=r_2^2. \end{aligned}$$

In this case $\bar{\theta }$ has zero mean and

$$ \partial _r\bar{\theta } =-(1+c)\delta _{r_1} +\delta _{r_2}. $$

For the sake of simplicity we fix

$$ r_1=\sigma , \quad \quad r_2=1, $$

for some $\frac{1}{2}<\sigma <1$, to be determined. In the next section we will use again the letters $r_1$ and $r_2$ to make the notation more compact. In any case, (4.14) reads as

4.15 $$\begin{aligned} (1+c)\sigma ^2=1. \end{aligned}$$

Under our ansatz, it remains to solve the equation (4.1) at the points r=σ,1. This imposes two conditions on the vector h=(h(σ),h(1)), represented by the following linear system:

Ah=zh,

with

4.16 $$\begin{aligned} A= \left[ \begin{array}{cc} \sigma ^{-(2+\alpha )}J_{n,\alpha }(1)-\sigma ^{-1}I_{1,\alpha }(\sigma ) &  \sigma ^{-1}I_{n,\alpha }(\sigma ) \\ -\sigma ^{-1} I_{n,\alpha }(\sigma ) &  -J_{n,\alpha }(1) +\sigma ^{-1} I_{1,\alpha }(\sigma ) \end{array} \right] , \end{aligned}$$

where we have applied (4.15) and the identity

$$ I_{n,\alpha }(1/\sigma )=\sigma ^\alpha I_{n,\alpha }(\sigma ).$$

Recall that $I_{1,\alpha }(\sigma )$, $I_{n,\alpha }(\sigma )$ and $J_{n,\alpha }(1)$ are well defined for any α<2.

In order to find an eigenvector h≠0 we need to solve det(A-z)=0, which is a quadratic equation in *z*. Since *A* is real valued, one of its roots satisfies ℑz>0 if and only if its discriminant Δ is strictly negative. In the next lemma we compute Δ in terms of the parameter σ and the functions $I_{n,\alpha }(\sigma )$ and $J_{n,\alpha }(1)$.

#### Lemma 4.6

The discriminant Δ of the characteristic polynomial det(A-z)=0 satisfies that

$$ \sigma ^2\Delta (\sigma ) =((\sigma +\sigma ^{-(1+\alpha )})J_{n,\alpha }(1)-2I_{1,\alpha }(\sigma ))^2 -(2I_{n,\alpha }(\sigma ))^2. $$

#### Proof

We compute

$$ \det (A-z) =z^2-(\textrm{tr}A)z+\det A, $$

where

$$ \textrm{tr}A =(\sigma ^{-(2+\alpha )}-1)J_{n,\alpha }(1), $$

and

$$\begin{aligned} \det A&=(\sigma ^{-(2+\alpha )}J_{n,\alpha }(1){-}\sigma ^{-1}I_{1,\alpha }(\sigma )) (-J_{n,\alpha }(1) {+}\sigma ^{-1} I_{1,\alpha }(\sigma )) {+}\sigma ^{-2}I_{n,\alpha }(\sigma )^2\\&=-\sigma ^{-(2+\alpha )} J_{n,\alpha }(1)^2 +(\sigma ^{-(3+\alpha )}{+}\sigma ^{-1})I_{1,\alpha }(\sigma )J_{n,\alpha }(1) -\sigma ^{-2}I_{1,\alpha }(\sigma )^2\\&\quad +\sigma ^{-2}I_{n,\alpha }(\sigma )^2. \end{aligned}$$

Therefore,

$$\begin{aligned} \Delta&=(\textrm{tr}A)^2-4\det A\\&=(1+\sigma ^{-(2+\alpha )})^2J_{n,\alpha }(1)^2 -4((\sigma ^{-(3+\alpha )}+\sigma ^{-1})I_{1,\alpha }(\sigma )J_{n,\alpha }(1) \\  &\quad -\sigma ^{-2}(I_{1,\alpha }(\sigma )^2-I_{n,\alpha }(\sigma )^2))\\&=((1+\sigma ^{-(2+\alpha )})J_{n,\alpha }(1)-2\sigma ^{-1}I_{1,\alpha }(\sigma ))^2 -4\sigma ^{-2}I_{n,\alpha }(\sigma )^2, \end{aligned}$$

as we wanted to prove. □

Notice that Δ=0 for n=1. Thus, from now on we consider the case n≥2.

#### Proposition 4.1

For every n≥2 and $0\le \alpha <2$, there exists $\frac{1}{2}<\sigma <1$ such that $\Delta (\sigma )<0$. Therefore, there exist an eigenvalue $z\in \mathbb {C}$ with ℑz>0 and an eigenvector h≠0 solving Az=hz.

#### Proof

Fixed *n* and α, we know from Lemma 4.1 that there exist δ>0 and $\frac{1}{2}<\sigma _0<1$ such that

4.17 $$\begin{aligned} I_{n,\alpha }(\sigma )>\delta , \end{aligned}$$

for all $\sigma _0\le \sigma <1$. Thus, in these range of σ’s, it follows from Lemma 4.6, that $\Delta (\sigma )<0$ if and only if

$$ |(\sigma +\sigma ^{-(1+\alpha )})J_{n,\alpha }(1)-2I_{1,\alpha }(\sigma )|<2I_{n,\alpha }(\sigma ), $$

or, equivalently,

4.18 $$\begin{aligned} -4I_{n,\alpha }(\sigma )<L_{n,\alpha }(\sigma )<0, \end{aligned}$$

where we have abbreviated

$$ L_{n,\alpha }(\sigma ) =2J_{n,\alpha }(\sigma )-(\sigma +\sigma ^{-(1+\alpha )})J_{n,\alpha }(1). $$

By Lemma 4.4, we have

4.19 $$\begin{aligned} L_{n,\alpha }(1)=0. \end{aligned}$$

In light of (4.17) and (4.19), to conclude (4.18) it is enough to check that $L_{n,\alpha }$ is strictly increasing on an interval $\sigma _1\le \sigma <1$ for some $\sigma _1>\sigma _0$. We start by computing the first derivative

$$ L_{n,\alpha }'(\sigma ) =2J_{n,\alpha }'(\sigma )-(1-(1+\alpha )\sigma ^{-(2+\alpha )})J_{n,\alpha }(1). $$

By applying Lemma 4.5, we deduce that

$$ L_{n,\alpha }'(1) =2J_{n,\alpha }'(1)+\alpha J_{n,\alpha }(1) =0. $$

Next, we compute the second derivative:

$$ L_{n,\alpha }''(\sigma ) =2J_{n,\alpha }''(\sigma ) -(1+\alpha )(2+\alpha )\sigma ^{-(3+\alpha )}J_{n,\alpha }(1). $$

Finally, we apply Lemmas 4.4 and 4.5 again. For $0\le \alpha <1$, we have

$$ L_{n,\alpha }''(1) =2J_{n,\alpha }''(1) -(1+\alpha )(2+\alpha )J_{n,\alpha }(1) {=}-2\alpha J_{n,2+\alpha }(1) -\frac{(2+\alpha )^2}{2}J_{n,\alpha }(1){<}0. $$

For $1\le \alpha <2$, we have

$$ \lim _{\sigma \rightarrow 1}L_{n,\alpha }''(\sigma )=-\infty . $$

This concludes the proof. □

## Regularization

In this section we prove Theorem 3.3. To this end, we show that there is a regularization $\bar{\theta }^\varepsilon$, for some small ε>0, of the piecewise constant vortex $\bar{\theta }$ that we constructed in the previous section, that is also unstable. As we explained in Sect. 3, it is convenient to regularize $\bar{\theta }$ in such a way that $\bar{\theta }^\varepsilon$ also has zero mean. To this end, we take a mollifier $\eta \in C_c^\infty (I)$ with I=(-1,1) satisfying the properties of the following lemma:

### Lemma 5.1

There exists $\eta \in C_c^\infty (-1,1)$ such that

$$ \int _{-1}^{1}\eta (\rho )\,\textrm{d}\rho =1, \quad \quad \int _{-1}^{1}\eta (\rho )\rho \,\textrm{d}\rho =\int _{-1}^{1}\eta (\rho )\rho ^2\,\textrm{d}\rho =0. $$

### Proof

Let $$\chi \in C_c^\infty (0,1)$$ satisfying

$$ \int _0^1\chi \,\textrm{d}\rho =1. $$

We consider the even function $\eta \in C_c^\infty (-1,1)$ defined on (0, 1) by

$$ \eta (\rho )=\frac{1}{4}(3\chi (\rho )+\rho \partial _\rho \chi (\rho )). $$

Notice that η(0)=0. The mean of η equals

$$ \int _{-1}^{1}\eta \,\textrm{d}\rho =\frac{1}{2}\int _0^1(3\chi +\rho \partial _\rho \chi )\,\textrm{d}\rho =1. $$

Since η is even, we have

$$ \int _{-1}^1\eta \rho \,\textrm{d}\rho = 0. $$

The second momentum of η also vanishes

$$ \int _{-1}^{1}\eta \rho ^2\,\textrm{d}\rho = \frac{1}{2} \int _0^1\partial _\rho (\rho ^3\chi )\,\textrm{d}\rho =0. $$

This concludes the proof. □

Next, we use η to regularize $\bar{\theta }$. We rewrite (4.13) as

$$ \bar{\theta } =c-(1+c)1_{[r_1,\infty )} +1_{[r_2,\infty )}. $$

Let $0<\varepsilon <\frac{1}{3}\min \{r_1,r_2-r_1\}$, to be determined. This reformulation allows mollifying $\bar{\theta }$ without modifying its value at $r=0^+$, namely we define

5.1 $$\begin{aligned} \bar{\theta }^\varepsilon =c+(-(1+c)1_{[r_1,\infty )} +1_{[r_2,\infty )})*\eta ^\varepsilon , \end{aligned}$$

where $\eta ^\varepsilon$ is the usual approximation of the identity

$$ \eta ^\varepsilon (\rho ) =\frac{1}{\varepsilon }\eta \left( \frac{\rho }{\varepsilon }\right) . $$

By definition, $\bar{\theta }^\varepsilon$ is smooth and agrees with $\bar{\theta }$ outside $B_{\varepsilon }(\{r_1,r_2\}) =B_\varepsilon (r_1)\cup B_\varepsilon (r_2).$ Therefore, since

$$ \partial _r\bar{\theta }^\varepsilon =0 \quad \text {outside}\quad B_\varepsilon (\{r_1,r_2\}), $$

it remains to find an eigenvalue $z^\varepsilon \in \mathbb {C}$ with $\Im z^\varepsilon >0$ and a profile $h^\varepsilon$ satisfying the linear stability equation (4.1)

5.2 $$\begin{aligned}  &   \frac{1}{r} \int _0^\infty \left( I_{n,\alpha }\left( \frac{r}{s}\right) h^\varepsilon (s)-I_{1,\alpha }\left( \frac{r}{s}\right) h^\varepsilon (r)\right) \partial _r\bar{\theta }^\varepsilon (s)s^{1-\alpha }\,\textrm{d}s =z^\varepsilon h^\varepsilon (r), \nonumber \\  &   \quad r\in B_\varepsilon (\{r_1,r_2\}). \end{aligned}$$

### Rescaling

In this section we zoom into each interval $B_\varepsilon (r_j)$ through the change of variables

$$ r=r_j+\varepsilon \rho , $$

for $\rho \in I=(-1,1)$ and j=1,2. From now on we will denote that

$$ c_1=-(1+c), \quad \quad c_2=1, $$

order to make the notation more compact. In the next lemma, we use the conditions on the momenta of η.

#### Lemma 5.2

(Rescaling of $\bar{\theta }^\varepsilon$) It holds that

$$ \varepsilon \partial _r\bar{\theta }^\varepsilon (r) =c_j\eta (\rho ), $$

for $r=r_j+\varepsilon \rho$. Moreover, $\bar{\theta }^\varepsilon$ has zero mean.

#### Proof

By differentiating (5.1), we obtain the first claim,

$$ \varepsilon \partial _r\bar{\theta }^\varepsilon =\varepsilon (c_1\delta _{r_1} +c_2\delta _{r_2})*\eta ^\varepsilon =c_1 \eta \left( \frac{r-r_1}{\varepsilon }\right) +c_2 \eta \left( \frac{r-r_2}{\varepsilon }\right) . $$

Hence, by applying Lemma 5.1, we deduce that

$$\begin{aligned}  &   \int _0^\infty \bar{\theta }^\varepsilon r\,\textrm{d}r =-\frac{1}{2}\int _0^\infty \partial _r\bar{\theta }^\varepsilon r^2\,\textrm{d}r\\  &   =-\frac{1}{2} \sum _{j=1,2}c_j \int _{-1}^{1} \eta (\rho )(r_j+\varepsilon \rho )^2\,\textrm{d}\rho =-\frac{1}{2} \sum _{j=1,2}c_j r_j^2=0, \end{aligned}$$

where the last equality follows from (4.14). The second claim is also proved. □

We expand $h^\varepsilon$ in each interval $B_\varepsilon (r_j)$ as

$$ h^\varepsilon (r) =h_j+\varepsilon g_j(\rho ), $$

for $r=r_j+\varepsilon \rho$, and for some profiles $g_j\in L^2(I)$ to be determined. Here $h=(h_1,h_2)$ is the eigenvector we found in Proposition 4.1. Typically, we will deal with j=1,2 and will speak of $g=(g_1,g_2) \in L^2(I)^2$ to deal with both equations simultaneously. Similarly, we expand

$$ z^\varepsilon =z+\varepsilon y, $$

for some $y\in \mathbb {C}$, to be determined. Here *z* is the eigenvalue we found in Proposition 4.1.

For these $g\in L^2(I)^2$ and $y\in \mathbb {C}$, the linear stability equation (5.2) is rewritten as

5.3 $$\begin{aligned} A^\varepsilon (h+\varepsilon g) =(z+\varepsilon y)(h+\varepsilon g), \end{aligned}$$

where

$$\begin{aligned}  &   (A^\varepsilon f)_j(\rho ) =\sum _{k=1,2} c_k\int _{-1}^{1} \frac{(r_k+\varepsilon \varrho )^{1-\alpha }}{r_j+\varepsilon \rho }\\  &   \left( I_{n,\alpha }\left( \frac{r_j+\varepsilon \rho }{r_k+\varepsilon \varrho }\right) f_k(\varrho ) - I_{1,\alpha }\left( \frac{r_j+\varepsilon \rho }{r_k+\varepsilon \varrho }\right) f_j(\rho )\right) \eta (\varrho )\,\textrm{d}\varrho . \end{aligned}$$

Recall that we have used the change of variables $r=r_j+\varepsilon \rho$ and $s=r_k+\varepsilon \varrho$.

### Expansion of $A^\varepsilon$

We decompose

$$ A^\varepsilon =A^\varepsilon _0+A^\varepsilon _1, $$

into

5.4 $$\begin{aligned} \begin{aligned} (A_0^\varepsilon f)_j(\rho ) =&\, c_k\int _{-1}^{1} \frac{(r_k+\varepsilon \varrho )^{1-\alpha }}{r_j+\varepsilon \rho } \left( I_{n,\alpha }\left( \frac{r_j+\varepsilon \rho }{r_k+\varepsilon \varrho }\right) f_k(\varrho )\right. \\&\left. - I_{1,\alpha }\left( \frac{r_j+\varepsilon \rho }{r_k+\varepsilon \varrho }\right) f_j(\rho )\right) \eta (\varrho )\,\textrm{d}\varrho \\&-c_j\int _{-1}^{1} \frac{(r_j+\varepsilon \varrho )^{1-\alpha }}{r_j+\varepsilon \rho } J_{n,\alpha }\left( \frac{r_j+\varepsilon \rho }{r_j+\varepsilon \varrho }\right) f_j(\varrho ) \eta (\varrho )\,\textrm{d}\varrho \end{aligned} \end{aligned}$$

with k≠j, and

5.5 $$\begin{aligned} (A_1^\varepsilon f)_j(\rho ) =c_j\int _{-1}^{1} \frac{(r_j+\varepsilon \varrho )^{1-\alpha }}{r_j+\varepsilon \rho } I_{1,\alpha }\left( \frac{r_j+\varepsilon \rho }{r_j+\varepsilon \varrho }\right) (f_j(\varrho ) - f_j(\rho )) \eta (\varrho )\,\textrm{d}\varrho .\nonumber \\ \end{aligned}$$

Notice that

5.6 $$\begin{aligned} A_1^\varepsilon \mu =0, \end{aligned}$$

for any constant vector $\mu \in \mathbb {C}^2$. The operator $A_0^\varepsilon$ can be treated uniformly in α as done in the following lemma. As a corollary, we obtain a simplification of the linear stability equation.

#### Lemma 5.3

(Expansion of $A_0^\varepsilon$) Let $0\le \alpha \le 1$. It holds that

$$ A_0^\varepsilon =A_0+\varepsilon B_0, $$

where, for k≠j,

5.7 $$\begin{aligned} \begin{aligned} (A_0 f)_j(\rho ) =&\, c_k \frac{r_k^{1-\alpha }}{r_j}\int _{-1}^{1} \left( I_{n,\alpha }\left( \frac{r_j}{r_k}\right) f_k(\varrho ) - I_{1,\alpha }\left( \frac{r_j}{r_k}\right) f_j(\rho )\right) \eta (\varrho )\,\textrm{d}\varrho \\&-c_j \frac{r_j^{1-\alpha }}{r_j} J_{n,\alpha }(1)\int _{-1}^{1} f_j(\varrho ) \eta (\varrho )\,\textrm{d}\varrho , \end{aligned} \end{aligned}$$

and $B_0=B_0^\varepsilon :L^2(I)^2\rightarrow L^2(I)^2$ is uniformly bounded in ε. Moreover, for any $\mu \in \mathbb {C}^2$,

$$ A_0\mu =A\mu , $$

where *A* is the matrix in (4.16).

#### Proof

Recall that k≠j and that the kernels $I_{n,\alpha }(\sigma )$ are smooth away from σ=1, while $J_{n,\alpha }$ is $C^1$ by Lemma 4.5. Thus, all the functions in (5.4) multiplying *f* are $C^1$ in ε and the first statement follows. The second statement is a direct consequence of the definition of $A_0$ and that of *A* in (4.16), together with $\int \eta =1$. □

#### Corollary 5.1

For $0\le \alpha \le 1$, the linear stability equation (5.3) can be rewritten as

5.8 $$\begin{aligned} (A^\varepsilon -z)g =(y-B_0)h+ \varepsilon yg. \end{aligned}$$

#### Proof

Since $h\in \mathbb {C}^2$, by applying (5.6) and Lemma 5.3, we deduce that

$$ A^\varepsilon h =A_0^\varepsilon h =A_0h+\varepsilon B_0 h. $$

On the other hand,

$$ (z+\varepsilon y)(h+\varepsilon g) =zh+\varepsilon (zg+yh)+\varepsilon ^2 yg. $$

Finally, we apply that $A_0h=Ah=zh$, thus canceling the zero-order term. □

The operator $A_1^\varepsilon$ is however more delicate, and needs to be analyzed separately for α<1 or α=1.

### Case $0\le \alpha <1$

#### Lemma 5.4

(Expansion of $A_1^\varepsilon$) Let $0\le \alpha <1$. It holds that

$$ A_1^\varepsilon =A_1+\frac{\varepsilon ^{1-\alpha }}{1-\alpha } B_1, $$

where

$$ (A_1 f)_j(\rho ) =\frac{c_j}{r_j^\alpha } I_{1,\alpha }(1) \left( \int _{-1}^{1} f_j(\varrho )\eta (\varrho )\,\textrm{d}\varrho - f_j(\rho ) \right) , $$

and $B_1=B_1^\varepsilon :L^2(I)^2\rightarrow L^2(I)^2$ is uniformly bounded in ε.

#### Proof

It follows by expanding around ε=0 the functions in (5.5) multiplying *f*. Firstly, notice that the factor

$$ \frac{(r_j+\varepsilon \varrho )^{1-\alpha }}{r_j+\varepsilon \rho } $$

is smooth in ε. Secondly, for the kernel $I_{1,\alpha }$, we apply Lemma 4.2 for

$$\sigma =\frac{r_j+\varepsilon \rho }{r_j+\varepsilon \varrho } \quad \Rightarrow \quad 1-\sigma =\frac{\varepsilon (\varrho -\rho )}{r_j+\varepsilon \varrho }. $$

More precisely, we have

$$ I_{1,\alpha }\left( \frac{r_j+\varepsilon \rho }{r_j+\varepsilon \varrho }\right) =I_{1,\alpha }(1)+O\left( \frac{(\varepsilon |\rho -\varrho |)^{1-\alpha }}{1-\alpha }\right) , $$

which implies that $B_1$ is bounded. □

#### Corollary 5.2

For $0\le \alpha <1$, the linear stability equation (5.8) can be rewritten as

5.9 $$\begin{aligned} (A-z)g =(y-B_0)h+ \left( \varepsilon y-\frac{\varepsilon ^{1-\alpha }}{1-\alpha }B\right) g, \end{aligned}$$

where

$$ A=A_0+A_1, \quad \quad B=(1-\alpha )\varepsilon ^\alpha B_0+B_1. $$

#### Proof

It follows by applying Lemmas 5.3 and 5.4. □

#### Inverting the Linear Operator

Notice that we can split

$$\begin{aligned} A=A_0+A_1=D+C, \end{aligned}$$

where

$$\begin{aligned} (Df)_j(\rho )= &   -f_j(\rho )\sum _{k=1,2} c_k\frac{r_k^{1-\alpha }}{r_j} I_{1,\alpha }\left( \frac{r_j}{r_k}\right) ,\\ (Cf)_j= &   \sum _{k=1,2} c_k\frac{r_k^{1-\alpha }}{r_j} I_{n,\alpha }\left( \frac{r_j}{r_k}\right) \int _{-1}^{1}f_k(\varrho )\eta (\varrho )\,\textrm{d}\varrho . \end{aligned}$$

Notice that *D* is a diagonal operator, and therefore (D-z) is invertible, and that *C* is constant valued, that is, $C:L^2(I)^2\rightarrow \mathbb {C}^2$. The next lemma takes advantage of this structure to reformulate the linear stability equation in order to find the corresponding eigenvalue with positive imaginary part. The proof is analogous to [31, Lemma 4.4]. Since it is brief, we recall it here for the sake of completeness.

##### Lemma 5.5

For $0\le \alpha <1$, the linear stability equation (5.9) is equivalent to

5.10 $$\begin{aligned} \begin{aligned} g&=f +\gamma h^* + \delta h,\\ y&=\frac{\langle Cf,h^{*\perp }\rangle }{\langle h,h^{*\perp }\rangle }, \end{aligned} \end{aligned}$$

where f=f(g,y)

5.11 $$\begin{aligned} f=(D-z)^{-1}\left( -B_0 h+\left( \varepsilon y-\frac{\varepsilon ^{1-\alpha }}{1-\alpha }B\right) g\right) , \end{aligned}$$

with $$\gamma =\gamma (f)$$

$$ \gamma =-\frac{1}{2iz_2|h|^2}\left( \frac{\langle h,h^*\rangle }{\langle h,h^{*\perp }\rangle }\langle Cf,h^{*\perp }\rangle -\langle Cf,h^*\rangle \right) , $$

and $\delta \in \mathbb {C}$ is arbitrary.

##### Proof

Let *f* be given as in (5.11). We write the unknown *g* as

g=f+μ,

in terms of some μ, to be determined. Then, *g* solves (5.9) if and only if μ solves

5.12 $$\begin{aligned} (A-z)\mu =yh-Cf. \end{aligned}$$

Since A=D+C with *D* diagonal and *C* constant valued, we deduce that μ must be a constant vector. Therefore, *A* is acting on constant vectors in (5.12), and it is thus given by (4.16). Since $\textrm{Ker}(A-z)=\textrm{span}(h)$, $\textrm{Im}(A-z)=\textrm{span}(h^*)$, and $\{h,h^*\}$ is a basis of $\mathbb {C}^2$, we take

$$ \mu =\gamma h^*+\delta h, $$

in terms of some $\gamma ,\delta \in \mathbb {C}$, to be determined. Notice that

5.13 $$\begin{aligned} (A-z)\mu =\gamma (A-z)h^* =\gamma (A-(z^*+2iz_2))h^* =-2i z_2\gamma h^*. \end{aligned}$$

Hence, multiplying (5.12) by $h^{*\perp }=(-h_2^*,h_1^*)$, we get the compatibility condition for *y*

$$ 0 =y\langle h,h^{*\perp }\rangle -\langle Cf,h^{*\perp }\rangle . $$

Notice that $\langle h,h^{*\perp }\rangle \ne 0$ since $\{h,h^*\}$ is a basis of $\mathbb {C}^2$. On the other hand, multiplying (5.12) by $h^*$, and using (5.13), we can determine γ from the equality

$$ -2iz_2\gamma |h|^2 =y\langle h,h^*\rangle -\langle Cf,h^*\rangle . $$

The proof of the lemma is concluded. □

Since δ is arbitrary, we will take δ=0 for simplicity.

#### Fixed Point Argument

For ε=0 the equation (5.10) is explicit for (*g*, *y*) and thus we can find a solution $(g^0,y^0)$. For small ε>0 we will apply a fixed point argument. The proof is again analogous to [31, Proposition 4.5].

##### Proposition 5.1

Let $0\le \alpha <1$. For every $M>\Vert (g^0,y^0)\Vert _{L^2(I^2)\times \mathbb {C}}$ there exists $\varepsilon _0>0$ satisfying that: for every $0\le \varepsilon \le \varepsilon _0$ there exists $(g^\varepsilon ,y^\varepsilon )\in L^2(I)^2\times \mathbb {C}$ solving (5.10) with $\Vert (g^\varepsilon ,y^\varepsilon )\Vert _{L^2(I)^2\times \mathbb {C}}\le M$. In particular, $\Im z^\varepsilon >0$ for $\varepsilon <\frac{\Im z}{M}$.

##### Proof

We fix $0\le \alpha <1$. Let us denote by *F* the map on the right-hand side of (5.10), that is, we rewrite this equation compactly as

(g,y)=F(ε,g,y).

For ε=0, the functional *F* is constant, namely, $F(0,g,y)=(g^0,y^0)$. Since all the operators involved in *F* are bounded, depend continuously on ε at zero, and $\Vert (g^0,y^0)\Vert _{L^2(I)^2\times \mathbb {C}}<M$, there exists $\varepsilon _0>0$ such that *F* maps the ball $B_M$ of $L^2(I)^2\times \mathbb {C}$ into itself. Moreover, we can choose $\varepsilon _0>0$ such that *F* becomes a contraction on $B_M$. Then, we can apply the classical Banach fixed point theorem to find our required solution. □

### Case α=1

#### Lemma 5.6

(Expansion of $A_1^\varepsilon$) For α=1,

$$ A_1^\varepsilon =(\log \varepsilon ) A_1+ B_1, $$

where

$$ (A_1f)_j(\rho )=\frac{2c_j}{r_j}\left( f_j(\rho )-\int _{-1}^{1}f_j(\varrho )\eta (\varrho )\,\textrm{d}\rho \right) , $$

and $B_1=B_1^\varepsilon :L^2(I)^2\rightarrow L^2(I)^2$ is uniformly bounded in ε.

#### Proof

By Lemma 4.3, we have

$$ I_{1,1}\left( \frac{r_j+\varepsilon \rho }{r_j+\varepsilon \varrho }\right) =-2\log (\varepsilon |\rho -\varrho |)+O(1). $$

Since the convolution operator associated with $\log |\rho -\varrho |$ is bounded in $L^2$, the uniform bound for $B_1$ follows. □

Due to the $(\log \varepsilon )$ scaling, it is natural to decompose

5.14 $$\begin{aligned} g=\mu +\frac{f}{\log \varepsilon } \end{aligned}$$

for some $\mu \in \mathbb {C}^2$ and $f\in L^2(I)^2$, to be determined.

#### Corollary 5.3

For α=1, the linear stability equation (5.3) can be rewritten in terms of the ansatz (5.14) as

5.15 $$\begin{aligned} A_1f= &   (y-B_0)h+(z-A_0)\mu +\varepsilon (y-B_0)\mu +\frac{1}{\log \varepsilon }((z-A_0-B_1)\nonumber \\  &   +\varepsilon (y-B_0))f. \end{aligned}$$

From the definition of $A_1$, a necessary condition for solving (5.15) is that the right-hand side multiplied by η has zero mean.

#### Inverting the Linear Operator

Notice that we can split $$ A_1=D+C, $$ where

$$ (Df)_j(\rho )=\frac{2c_j}{r_j}f_j(\rho ), \quad \quad (Cf)_j=-\frac{2c_j}{r_j}\int _{-1}^{1}f_j\eta \,\textrm{d}\rho . $$

As before, *D* is an invertible diagonal operator, and *C* is constant value.

##### Lemma 5.7

For α=1, the linear stability equation (5.15) is equivalent to

5.16 $$\begin{aligned} \begin{aligned} f&=\bar{f}+\frac{D^{-1}}{\log \varepsilon }\left( ((z-A_0-B_1)+\varepsilon (y-B_0))f\right. \\&\left. -\int _{-1}^{1}((z-A_0-B_1)+\varepsilon (y-B_0))f\eta \,\textrm{d}\rho \right) ,\\ y&=\frac{\langle F,h^{*\perp }\rangle }{\langle h,h^{*\perp }\rangle },\\ \gamma&=\frac{\langle F-yh,h^*\rangle }{2iz_2|h|^2}, \end{aligned} \end{aligned}$$

where F=F(f,y,γ) is given by

$$ F=B_0h -\varepsilon (y-B_0)\mu -\frac{1}{\log \varepsilon }\int _{-1}^{1}((z-A_0-B_1)+\varepsilon (y-B_0))f\eta \,\textrm{d}\rho , $$

$\mu =\gamma h^*+\delta h$, and $\delta ,\bar{f}_j\in \mathbb {C}$ are arbitrary. Moreover, $\int f\eta =\bar{f}$.

##### Proof

We need to solve (5.15), that is,

$$ (y-B_0)h+(z-A_0)\mu +\varepsilon (y-B_0)\mu +\frac{1}{\log \varepsilon }\int _{-1}^{1}((z-A_0-B_1)+\varepsilon (y-B_0))f\eta \,\textrm{d}\rho =0. $$

Similarly to Lemma 5.5, we can write μ in the basis $\{h,h^*\}$ as

$$ \mu =\gamma h^* + \delta h, $$

and thus

$$ (A_0-z)\mu =-2iz_2\gamma h^*. $$

Therefore, we need to solve

$$\begin{aligned}  &   yh+2iz_2\gamma h^* =B_0h -\varepsilon (y-B_0)(\gamma h^* + \delta h)\\  &   \quad -\frac{1}{\log \varepsilon }\int _{-1}^{1}((z-A_0-B_1)+\varepsilon (y-B_0))f\eta \,\textrm{d}\rho =F. \end{aligned}$$

This equation determines γ and *y* as in the statement of the lemma. □

#### Fixed Point Argument

Similarly to Proposition 5.1, we can apply a fixed-point argument to the SQG for. The proof is analogous, and we therefore omit the details. We denote the solution to (5.16) for when ε=0 by $(f^0,y^0,\gamma ^0)$.

##### Proposition 5.2

For every $M>\Vert (f^0,y^0,\gamma ^0)\Vert _{L^2(I^2)\times \mathbb {C}}$ there exists $\varepsilon _0>0$ satisfying that: for every $0\le \varepsilon \le \varepsilon _0$ there exists $(f^\varepsilon ,y^\varepsilon ,\gamma ^\varepsilon )\in L^2(I)^2\times \mathbb {C}$ solving (5.16) with $\Vert (f^\varepsilon ,y^\varepsilon ,\gamma ^\varepsilon )\Vert _{L^2(I)^2\times \mathbb {C}}\le M$. In particular, $\Im z^\varepsilon >0$ for $\varepsilon <\frac{\Im z}{M}$.

Once $0<\varepsilon \le \varepsilon _0$ is fixed, we take the vortex

$$ \bar{\Theta }=\bar{\theta }^\varepsilon . $$

From now on the smooth vortex $\bar{\Theta }$ is fixed and thus we will omit ε for the sake of simplicity.

### Properties of the Vortex

We conclude this section by deriving some properties of the vortex $\bar{\Theta }$, the corresponding velocity field $\bar{V}(X) =\bar{V}_\phi (R)e_\phi$, and the eigenfunction $W\in U_n$, that will be useful in the next sections. The next proposition will be applied in the proof of Proposition 6.4.

#### Proposition 5.3

There exists a constant *C* and a smooth function *G* such that

$$ \frac{\bar{V}_\phi (R)}{R} = C+RG(R), $$

for $0<R\le \frac{r_1}{2}$. In particular,

$$\begin{aligned} \partial _R(C\log R+U(R))=\frac{\bar{V}_\phi (R)}{R^2}, \end{aligned}$$

where

$$ U(R)=\int _0^R G\,\textrm{d}S. $$

#### Proof

By Lemma 2.1, we have

5.17 $$\begin{aligned} \frac{\bar{V}_\phi (R)}{R} =-C_\alpha \int _0^\infty K_{1,\alpha }\left( \frac{R}{S}\right) \partial _R\bar{\Theta }(S)S^{-\alpha }\,\textrm{d}S, \end{aligned}$$

where we have applied Remark 2.4, namely,

$$ \frac{1}{\sigma }I_{1,\alpha }(\sigma ) =\int _{-\pi }^{\pi }\frac{(\sin \beta )^2}{|\sigma -e^{i\beta }|^{2+\alpha }}\,\textrm{d}\beta =K_{1,\alpha }(\sigma ). $$

Since $\partial _R\bar{\Theta }(S)=0$ outside $r_1-\varepsilon \le S\le r_2+\varepsilon$, we deduce that

$$ \lim _{R\rightarrow 0}\frac{\bar{V}_\phi (R)}{R} =-C_\alpha K_{1,\alpha }(0) \int _0^\infty \partial _R\bar{\Theta }(S)S^{-\alpha }\,\textrm{d}S=C, $$

where $K_{1,\alpha }(0)=\pi$. Thus, the fundamental theorem of calculus implies that

$$ \frac{\bar{V}_\phi (R)}{R} -C =-RC_\alpha \int _0^\infty \int _0^1 K_{1,\alpha }'\left( \lambda \frac{R}{S}\right) \,\textrm{d}\lambda \partial _R\bar{\Theta }(S)S^{-(1+\alpha )}\,\textrm{d}S =RG(R). $$

Finally, *G* is smooth since $K_{1,\alpha }(\sigma )$ is smooth away from σ=1. □

The next lemma will be applied in Sect. 7.7.

#### Lemma 5.8

For every $$i\in \mathbb {N}$$,

$$ R^i\partial ^i_R\left( \frac{\bar{V}_\phi }{R}\right) \in L^\infty . $$

#### Proof

By construction, $\bar{\Theta }\in C_c^\infty$ and has zero mean. In particular, $\bar{\Theta }\in H^m$, thus $\bar{V}\in H^{m+1-\alpha }$ for all $m\in \mathbb {N}$. Therefore, $\bar{V}\in C^\infty$. Hence, the statement holds in the annulus $\frac{1}{2}(r_1-\varepsilon )\le R\le \frac{3}{2}(r_2+\varepsilon )$. It remains to analyze the behavior near the origin and at infinity. By changing variables R=ST in (5.17) we deduce that

$$ \frac{\bar{V}_\phi (R)}{R} =-C_\alpha \int _0^\infty K_{1,\alpha }(T)\partial _R\bar{\Theta }\left( \frac{R}{T}\right) \left( \frac{R}{T}\right) ^{1-\alpha }\frac{\,\textrm{d}T}{T}. $$

The integrand vanishes outside $\frac{R}{r_2+\varepsilon }\le T\le \frac{R}{r_1-\varepsilon }$, and in $|T-1|\le \frac{1}{2}$ outside the annulus $\frac{1}{2}(r_1-\varepsilon )\le R\le \frac{3}{2}(r_2+\varepsilon )$. Thus, for any R>0 we can differentiate under the integral sign. By applying the Leibniz rule, we get

$$\begin{aligned} R^i\partial ^i_R\left( \frac{\bar{V}_\phi }{R}\right)&=\sum _{0\le j\le i} \frac{i!}{j!}\left( {\begin{array}{c}1-\alpha \\ i-j\end{array}}\right) \int _0^\infty K_{1,\alpha }(T)\partial _R^{j+1}\bar{\Theta }\left( \frac{R}{T}\right) \left( \frac{R}{T}\right) ^{1-\alpha +j} \frac{\,\textrm{d}T}{T}. \end{aligned}$$

Notice that all the integrals in the above sum can be expressed as

$$ \int _{r_1-\varepsilon }^{r_2+\varepsilon } K_{1,\alpha }\left( \frac{R}{S}\right) \partial _R^{j+1}\bar{\Theta }(S)S^{j-\alpha }\,\textrm{d}S \,\textrm{d}S. $$

The proof is concluded since $K_{1,\alpha }(\sigma )$ is bounded away from σ=1. □

The next proposition will be applied in the proof of Theorem 3.5. Recall that the eigenfunction $W=W_n e^{in\phi }$ is given by $W_n=h\partial _R\bar{\Theta }$, where $h\in L^2$ is the profile we found in the previous section.

#### Proposition 5.4

The eigenfunction $W\in U_n$ satisfies $W\in C_c^\infty$ with $\textrm{supp}(W_n)\subset [r_1-\varepsilon ,r_2+\varepsilon ]$.

#### Proof

Notice that $W_n=h\partial _r\bar{\Theta }=0$ outside the interval $B_\varepsilon ([r_1,r_2])$. Let us check that it is smooth inside the interval $I_\varepsilon =B_{2\varepsilon }([r_1,r_2])$. By construction, $W_n$ satisfies the eigenvalue equation (2.23) for b=0

5.18 $$\begin{aligned} \left( \frac{\bar{V}_\phi (R)}{C_\alpha R}- z\right) W_{n}(R) +\partial _R\bar{\Theta }(R)\int _0^\infty I_{n,\alpha }(S) W_{n}\left( \frac{R}{S}\right) \frac{\,\textrm{d}S}{S^{1+\alpha }} =0, \end{aligned}$$

where $\lambda =-inC_\alpha z$. Notice that the last integrand in (5.18) is supported on $\frac{R}{r_2+\varepsilon }\le S\le \frac{R}{r_1-\varepsilon }$. For α=1, the expansion provided in Lemma 4.3 implies that the integral operator in (5.18) gains a derivative in $L^2$. Since ℑz>0 we can invert the diagonal operator multiplying $W_n$ to conclude that $W_n \in H^1$. Using a bootstrapping argument, we further deduce that $W_n \in H^k$ for any k≥1. The cases $0 \le \alpha < 1$ are even more favorable, as the kernel $I_{n,\alpha }$ becomes more regular as α decreases as stated in Lemma 4.2. □

#### Remark 5.1

In the next section, we prove that $\bar{\Theta }$ is unstable in self-similar coordinates using Vishik’s spectral argument and semigroup theory. This approach could be avoided if one were able to solve the α-Rayleigh stability equation (2.23) directly for b>0. Given the simplicity of our vortex, this seems approachable but it could require substantial additional work. We are grateful to the anonymous referee for this interesting observation.

## Self-Similar Instability

In this section we prove Theorem 3.4, and provide growth bounds of the semigroup generated by $L_b$. From now on we consider n≥2 fixed.

### The Linear Operators

We split the linearization $L_b=L_{b,\alpha ,\bar{\Theta }}$ of the self-similar α-SQG equation around $\bar{\Theta }$ into

$$ L_b = (a-\alpha )b +T_b+K, $$

where $T_b$ is the transport operator

$$ T_b\Theta =-\bar{V}_b\cdot \nabla \Theta \quad \text {with}\quad \bar{V}_b =\bar{V}-b X, $$

and *K* is the operator

$$ K\Theta =-V\cdot \nabla \bar{\Theta } \quad \text {with}\quad V=-\nabla ^\perp \Lambda ^{\alpha -2}\Theta . $$

Here, $\bar{\Theta }\in C_c^\infty$ is the vortex from Theorem 3.3, and $\bar{V}$ is the corresponding velocity field. The domain of *K* is $D(K)=L_n^2$, and the domains of $L_b$ and $T_b$ are

$$ D(L_b)=D(T_b) =\{\Theta \in L_n^2\,:\,\textrm{div}(\bar{V}_b\Theta )\in L_n^2\}. $$

Thus, the operators under consideration are closed and densely defined in $L_n^2$. We recall that in Sect. 2.2, we verified that $U_{jn}$ is invariant under $T_b$ and *K* for every $j\in \mathbb {Z}$. The reason for considering the direct sum

$$\begin{aligned} L_n^2=\bigoplus _{j\in \mathbb {Z}}U_{jn} \end{aligned}$$

is that the quadratic term in the α-SQG equation lacks invariance in $U_{jn}$, but it is invariant in $L_n^2$. The invariance in $L_n^2$ follows from the following identity (see (2.27) and (6.6))

$$ \textrm{div}(V\Theta )(X) =\textrm{div}\left( e^{-\frac{2\pi i}{n}}(V\Theta )(e^{\frac{2\pi i}{n}} X)\right) =\textrm{div}(V\Theta )(e^{\frac{2\pi i}{n}} X). $$

More specifically, in Sect. 7 we will need the growth bound of the semigroup generated by $L_b$ acting on the full space $L_n^2$.

As we mentioned in the introduction, one of the key points in Vishik’s spectral analysis [129, 130] (as well as in [2, 31]) for the 2D Euler equation (α=0) is the fact that *K* is compact. This still holds for $0\le \alpha <1$, but no longer for α=1 (see Sect. 6.4). In Sect. 6.6, we show how to overcome this difficulty by appropriately decomposing the operator *K*.

### Spectral Analysis

In this section, we present the part of the analysis that is common to both cases: $0 \le \alpha < 1$ and α=1. For this purpose, we recall some classical results in Operator theory that will be useful during the analysis. We consider linear operators $A:D(A)\subset H\rightarrow H$ acting on some Hilbert space *H*, where *D*(*A*) is the domain of *A*. For a fixed *H*, we denote by $\mathcal {L}$ and $\mathcal {K}$ the space of bounded and compact operators, respectively. In the next sections we will consider $H=L_n^2$.

Firstly, we recall the stability of strongly continuous semigroups under bounded perturbations, which can be found in [68, Chapter III, Bounded Perturbation Theorem].

#### Proposition 6.1

Let *A* be a linear operator on *H* generating a strongly continuous semigroup, and $B\in \mathcal {L}.$ Then, A+B generates a strongly continuous semigroup.

For the proof of Theorem 3.4, we will decompose the operator $L_b$ into an operator $A_b$ that satisfies a series of properties detailed in the proposition below, and a compact operator *C*. The proof of this proposition can be found in Appendix A.1 and follows the same argument as in [2, 31]. We recall it here for the convenience of the reader. We remark that although this proposition could be further generalized, we choose to present the version that is most useful for our purposes. See Appendix A.1 and [68] for more details on operator theory and definitions. In particular, see Definition A.1 for the notion of contraction semigroup and (A.1) for the definition of the growth bound $\omega _0$.

#### Proposition 6.2

Assume that the following conditions hold:

1. Let $(A_b)$ be a family of linear operators on *H* generating contraction semigroups. Suppose that for any fixed $\tau \ge 0$ and W∈H, the map
  6.1 $$\begin{aligned} b\mapsto e^{\tau A_b}W \end{aligned}$$
  is continuous from [0,∞) to *H*.
2. Let *C* be a compact operator on *H*.
3. Let $L_b=A_b+C$. Suppose that there exists $\lambda _0$ with $\Re \lambda _0>0$ and $W_0\in D(L_0)$ such that
  $$ L_0W_0=\lambda _0 W_0. $$

Then, for every $b_0>0$, there exist $\lambda _b\in \mathbb {C}$ with $\Re \lambda _b=\omega _0(L_b)>\frac{\Re \lambda _0}{2}$ and $W_b\in D(L_b)$ for some $0<b\le b_0$ such that

$$ L_bW_b=\lambda _b W_b. $$

Furthermore, for every δ>0 there exists $C_\delta \ge 1$ such that

$$ \Vert e^{\tau L_b}\Vert _{\mathcal {L}} \le C_\delta e^{(\Re \lambda +\delta )\tau }, $$

for all $$\tau \ge 0$$.

#### Proof

See Appendix  8□

Therefore, to prove that the vortex $\bar{\Theta }$ is also self-similarly unstable, it will suffice to verify that the conditions (1)-(3) are satisfied for a suitable decomposition of $L_{b,\alpha ,\bar{\Theta }}$.

### The Transport Operator $T_b$

In this section, we show that the operator $T_b$ satisfies the condition (1) in Proposition 6.2. Although the proof is the same as in [31], we recall it here for its brevity and for the convenience of the reader.

#### Lemma 6.1

The operator $T_b$ generates a contraction semigroup $\{e^{\tau T_b}\}_{\tau \ge 0}$ with

6.2 $$\begin{aligned} \Vert e^{\tau T_b}\Vert _{\mathcal {L}} =e^{-b\tau }, \end{aligned}$$

for all $\tau \ge 0$. Furthermore, for any $\tau \ge 0$ and $\Theta \in L_n^2$, the map

$$ b\mapsto e^{\tau T_b}\Theta $$

is continuous from [0,∞) to $L_n^2$.

#### Proof

Notice that the velocity field $\bar{V}_b=\bar{V}-bX$ is Lipschitz. Hence, the first statement follows from the Cauchy-Lipschitz theory applied to the transport equation. In fact, it is well-known that the solution to the transport equation is given by

6.3 $$\begin{aligned} e^{\tau T_b} \Theta =\Theta \circ \bar{X}_b(\tau ,\cdot )^{-1}, \end{aligned}$$

where $\bar{X}_b$ is the flow map

6.4 $$\begin{aligned} \partial _\tau \bar{X}_b =\bar{V}_b(\bar{X}_b), \quad \quad \bar{X}_b|_{\tau =0}=\text {id}. \end{aligned}$$

For the second statement, by solving the ODE

$$ \partial _\tau J_{\bar{X}_b}=\textrm{div}(\bar{V}_b)J_{\bar{X}_b} \quad \text {with}\quad \textrm{div}(\bar{V}_b)=-2b, $$

we deduce that the Jacobian of the flow map equals

6.5 $$\begin{aligned} J_{\bar{X}_b}=e^{-2b\tau }. \end{aligned}$$

Therefore,

$$ \int _{\mathbb {R}^2}|e^{\tau T_b}\Theta |^2\,\textrm{d}X =e^{-2b\tau } \int _{\mathbb {R}^2}|\Theta |^2\,\textrm{d}Y, $$

where $X=\bar{X}_b(\tau ,Y)$. Due to (6.3), the second statement follows from the fact that the flow map $\bar{X}_b$ defined in (6.4) is continuous in *b*, which is a consequence of the continuity of $\bar{V}_b$ in *b*. More rigorously, we first prove the continuity in *b* for $\Theta \in L_n^2\cap C_c^\infty$ by applying pointwise convergence followed by the dominated convergence theorem. Then, we extend the result to the entire domain by density. □

### The Bounded Operator *K*

Analogously to the 2D Euler equation (α=0) we check that the α-Biot-Savart law (1.2) can be extended, for any $0\le \alpha \le 1$, within the subspace $L_n^2$. This is possible thanks to the following proposition. By slight abuse of notation, we will continue to denote the α-Biot-Savart operator as $V=-\nabla ^\perp \Lambda ^{\alpha -2}\Theta$.

#### Lemma 6.2

There exists C>0 such that

$$ R^{\alpha -1}\Vert V\Vert _{L^2(B_R)}+\Vert V\Vert _{\dot{H}^{1-\alpha }(\mathbb {R}^2)} \le C\Vert \Theta \Vert _{L^2(\mathbb {R}^2)} $$

for any R>0 and $\Theta \in L_n^2$, where $V=-\nabla ^\perp \Lambda ^{\alpha -2}\Theta$ for any $0\le \alpha \le 1$.

#### Proof

Let $\Theta \in L_n^2\cap C_c^\infty$. Firstly, by applying the Plancherel identity to the α-Biot-Savart law

$$ \hat{V} =-i\xi ^\perp |\xi |^{\alpha -2}\hat{\Theta }, $$

we deduce that

$$ \Vert V\Vert _{\dot{H}^{1-\alpha }} =\Vert \Theta \Vert _{L^2}. $$

Secondly, by applying the *n*-fold symmetry (2.27), we deduce that

6.6 $$\begin{aligned} V(X) =C_\alpha \int _{\mathbb {R}^2}\frac{(X-e^{-\frac{2\pi i k}{n}}Y)^\perp }{|X-e^{-\frac{2\pi i k}{n}}Y|^{2+\alpha }}\Theta (Y)\,\textrm{d}Y =e^{-\frac{2\pi i k}{n}} V(e^{\frac{2\pi i k}{n}}X). \end{aligned}$$

Therefore, by integrating *V* on the ball $B_R$, we get

$$ \int _{B_R}V(x)\,\textrm{d}x =e^{-\frac{2\pi i k}{n}}\int _{B_R}V(x)\,\textrm{d}x, $$

from which we deduce that

6.7 $$\begin{aligned} \int _{B_R}V(x)\,\textrm{d}x =\left( \frac{1}{n}\sum _{k=0}^{n-1}e^{-\frac{2\pi i k}{n}}\right) \int _{B_R}V(x)\,\textrm{d}x = 0. \end{aligned}$$

Thus, if we apply the fractional Poincaré inequality (see e.g. [128]), we deduce that

$$ R^{\alpha -1}\Vert V\Vert _{L^2(B_R)} \le C \Vert V\Vert _{\dot{H}^{1-\alpha }(B_R)} \le C\Vert V\Vert _{\dot{H}^{1-\alpha }(\mathbb {R}^2)}, $$

for any R>0. □

We take the opportunity to derive a lemma that will be helpful later in Sect. 7.

#### Lemma 6.3

Suppose m≥4. There exists C>0 such that

$$ \left\| \frac{V}{|X|}\right\| _{L^\infty }+\left\| \frac{\nabla \Theta }{|X|}\right\| _{L^\infty } \le C\Vert \Theta \Vert _{H^m} $$

for any $\Theta \in L_n^2\cap H^m$, where $V=-\nabla ^\perp \Lambda ^{\alpha -2}\Theta$.

#### Proof

Let $\Theta \in L_n^2\cap C_c^\infty$. By the Morrey inequality we have that $DV,D^2\Theta \in L^\infty$. In particular, *V* and $\nabla \Theta$ are continuous. Therefore, dividing both sides of (6.7) by $|B_R|$ and letting R→0, we deduce that V(0)=0. Consequently, by integrating *DV* over the segment with endpoints 0 and *X* we get

$$\begin{aligned} |V(X)|\le C |X|\Vert DV\Vert _{L^\infty }\le C |X|\Vert \Theta \Vert _{H^m}. \end{aligned}$$

Similarly, by the *n*-fold symmetry of Θ (recall (2.27)), we have that

$$\begin{aligned} \nabla \Theta (X)=e^{\frac{2\pi i k}{n}}\nabla \Theta \left( e^{\frac{2\pi i k}{n}}X\right) , \end{aligned}$$

thus we conclude that $\nabla \Theta (0)=0$. Hence, as before, $|\nabla \Theta (X)|\le C |X|\Vert \Theta \Vert _{H^m}.$ □

### Case $0\le \alpha <1$

In this section, we prove that $\bar{\Theta }$ is self-similarly unstable for the cases $0\le \alpha <1$. To this end, we will apply Proposition 6.2 to

$$ A_b =b(a-\alpha )+T_b, \quad \quad C=K. $$

#### Lemma 6.4

The operators $A_b=b(a-\alpha )+T_b$ satisfy the condition (1) in Proposition 6.2.

#### Proof

By applying Lemma 6.1 and the fact that the identity is a bounded operator, Proposition 6.1 implies that $A_b$ generates a strongly continuous semigroup. Then, for any $\Theta _0\in L_n^2\cap C_c^\infty$, there exists a unique global solution $\Theta =e^{\tau A_b}\Theta _0$ to

$$\partial _\tau \Theta =A_b\Theta , \quad \quad \Theta |_{\tau =0}=\Theta _0.$$

By applying the identity (recall $\textrm{div}(\bar{V}_b)=-2b$)

$$ \int _{\mathbb {R}^2}\Theta T_b\Theta \,\textrm{d}X =-\frac{1}{2}\int _{\mathbb {R}^2}\bar{V}_b\cdot \nabla |\Theta |^2\,\textrm{d}X =-b\int _{\mathbb {R}^2}|\Theta |^2\,\textrm{d}X, $$

the following energy estimate shows that $A_b=b(a-\alpha )+T_b$ is a contraction

$$\begin{aligned} \frac{1}{2}\partial _\tau \int _{\mathbb {R}^2}|\Theta |^2\,\textrm{d}X =\int _{\mathbb {R}^2}\Theta A_b\Theta \,\textrm{d}X = b(a-\alpha -1)\int _{\mathbb {R}^2}|\Theta |^2\,\textrm{d}X\le 0. \end{aligned}$$

In the last inequality we use our choice of $0<a<\varepsilon <1+\alpha$ (recall (2.8)). We remark that the same inequality is obtained in the full domain by density. The continuity in *b* follows analogously to that of the operator $T_b$ in the proof of Lemma 6.1. □

Secondly, we check that C=K satisfies condition (2) in Proposition 6.2.

#### Lemma 6.5

The operator *K* is compact in $L_n^2$ for $0\le \alpha <1$.

#### Proof

It follows by applying Lemma 6.2 and the (fractional) Rellich-Kondrachov Theorem (see e.g. [58, Sect. 7]). Recall that $K\Theta =-V\cdot \nabla \bar{\Theta }$ with $\bar{\Theta }$ smooth and compactly supported. □

Finally, notice that condition (3) in Proposition 6.2 was proved in Theorem 3.3. Since we have verified conditions (1)-(3), Proposition 6.2 implies that $\bar{\Theta }$ is self-similarly unstable.

### Case α=1

In this section, we prove that $\bar{\Theta }$ is self-similarly unstable for SQG. In this case we cannot apply Proposition 6.2 to $A_b=b(a-\alpha )+T_b$ since *K* is not compact for α=1. We bypass this obstacle by decomposing *K* into a skew-adjoint operator *S*, which preserves the growth bound of the semigroup, along with a commutator *C*, which turns out to be compact.

#### Proposition 6.3

It holds that

K=S+C,

where *S* is a skew-adjoint operator, and *C* is the commutator

6.8 $$\begin{aligned} C\Theta =\frac{1}{2}[\nabla \bar{\Theta },\Lambda ^{-1}\nabla ^\perp ]\Theta . \end{aligned}$$

#### Proof

This follows by integrating by parts:

$$\begin{aligned} \int _{\mathbb {R}^2}\Theta K\Theta \,\textrm{d}X&=\int _{\mathbb {R}^2}\Theta (\nabla ^\perp \Lambda ^{-1}\Theta )\cdot \nabla \bar{\Theta }\,\textrm{d}X\\&=-\int _{\mathbb {R}^2}\Lambda ^{-1}\Theta (\nabla ^\perp \Theta \cdot \nabla \bar{\Theta })\,\textrm{d}X\\&=-\int _{\mathbb {R}^2}\Theta \Lambda ^{-1}(\nabla ^\perp \Theta \cdot \nabla \bar{\Theta })\,\textrm{d}X. \end{aligned}$$

Therefore,

$$ \int _{\mathbb {R}^2}\Theta K\Theta \,\textrm{d}X =\int _{\mathbb {R}^2}\Theta C\Theta \,\textrm{d}X, $$

where

$$ C\Theta =\frac{1}{2}((\Lambda ^{-1}\nabla ^\perp \Theta )\cdot \nabla \bar{\Theta } -\Lambda ^{-1}(\nabla ^\perp \Theta \cdot \nabla \bar{\Theta })). $$

By definition, we have

6.9 $$\begin{aligned} \int _{\mathbb {R}^2}\Theta S\Theta \,\textrm{d}X =\int _{\mathbb {R}^2}\Theta (K-C)\Theta \,\textrm{d}X=0. \end{aligned}$$

This property is equivalent to being skew-adjoint. □

We will apply Proposition 6.2 to

$$ A_b =b(a-\alpha )+T_b+S, \quad \quad C=K-S. $$

#### Lemma 6.6

The commutator (6.8) is compact in $L_n^2$.

#### Proof

It is well known that *C* is a smoothing operator in $L^2$ (see e.g. [123]). Therefore, it is compact when restricted to $L^2(\Omega )$ for bounded subsets $\Omega \subset \mathbb {R}^2$. In order to extend the compactness to the whole plane, we will exploit the fact that $\bar{\Theta }$ is smooth and has compact support $B_{\bar{R}}$, with $\bar{R}=r_2+\varepsilon$. We split the commutator into

$$ C=C_1-C_2, $$

where

$$ C_1\Theta =\frac{1}{2}\Lambda ^{-1}\nabla ^\perp \cdot (\Theta \nabla \bar{\Theta }), \quad \quad C_2\Theta =\frac{1}{2}(\Lambda ^{-1}\nabla ^\perp \Theta \cdot \nabla \bar{\Theta }). $$

On the one hand, notice that $C_1$ is the Riesz transform of $H=\Theta \nabla \bar{\Theta }$

$$ C_1\Theta (X) =\frac{1}{4\pi } \int _{\mathbb {R}^2} \frac{(X-Y)^\perp }{|X-Y|^3}\cdot H(Y)\,\textrm{d}Y. $$

Therefore, for any $R>2\bar{R}$, we have

$$\begin{aligned} \int _{\mathbb {R}^2\setminus B_R} |C_1\Theta |^2\,\textrm{d}X&\le \frac{1}{(4\pi )^2}\int _{|X|\ge R} \left| \int _{|Y|\le \bar{R}}\frac{1}{|X-Y|^2}|H(Y)| \,\textrm{d}Y \right| ^2\,\textrm{d}X\\&\le \frac{1}{\pi ^2}\int _{|X|\ge R}\frac{\,\textrm{d}X}{|X|^4} \left| \int _{|Y|\le \bar{R}}|H(Y)|\,\textrm{d}Y\right| ^2 \le \left( \frac{\bar{R}}{R}\right) ^2\Vert H\Vert _{L^2}^2, \end{aligned}$$

where in the second line we applied that

$$ |X-Y|\ge |X|-|Y|\ge \frac{1}{2}|X|. $$

Observe that

$$ \Vert H\Vert _{L^2} \le \Vert \nabla \bar{\Theta }\Vert _{L^\infty } \Vert \Theta \Vert _{L^2}. $$

On the other hand, since $C_2\Theta$ vanishes outside the support of $\bar{\Theta }$, we have

$$ \int _{\mathbb {R}^2\setminus B_R}|C_2\Theta |^2\,\textrm{d}X=0. $$

These estimates allow us to conclude that *C* is compact on the whole plane. Let $\Theta _n$ be a sequence that is uniformly bounded in $L^2$. Since *C* is compact when restricted to $L^2(\Omega )$ for bounded subsets $\Omega \subset \mathbb {R}^2$, by a diagonal argument, we can extract a subsequence of $C\Theta _n$ that converges to some *D* in $L^2$ on any ball $B_N$. By relabeling if necessary, we denote this subsequence by $C\Theta _n$, with a slight abuse of notation. Let ε>0. Since $\Theta _n$ is uniformly bounded in $L^2$, the bounds we obtained for $C_1$ and $C_2$ ensure that we can take $N\in \mathbb {N}$ big enough such that

$$ \Vert C\Theta _n-D\Vert _{L^2(\mathbb {R}^2\setminus B_N)} \le \Vert C\Theta _n\Vert _{L^2(\mathbb {R}^2\setminus B_N)} +\Vert D\Vert _{L^2(\mathbb {R}^2\setminus B_N)} \le \frac{\varepsilon }{2}, $$

uniformly in *n*. Once *N* is fixed, we can take $n_0\in \mathbb {N}$ big enough such that

$$ \Vert C\Theta _n-D\Vert _{L^2(B_N)} \le \frac{\varepsilon }{2}, $$

for all $n\ge n_0$. Therefore, $C\Theta _n\rightarrow D$ in $L^2$. We have proved that *C* is compact in $L^2$, and thus also in $L_n^2$. □

#### Lemma 6.7

The operators $A_b=b(a-\alpha )+T_b+S$ satisfy the condition (1) in Proposition 6.2.

#### Proof

By applying Lemma 6.4 and that $S=K-C\in \mathcal {L}$, Proposition 6.1 implies that $A_b$ generates a strongly continuous semigroup. Similarly to the proof of Lemma 6.4, but now applying that *S* is skewadjoint (recall (6.9)), the following energy estimate on $\Theta =e^{\tau A_b}\Theta _0$ shows that $A_b=b(a-\alpha )+T_b+S$ is a contraction

$$\begin{aligned} \frac{1}{2}\partial _\tau \int _{\mathbb {R}^2}|\Theta |^2\,\textrm{d}X&=\int _{\mathbb {R}^2}\Theta A_b\Theta \,\textrm{d}X =\int _{\mathbb {R}^2}\Theta (A_b-S)\Theta \,\textrm{d}X \\&= b(a-\alpha -1)\int _{\mathbb {R}^2}|\Theta |^2\,\textrm{d}X\le 0. \end{aligned}$$

In the last inequality we use our choice of $0<a<\varepsilon <1+\alpha$ (recall (2.8)). The continuity in *b* follows analogously to that of the operator $T_b$ in the proof of Lemma 6.1. □

Since condition (1) has been verified in Lemma 6.7, condition (2) in Lemma 6.6, and condition (3) in Theorem 3.3, Proposition 6.2 implies that $\bar{\Theta }$ is self-similarly unstable.

### The Eigenfunction

In this section we prove that the eigenfunction

$$ W_b\in \textrm{Ker}(L_b-\lambda _b) $$

associated with the eigenvalue $\lambda _b$ appearing in Proposition 6.2 is smooth and compactly supported.

Given m≥5, we fix $b=b_m$ satisfying

6.10 $$\begin{aligned} 0<b <\frac{\Re \lambda _b}{m+3}. \end{aligned}$$

Thus, from now on, we will denote *W* and λ instead of $W_b$ and $\lambda _b$ to alleviate the notation.

Since $U_{jn}$ is invariant under $L_b$ and

$$ W=\sum _{j\in \mathbb {Z}} W_{jn}(R)e^{ijn \theta }, $$

each (non-zero) projection $W_{jn}(R)e^{ijn \theta }\in U_{jn}$ is also an eigenfunction. For j=0, since $L_b W_{0}=\lambda _b W_{0}$ reads as

$$ b(a+R\partial _R)W_{0}=\lambda _b W_{0}, $$

We necessarily have that $W_{0}=0$. Therefore, $0\ne W_{jn}(r)e^{ijn\theta }\in \textrm{Ker}(L_b-\lambda )\cap U_{jn}$ for some j≠0. Moreover, since $\textrm{Ker}(L_b-\lambda )$ is finite dimensional (see Lemma A.2), $W_{jn}$ is null for all but a finite number of (non-zero) *j*’s.

#### Proposition 6.4

Let $W\in \textrm{Ker}(L_b-\lambda )\cap U_{jn}$ with j≠0. Then,

$$ W\in C^\infty (\mathbb {R}^2\setminus \{0\}) \cap C_c^\gamma (\mathbb {R}^2), $$

with $$\gamma =(\frac{\Re \lambda }{b}-(a-\alpha ))\ge m+2$$. Moreover,

$$ W_{jn}(R) =C_0 R^{\frac{\lambda -b(a-\alpha )+ijn C}{b}}e^{-\frac{ijn}{b}U(R)}, $$

for all $R\le \frac{r_1}{2}$, where $C_0$ is a constant, and *C* and *U*(*R*) are given in Proposition 5.3.

#### Proof

The self-similar stability equation (2.23) can be rewritten as

6.11 $$\begin{aligned}  &   \left( (\lambda -b(a-\alpha ))+ijn\frac{\bar{V}_\phi }{R}-bR\partial _R\right) W_{jn}\nonumber \\  &   \quad +in\, C_\alpha \partial _R\bar{\Theta }\int _0^\infty I_{jn,\alpha }(S) W_{jn}\left( \frac{R}{S}\right) \frac{\,\textrm{d}S}{S^{1+\alpha }} =0. \end{aligned}$$

We remark that (6.11) is well defined since $W\in D(L_b)$ by construction. On the interval $(0,r_1-\varepsilon )$, we have $\partial _R\bar{\Theta }=0$. Thus, $W_{jn}$ solves an ODE that can be solved explicitly by applying the formula for $\bar{V}_\phi$ from Proposition 5.3. We obtain that, on the interval $(0,r_1-\varepsilon )$, it holds that

$$ W_{jn}(R) =C_0 R^{\frac{(\lambda -b(a-\alpha ))+ijn C}{b}}e^{-\frac{ijn}{b}U(R)} $$

for some new constant $C_0$. On the interval $(r_2+\varepsilon ,\infty )$, since $\partial _R\bar{\Theta }=0$, we deduce that

$$ |W_{jn}(R)| =C_1 R^{\frac{\Re \lambda -b(a-\alpha )}{b}}. $$

In this case, since $W_{jn}\in L^2$ and by (6.10), necessarily $C_1=0$. As a consequence, we have $\textrm{supp}(W_{jn})\subset [0,r_2+\varepsilon ]$. We have checked that $W_{jn}$ is smooth outside the interval $B_\varepsilon ([r_1,r_2])$. Let us check that it is smooth inside the interval $I_\varepsilon =B_{2\varepsilon }([r_1,r_2])$. Notice that the last integrand in (6.11) is supported on $S\ge \frac{R}{r_2+\varepsilon }$. Hence, it follows from (6.11) that $W_{jn}\in H^1(I_\varepsilon )$. By bootstrapping, the same formula allows us to prove that $W_{jn}\in H^k(I_\varepsilon )$ for any k≥1. □

## Non-Linear Instability

In this section we prove Theorem 3.5. We emphasize that the proof works for b>0, which establishes the Sobolev non-uniqueness result (Theorem 3.1), as well as for b=0, which demonstrates the existence of unstable vortices exhibiting non-uniqueness at t=-∞ (Theorem 1.2). In this section, m≥5 is fixed (except for the definition of $Y^m$ and the results related to its properties) and a corresponding *b* is also fixed in the regime

7.1 $$\begin{aligned} 0\le b <\frac{\Re \lambda }{m+3}. \end{aligned}$$

In order to prove Theorem 3.5, we construct a sequence of approximate solutions $\Theta _k^{(q)}$ to (2.24) that satisfy the decay condition (2.26) uniformly in both *k* and *q*, and then we pass to the limit in a suitable Sobolev space. Indeed, we consider the weighted Hilbert space $H_\omega ^m$ given by the norm

$$ \Vert f\Vert _{H_\omega ^m} :=\Vert f\omega \Vert _{L^2} +\sum _{0<|K|\le m}\Vert \partial _X^K f\omega \Vert _{L^2}, $$

where ω is the standard radial weight

$$ \omega (X) =\langle X\rangle ^2 =1+R^2. $$

Firstly, for every $k\in \mathbb {N}$, we consider the unique solution $\Theta _k^{\text {cor}}$ to (2.24)

7.2 $$\begin{aligned} (\partial _\tau - L_b)\Theta _k^{\text {cor}} +\underbrace{(V^{\text {lin}}+\epsilon V_k^{\text {cor}})\cdot \nabla (\Theta ^{\text {lin}}+\epsilon \Theta _k^{\text {cor}})}_{\mathcal {F}_k} =0, \end{aligned}$$

coupled with the initial condition (replacing (2.25))

7.3 $$\begin{aligned} \Theta _k^{\text {cor}}|_{\tau =-k}=0. \end{aligned}$$

The local existence and uniqueness of this solution is guaranteed by [35]. We recall that for b>0, in the original system of coordinates, we have that

$$ \theta _{k} =\theta _0+\epsilon \theta ^{\text {lin}} +\epsilon ^2\theta _k^{\text {cor}} $$

is a solution to the α-SQG equ. (1.1) with the initial condition

7.4 $$\begin{aligned} \theta _{k}|_{t=t_k}=(\theta _0+\epsilon \theta ^{\text {lin}})(t_k) =(abt_k)^{\frac{\alpha }{a}-1}(\bar{\Theta }+\epsilon \Theta ^{\text {lin}}(-k)), \end{aligned}$$

where $t_k=e^{-abk}$, and with the forcing

7.5 $$\begin{aligned} f=-(abt)^{\frac{\alpha }{a}-2}b((a-\alpha )+X\cdot \nabla )\bar{\Theta }. \end{aligned}$$

The corresponding $\Theta ^{\text {cor}}_k$ solves (7.2) and (7.3).

Secondly, for every $k\in \mathbb {N}$, we recover $\Theta _k^{\text {cor}}$ as the limit of the following iterative scheme. Starting from $\Theta _k^{(0)}=0$, we define $\Theta _k^{(q)}$ for any $q\in \mathbb {N}$ by

7.6 $$\begin{aligned} (\partial _\tau - L_b)\Theta _k^{(q)} +\underbrace{(V^{\text {lin}}+\epsilon V_k^{(q-1)})\cdot \nabla (\Theta ^{\text {lin}}+\epsilon \Theta _k^{(q)})}_{\mathcal {F}_k^{(q)}} =0, \end{aligned}$$

coupled with the initial condition

7.7 $$\begin{aligned} \Theta _k^{(q)}|_{\tau =-k}=0. \end{aligned}$$

### Remark 7.1

On the one hand, since $\bar{\Theta } \in C_c^\infty$ (Theorem 3.3) and $\Theta ^{\text {lin}} \in C_c^{m+2}$ (Proposition 5.4 for b=0, and Proposition 6.4 for b>0), the initial datum satisfies $\bar{\Theta } + \epsilon \Theta ^{\text {lin}}(-k) \in C_c^{m+2}$. On the other hand, for b>0, the force (7.5) remains smooth and compactly supported for all $t \ge t_k$. Recall that f=0 for b=0. Moreover, by construction, both the initial datum and the force have finite Hamiltonian. Therefore, for all *k*, *q*, the solution $\Theta _k^{(q)}$ to this linearized system belongs to $C([-k,T], H^{m+2} \cap \dot{H}^{\frac{\alpha -2}{2}})$ for all T>-k.

As we explained in the intro, the use of the Duhamel formula allows gaining extra exponential decay, but at the cost of having to control the bound under a stronger norm. In Sect. 7.2 we introduce the energy space $Y^m\hookrightarrow H_\omega ^m\cap L_n^2$. Since we need to work in $Y^m$ (instead of $H_\omega ^m$) we must ensure that our approximate solution remains in this space globally in time.

### Proposition 7.1

Let $k,q\in \mathbb {N}$. For every T>-k, the unique solution $\Theta _k^{(q)}$ to (7.6), (7.7) satisfies

$$ \Theta _k^{(q)}\in L^\infty ([-k,T];Y^{m+1})\cap C([-k,T];Y^{m}). $$

Moreover, $\Theta _k^{(q)}$ remains compactly supported.

### Proof

We will prove it in Sect. 7.3 after introducing the space $Y^m$. □

Due to Proposition 7.1 and recalling (7.7), given some fixed $\delta _0$ in the regime

$$\begin{aligned} 0<\delta _0<c_{(m,0)}:=2^{-\frac{m(m+3)}{2}}, \end{aligned}$$

we can define $-k<\tau _k^{(q)}\le 0$ to be the largest non-positive time such that

7.8 $$\begin{aligned} \Vert \Theta ^{(q)}_k(\tau )\Vert _{Y^m}\le e^{(1+\delta _0)\Re \lambda \tau } \end{aligned}$$

for any $-k\le \tau \le \tau _k^{(q)}$. The purpose of the notation $c_{(m,0)}$ will become clear later in the text. Our task is to show that the times $\tau _k^{(q)}$ satisfying (7.8) do not diverge to -∞ as $k,q\rightarrow \infty$. For this purpose, we will use a bootstrapping argument to improve the bound (7.8). This is the content of the following lemma, whose proof will take most of this section.

### Lemma 7.1

There exists C>0, independent of *k* and *q*, such that

7.9 $$\begin{aligned} \Vert \Theta ^{(q)}_k(\tau )\Vert _{Y^m}\le C e^{(1+c_{(m,0)})\Re \lambda \tau } \end{aligned}$$

for all $$-k\le \tau \le \min _{p\le q}\tau _k^{(p)}$$.

### Proof

We will prove it in Sects. 7.4-7.8. □

As a corollary of this lemma, we obtain the required lower bound for $\tau _k^{(q)}$.

### Corollary 7.1

It holds that

7.10 $$\begin{aligned} \bar{\tau } :=\inf _{k,q}\tau _k^{(q)} > -\infty . \end{aligned}$$

### Proof

We prove it by contradiction. Suppose that $\bar{\tau }=-\infty$. Then, there must exist a sequence satisfying

7.11 $$\begin{aligned} 0>\tau _{k_j}^{(q_j)}\rightarrow -\infty , \end{aligned}$$

as $j\rightarrow \infty$. We can assume, without loss of generality, that

7.12 $$\begin{aligned} \tau _{k_j}^{(q_j)}=\min _{p\le q_j}\tau _{k_j}^{(p)}. \end{aligned}$$

Otherwise, it would suffice to replace $q_j$ by the *p* that minimizes (7.12). By definition of $\tau _k^{(q)}$ in (7.8), we have

7.13 $$\begin{aligned} \left\| \Theta ^{(q_j)}_{k_j}\left( \tau _{k_j}^{(q_j)}\right) \right\| _{Y^m}= e^{(1+\delta _0)\Re \lambda \tau _{k_j}^{(q_j)}}. \end{aligned}$$

Hence, by combining (7.9) with (7.13), we get

$$ C e^{(c_{(m,0)}-\delta _0)\Re \lambda \tau _{k_j}^{(q_j)}}\ge 1, $$

which contradicts (7.11), due to $\delta _0<c_{(m,0)}$. □

In the next section, we show how Theorem 3.5 is proven once we know that (7.10) holds. Therefore, in order to conclude it will remain to check Lemma 7.1.

### Proof of Theorem 3.5

Firstly, we take the limit $q\rightarrow \infty$, to recover the solutions $\Theta _k^{\text {cor}}$. By the embedding $Y^m\hookrightarrow H_\omega ^m$ (see Lemmas 7.4 and 7.5) and (7.8), these solutions satisfy

$$ \Vert \Theta _k^{\text {cor}}(\tau )\Vert _{H_\omega ^m}\lesssim \Vert \Theta _k^{\text {cor}}(\tau )\Vert _{Y^m} \le \liminf _{q\rightarrow \infty }\Vert \Theta _k^{(q)}(\tau )\Vert _{Y^m} \le e^{(1+\delta _0)\Re \lambda \tau } $$

for all $-k\le \tau \le \bar{\tau }$, with $\bar{\tau }>-\infty$ by Corollary 7.1. Secondly, we take the limit $k\rightarrow \infty$. With this regularity, the sequence $\Theta ^{\text {cor}}_k$ converges to a solution $\Theta ^{\text {cor}}$ of (2.24), which satisfies

$$ \Vert \Theta ^{\text {cor}}(\tau )\Vert _{H_\omega ^m} \le \liminf _{k\rightarrow \infty } \Vert \Theta _k^{\text {cor}}(\tau )\Vert _{H_\omega ^m} \lesssim e^{(1+\delta _0)\Re \lambda \tau } $$

for all $\tau \le \bar{\tau }$. Notice that the Hamiltonian remains finite since the sequence of classical solutions $\Theta _k^{\text {cor}}$ satisfy the Hamiltonian identity, which provides a uniform bound in *k* due to Remark 7.1.

### Weighted Energy Space

As we explained in Sect. 3, it will be necessary to switch between Cartesian and polar coordinates. The following lemmas relate the partial derivatives of any order in both coordinate systems. We present a proof in Appendix A.2 for completeness.

#### Lemma 7.2

(From Cartesian to Polar derivatives) For any multi-index $K=(k_1,k_2)$ with |K|>0, it holds that

$$ \partial _X^Kf =\sum _{0<|J|\le |K|}p_J^K\frac{\partial _R^{j_1}\partial _\phi ^{j_2}f}{R^{|K|-j_1}}, $$

where $p_J^K=p_J^K(\cos \phi ,\sin \phi )$ is a |*K*|-homogeneous polynomial.

#### Lemma 7.3

(From Polar to Cartesian derivatives) For any multi-index $J=(j_1,j_2)$ with |J|>0, it holds that

$$ \partial _R^{j_1}\partial _\phi ^{j_2}f =\sum _{\begin{array}{c} 0<|K|\le |J| \\ j_1\le |K| \end{array}} q_K^J R^{|K|-j_1}\partial _X^K f, $$

where $q_K^J=q_K^J(\cos \phi ,\sin \phi )$ is a |*K*|-homogeneous polynomial.

Given $m\in \mathbb {N}$, we define the energy subspaces $Y^m$ and $Z^m$ of $L_n^2$ given by the norms

$$\begin{aligned} \Vert f\Vert _{Y^m}&:=\Vert f\omega \Vert _{L^2} +\sum _{0<|J|\le m}\left\| \frac{\partial _R^{j_1}\partial _\phi ^{j_2}f}{R^{m-j_1}}\omega \right\| _{L^2},\\ \Vert f\Vert _{Z^m}&:=\Vert f\omega \Vert _{L^2} +\sum _{0<|K|\le m}\left\| \frac{\partial _X^K f}{R^{m-|K|}}\omega \right\| _{L^2}, \end{aligned}$$

where $L^2=L^2(\mathbb {R}^2,\textrm{d}X)$ in Cartesian coordinates. In Lemmas 7.4 and 7.5 we will see that indeed

$$ Y^m=Z^m\hookrightarrow H^m_\omega , $$

as well as other embeddings that will be crucial during the energy estimates. Notice that $Y^1=Z^1=H_\omega ^1$, while in general $Y^m=Z^m\subsetneq H_\omega ^m$ for m≥2.

#### Embeddings

Note that the inequality

7.14 $$\begin{aligned} \Vert f\Vert _{H^m}\lesssim \Vert f\Vert _{Z^m} \end{aligned}$$

follows by the definition of $Z^m$. Indeed, $\Vert f\Vert _{L^2}\le \Vert f\omega \Vert _{L^2}\le \Vert f\Vert _{Z^m}$ and, for any |K|=m, $\Vert \partial _X^K f\Vert _{L^2}\le \Vert \partial _X^K f \, \omega \Vert _{L^2}\le \Vert f\Vert _{Z^m}$.

##### Lemma 7.4

For any $0\le |K|\le m$ and $f\in Z^m$,

7.15 $$\begin{aligned} \Vert \partial _X^K f \, \omega \Vert _{H^{m-|K|}}\lesssim \Vert f\Vert _{Z^m}. \end{aligned}$$

In particular,

$$\begin{aligned} \Vert f\Vert _{H^m_\omega }\lesssim \Vert f\Vert _{Z^m}. \end{aligned}$$

##### Proof

Note that the case |K|=m follows directly by the definition of $Z^m$. We start by proving the case |K|=0. Since $\Vert f \omega \Vert _{L^2}\le \Vert f\Vert _{Z^m}$, it is enough to see that $\Vert \partial ^L_X (f\omega )\Vert _{L^2}\lesssim \Vert f\Vert _{Z^m}$ for every |L|=m. Therefore, we let |L|=m and apply the Leibniz rule

$$\begin{aligned} \partial ^L_X (f\omega )=\sum _{(0,0)\le J\le L}{L \atopwithdelims ()J}\partial ^{L-J}_X f \, \partial ^J_X\omega . \end{aligned}$$

Note that, since $\omega (X)=1+X_1^2+X_2^2$, we have $\partial ^J_X\omega =0$ for J≠(0,0),(1,0),(0,1),(2,0),(0,2). We deal with these cases separately. If J=(0,0), then

$$\begin{aligned} \Vert \partial ^{L-J}_X f \, \partial ^J_X\omega \Vert _{L^2}=\Vert \partial ^{L}_X f \, \omega \Vert _{L^2}\le \Vert f\Vert _{Z^m}. \end{aligned}$$

If J=(1,0) or J=(0,1) we have $\partial ^J_X\omega =2X_1$ or $2X_2$, respectively. Therefore, since |L-J|=m-1,

$$\begin{aligned} \Vert \partial ^{L-J}_X f \, \partial ^J_X\omega \Vert _{L^2}=\left\| \frac{\partial ^{L-J}_X f}{R} \frac{R\partial ^J_X\omega }{\omega } \omega \right\| _{L^2}\le 2\left\| \frac{\partial ^{L-J}_X f}{R^{m-|L-J|}} \omega \right\| _{L^2}\lesssim \Vert f\Vert _{Z^m}. \end{aligned}$$

Lastly, if J=(2,0) or J=(0,2) we have $\partial _X^J\omega =2$, thus, by (7.14),

$$\begin{aligned} \Vert \partial ^{L-J}_X f \, \partial ^J_X\omega \Vert _{L^2}=2\Vert \partial ^{L-J}_X f\Vert _{L^2} \lesssim \Vert f\Vert _{Z^m}. \end{aligned}$$

We now proceed inductively on |*K*|. Let 1<|K|≤m and suppose that (7.15) holds for every $K^{\prime }$ with $|K^{\prime }|=|K|-1$. Take |J|=1 with J≤K, then

$$\begin{aligned} \Vert \partial ^K_Xf \omega \Vert _{H^{m-|K|}}&=\Vert \partial ^{J}_X(\partial ^{K-J}_X f\omega )-\partial ^{K-J}_Xf\partial _X^J\omega \Vert _{H^{m-|K|}}\\&=\left\| \partial ^{J}_X(\partial ^{K-J}_X f\omega )-\partial ^{K-J}_Xf\frac{\partial _X^J\omega }{\omega }\omega \right\| _{H^{m-|K|}}\\&\lesssim \Vert \partial ^{K-J}_X f\omega \Vert _{H^{m-(|K|-1)}}+\Vert \partial ^{K-J}_X f \omega \Vert _{H^{m-|K|}}\\&\lesssim \Vert \partial _X^{K-J}f\omega \Vert _{H^{m-|K-J|}} \lesssim \Vert f\Vert _{Z^m}, \end{aligned}$$

where K-J plays the role of $K^{\prime }$. □

##### Lemma 7.5

It holds that

$$ \Vert f\Vert _{Z^m} \simeq \Vert f\Vert _{Y^m}. $$

Moreover,

$$\begin{aligned} \Vert f\Vert _{Y^m}&\simeq \Vert f\Vert _{\bar{Y}^m}:=\Vert f\omega \Vert _{L^2} +\sum _{0<|J|\le r+j_2\le m}\left\| \frac{\partial _R^{j_1}\partial _\phi ^{j_2}f}{R^{m-r}}\omega \right\| _{L^2},\\ \Vert f\Vert _{Z^m}&\simeq \Vert f\Vert _{\bar{Z}^m}:=\Vert f\omega \Vert _{L^2} +\sum _{0<|K|\le r\le m}\left\| \frac{\partial _X^K f}{R^{m-r}}\omega \right\| _{L^2}. \end{aligned}$$

##### Proof

To prove the equivalences of norms we prove the following sequence of inequalities:

$$\Vert f\Vert _{Z^m} \lesssim \Vert f\Vert _{Y^m} \le \Vert f\Vert _{\bar{Y}^m} \lesssim \Vert f\Vert _{\bar{Z}^m} \lesssim \Vert f\Vert _{Z^m}.$$

To prove $\Vert f\Vert _{Z^m}\lesssim \Vert f\Vert _{Y^m}$ we use Lemma 7.2 to write

$$ \frac{\partial _X^K f}{R^{m-|K|}}\omega =\sum _{0<|J|\le |K|}p_J^K\frac{\partial _R^{j_1}\partial _\phi ^{j_2}f}{R^{m-j_1}}\omega $$

for any 0<|K|≤m and then apply the definition of $Y^m$.

The inequality $\Vert f\Vert _{Y^m}\le \Vert f\Vert _{\bar{Y}^m}$ is trivial. For the inequality $\Vert f\Vert _{\bar{Y}^m}\lesssim \Vert f\Vert _{\bar{Z}^m}$ let $0<|J|\le r+j_2\le m$ and use Lemma 7.3 to write

$$\frac{\partial _R^{j_1}\partial _\phi ^{j_2}f}{R^{m-r}}\omega =\sum _{\begin{array}{c} 0<|K|\le |J| \\ j_1\le |K| \end{array}} q_K^J \frac{\partial _X^K f}{R^{m-(r-j_1+|K|)}}\omega .$$

Then the inequality follows by using the definition of $\bar{Z}^m$ since $|K|\le r-j_1+|K|\le m$.

Finally, $\Vert f\Vert _{\bar{Z}^m}\lesssim \Vert f\Vert _{Z^m}$ follows by decoupling

$$ \frac{\left| \partial _X^K f\right| }{R^{m-r}}\omega \le \frac{\left| \partial _X^K f\right| }{R^{m-|K|}}\omega 1_B +\left| \partial _X^K f\right| \omega 1_{B^c}\le \Vert f\Vert _{Z^m}+\Vert f\Vert _{H^m_\omega }, $$

for any $0<|K|\le r\le m$, and then applying Lemma 7.4. □

The next lemma relates $H_\omega ^m$ with classical Sobolev spaces. The embedding is not optimal, but since *m* is arbitrarily large, we present a simple version that is enough for our purposes.

##### Lemma 7.6

For all $$1\le p\le \infty $$,

$$ H_\omega ^m\hookrightarrow W^{m-2,p}. $$

##### Proof

Let $f\in H_\omega ^m$. On the one hand, for any $0\le |K|\le m$,

$$ \Vert \partial ^K f\Vert _{L^q} \le \Vert \partial ^K f\omega \Vert _{L^2}\Vert \omega ^{-1}\Vert _{L^{\frac{2q}{2-q}}}, $$

with $\omega ^{-1}\in L^{\frac{2q}{2-q}}$ for $\frac{2}{3}<q\le 2$. Therefore, $f\in W^{m,q}$ for all $1\le q\le 2$. On the other hand, since $f\in H^m$, the Morrey inequality implies that $f\in W^{m-2,\infty }$. Hence, by interpolation, we have $f\in W^{m-2,p}$ for all $1\le p\le \infty$. □

In the next lemma, we show that $Y^{m+1}(\Omega )$ is compact in $Y^m(\Omega )$ for bounded subsets $\Omega \subset \mathbb {R}^2$ and arbitrary $m\in \mathbb {N}$, where $Y^m(\Omega )$ is given as $Y^m$ but replacing $L^2(\mathbb {R}^2,\textrm{d}X)$ by $L^2(\Omega ,\textrm{d}X)$.

##### Lemma 7.7

The embedding $Y^{m+1}(\Omega )\hookrightarrow Y^m(\Omega )$ is compact for any bounded subset $\Omega \subset \mathbb {R}^2$.

##### Proof

Given $f\in Y^{m+1}(\Omega )$ and 0<|J|≤m, we denote

$$ f_J=\frac{\partial ^J f}{R^{m-j_1}}=\frac{\partial _R^{j_1}\partial _\phi ^{j_2}f}{R^{m-j_1}}. $$

On the one hand,

$$ \partial _R f_J =\frac{\partial ^{J+(1,0)} f}{R^{(m+1)-(j_1+1)}} -(m-j_1)\frac{\partial ^J f}{R^{m+1-j_1}}. $$

On the other hand,

$$ \frac{\partial _\phi f_J}{R} =\frac{\partial ^{J+(0,1)} f}{R^{(m+1)-j_1}}. $$

Therefore, $f_J\in H^1(\Omega )$. The statement follows by applying that the embedding $H^1(\Omega )\hookrightarrow L^2(\Omega )$ is compact. The weight ω does not play any role here. □

Next, we check that the vortex and eigenfunction belong to these weighted energy spaces.

##### Lemma 7.8

It holds that

$$ \bar{\Theta }\in \bigcap _{m\in \mathbb {N}}Y^m. $$

##### Proof

By construction, we have that $\bar{\Theta }\in C_c^\infty (\mathbb {R}^2)$ with $\bar{\Theta }(X)$ constant on $|X|\le \frac{1}{4}$. □

##### Lemma 7.9

There exists C>0 such that

$$ \Vert \Theta ^{\text {lin}}(\tau )\Vert _{Y^{m+2}} \le C e^{\Re \lambda \tau }, $$

for all $$\tau \in \mathbb {R}$$.

##### Proof

Recall that $\Theta ^{\text {lin}}(\tau ,X)=\Re (e^{\lambda _b\tau }W_b(X))$ (see Sect. 6.7). The statement follows by Proposition 5.4 for b=0, and by Proposition 6.4 for b>0. □

We conclude this section by controlling the $L^\infty$ norm of the velocity $V=-\nabla ^\perp \Lambda ^{\alpha -2}\Theta$, namely

7.16 $$\begin{aligned} \Vert V\Vert _{L^\infty }\lesssim \Vert \Theta \Vert _{H_\omega ^m}\lesssim \Vert \Theta \Vert _{Y^m}. \end{aligned}$$

This follows from the following lemma in combination with Lemmas 7.4- 7.6:

##### Lemma 7.10

For α=0 and $2<p\le \infty$, there exists C>0 such that

$$ \Vert V\Vert _{L^\infty } \le C(\Vert \Theta \Vert _{L^1}+\Vert \Theta \Vert _{L^p}). $$

For $0<\alpha \le 1$ and ϵ>0, there exists C>0 such that

$$ \Vert V\Vert _{L^\infty }\le C\Vert \Theta \Vert _{H^{1+\epsilon }}. $$

##### Proof

For α=1, simply $V\in H^{1+\epsilon }\hookrightarrow L^\infty$ by the Morrey inequality. For $0\le \alpha <1$, we bound

$$\begin{aligned} |V(X)|\le C_\alpha \int _{\mathbb {R}^2}\frac{|\Theta (Y)|}{|X-Y|^{1+\alpha }}\,\textrm{d}Y. \end{aligned}$$

We split $\mathbb {R}^2$ into |X-Y|>1 and |X-Y|<1. Let α=0. In the exterior domain,

$$ \int _{|X-Y|>1}\frac{|\Theta (Y)|}{|X-Y|}\,\textrm{d}Y \le \Vert \Theta \Vert _{L^1}. $$

In the interior domain,

$$ \int _{|X-Y|<1}\frac{|\Theta (Y)|}{|X-Y|}\,\textrm{d}Y \le \Vert \Theta \Vert _{L^p} \left( 2\pi \int _1^\infty \frac{R\,\textrm{d}R}{R^{\frac{p}{p-1}}}\right) ^{\frac{p-1}{p}} =\left( 2\pi \frac{p-1}{p-2}\right) ^{\frac{p-1}{p}} \Vert \Theta \Vert _{L^p}.$$

Now let 0<α<1. In the exterior domain,

$$ \int _{|X-Y|>1}\frac{|\Theta (Y)|}{|X-Y|^{1+\alpha }}\,\textrm{d}Y \le \Vert \Theta \Vert _{L^2}\left( 2\pi \int _1^\infty \frac{R\,\textrm{d}R}{R^{2(1+\alpha )}}\right) ^{\nicefrac {1}{2}} =\sqrt{\frac{\pi }{\alpha }}\Vert \Theta \Vert _{L^2}. $$

In the interior domain,

$$ \int _{|X-Y|<1}\frac{|\Theta (Y)|}{|X-Y|^{1+\alpha }}\,\textrm{d}Y \le 2\pi \Vert \Theta \Vert _{L^\infty }\int _0^1\frac{R\,\textrm{d}R}{R^{1+\alpha }} =\frac{2\pi }{1-\alpha }\Vert \Theta \Vert _{L^\infty }, $$

with $$\Theta \in H^{1+\epsilon }\hookrightarrow L^\infty $$ for ϵ>0. □

### Proof of Proposition 7.1

We begin by writing equation (7.6) in terms of the deviation in the original coordinates

7.17 $$\begin{aligned} \partial _t\tilde{\theta } +v_\epsilon \cdot \nabla \tilde{\theta } +\tilde{v}\cdot \nabla \theta _0 =0, \end{aligned}$$

coupled with the initial condition (7.7)

$$ \tilde{\theta }|_{t=t_k} =\theta ^{\text {lin}}(t_k), $$

where we have abbreviated

$$ \tilde{\theta } =\theta ^{\text {lin}}+\epsilon \theta _k^{(q)}, \quad \quad \tilde{v} =v^{\text {lin}}+\epsilon v_k^{(q)}, $$

and also

$$ \theta _\epsilon =\theta _0+\epsilon \theta ^{\text {lin}}+\epsilon ^2 \theta _k^{(q-1)}, \quad \quad v_\epsilon =v_0+\epsilon v^{\text {lin}}+\epsilon ^2 v_k^{(q-1)}. $$

Notice that $\tilde{\theta }$ and $\tilde{v}$ correspond to step *q*, while $\theta _\epsilon$ and $v_\epsilon$ refer to step q-1. When it is not clear from the context, we will use the superscript *q* again. We remark that for b=0, we were already in the original variables and therefore do not need to change to lowercase letters. By a slight abuse of notation, we denote both cases using lowercase letters since the proof is exactly the same.

By Remark 7.1, the solution to (7.6) in physical variables satisfies

7.18 $$\begin{aligned} \tilde{\theta } \in C([t_k,T];H^{m+2}), \quad \quad \partial _t\tilde{\theta }\in L^\infty ([t_k,T];H^{m+1}), \end{aligned}$$

for all $T>t_k$. Moreover, this solution remains compactly supported.

Next, we perform energy estimates in the weighted energy space $Y^{m+1}=Z^{m+1}$. To avoid the singularity at r=0, we will carry out the energy estimates uniformly with respect to a parameter ε>0 that desingularizes the weight $r^{-(m+1-|K|)}$. We notice that for b=0 this desingularization would not be necessary since the initial datum is constant in a neighborhood of the origin.

Given a multi-index $1\le |K|\le m+1$, we deduce from (7.17) that

$$ (\partial _t+v_\epsilon \cdot \nabla ) \left( \frac{\partial ^K\tilde{\theta }}{(\varepsilon +r)^{m+1-|K|}}\omega \right) +h=0, $$

where $$h=h_1+h_2$$ with

$$ h_1= \frac{\partial ^K(v_\epsilon \cdot \nabla \tilde{\theta })}{(\varepsilon +r)^{m+1-|K|}}\omega -v_\epsilon \cdot \nabla \left( \frac{\partial ^K\tilde{\theta }}{(\varepsilon +r)^{m+1-|K|}}\omega \right) , \quad \quad h_2=\frac{\partial ^K(\tilde{v}\cdot \nabla \theta _0)}{(\varepsilon +r)^{m+1-|K|}}\omega . $$

Since we have (7.18) and ε>0, we can integrate by parts to deduce that

$$ \partial _t\left\| \frac{\partial ^K\tilde{\theta }}{(\varepsilon +r)^{m+1-|K|}}\omega \right\| _{L^2} \le \Vert h\Vert _{L^2}, $$

where we have applied that $v_\epsilon$ is divergence free.

Concerning $h_2$, since $\nabla \theta _0$ vanishes outside an annulus, we have

$$ \Vert h_2\Vert _{L^2}\le C\Vert \tilde{\theta }\Vert _{H^{m+1}}, $$

where *C* depends on $\theta _0$, *m*, *k*, *q*, and *T*, but not on ε.

Concerning $h_1$, by applying

$$ \nabla \left( \frac{\omega }{(\varepsilon +r)^{m+1-|K|}}\right) =\left( \frac{2r}{(\varepsilon +r)^{m+1-|K|}}-\frac{m+1-|K|}{(\varepsilon +r)^{m+2-|K|}}\omega \right) e_r $$

we split $h_1=h_{1,1}+h_{1,2}$ with

$$\begin{aligned} h_{1,1}= &   \frac{\partial ^K(v_\epsilon \cdot \nabla \tilde{\theta }) -v_\epsilon \cdot \nabla \partial ^K\tilde{\theta }}{(\varepsilon +r)^{m+1-|K|}},\\ h_{1,2}= &   \left( \frac{2r}{(\varepsilon +r)^{m+1-|K|}}-\frac{m+1-|K|}{(\varepsilon +r)^{m+2-|K|}}\omega \right) (v_\epsilon )_r\partial ^K\tilde{\theta }. \end{aligned}$$

The term $h_{1,2}$ can be bounded by

$$ \Vert h_{1,2}\Vert _{L^2} \le C\left\| \frac{v_\epsilon }{r}\right\| _{L^\infty } \left\| \frac{\partial ^K\tilde{\theta }}{(\varepsilon +r)^{m+1-|K|}}\omega \right\| _{L^2}, $$

where *C* only depends on *m*. The velocity term can be bounded by $\Vert \theta _\epsilon \Vert _{H^m}$ (Lemma 6.3). Concerning $h_{1,1}$, by applying the Leibniz rule, we get

$$ h_{1,1} =\sum _{(0,0)\ne L\le K}\left( {\begin{array}{c}K\\ L\end{array}}\right) h_{K,L}, \quad \text {with}\quad h_{K,L} =\partial ^Lv_\epsilon \cdot \frac{\nabla \partial ^{K-L}\tilde{\theta }}{(\varepsilon +r)^{m+1-|K|}}. $$

The terms with multi-indices 0<|L|≤m are bounded by

$$ \Vert h_{K,L}\Vert _{L^2} \le \Vert \partial ^Lv_\epsilon \Vert _{L^\infty } \left\| \frac{\nabla \partial ^{K-L}\tilde{\theta }}{(\varepsilon +r)^{m+1-|K|}}\right\| _{L^2}. $$

The first term is bounded by $\Vert \theta _\epsilon \Vert _{H^{m+2}}$. The second term can be bounded, by splitting the integral into the regions $|x|\le 1-\varepsilon$ and |x|>1-ε, as follows

$$\begin{aligned} \left\| \frac{\nabla \partial ^{K-L}\tilde{\theta }}{(\varepsilon +r)^{m+1-|K|}}\right\| _{L^2}&\le \left\| \frac{\nabla \partial ^{K-L}\tilde{\theta }}{(\varepsilon +r)^{m+1-|K-L|}}\right\| _{L^2(B_{1-\varepsilon })} + \Vert \nabla \partial ^{K-L}\tilde{\theta }\Vert _{L^2(\mathbb {R}^2\setminus B_{1-\varepsilon })}. \end{aligned}$$

If |L|=m+1, necessarily L=K and thus

$$ \Vert h_{K,K}\Vert _{L^2} \le \Vert \partial ^Kv_\epsilon \Vert _{L^2} \Vert \nabla \tilde{\theta }\Vert _{L^\infty } \le \Vert \theta _\epsilon \Vert _{H^{m+1}}\Vert \tilde{\theta }\Vert _{H^m}. $$

Summing over all multi-indices *K*, we deduce that the semi-norm

$$ f_\varepsilon = \sum _{0<|K|\le m+1}\left\| \frac{\partial _X^K\tilde{\theta }}{(\varepsilon +r)^{m+1-|K|}}\right\| _{L^2} $$

satisfies the bound

$$ \partial _t f_\varepsilon \le \Vert \theta _\epsilon \Vert _{H^{m+2}}f_\varepsilon +P(\Vert \theta _\epsilon \Vert _{H^{m+2}},\Vert \tilde{\theta }\Vert _{H^{m+1}}), $$

where *P* is a polynomial. By applying the Grönwall inequality, we deduce that

7.19 $$\begin{aligned} \begin{aligned} f_\varepsilon (t) \le&f_\varepsilon (t_k)\exp \left( \int _{t_k}^t\Vert \theta _\epsilon (s)\Vert _{H^{m+2}}\,\textrm{d}s\right) \\&+\int _{t_k}^t P(\Vert \theta _\epsilon \Vert _{H^{m+2}},\Vert \tilde{\theta }\Vert _{H^{m+1}})(s)\exp \left( \int _{s}^t\Vert \theta _\epsilon (t')\Vert _{H^{m+2}}\,\textrm{d}t'\right) \,\textrm{d}s. \end{aligned} \end{aligned}$$

Finally, by applying Lemma 7.9

$$ f_\varepsilon (t_k)\le f_0(t_k)\le \Vert \theta ^{\text {lin}}(t_k)\Vert _{Y^{m+1}}, $$

we deduce, by passing to the limit $\varepsilon \rightarrow 0$ in (7.19), that

$$ \tilde{\theta }\in L^\infty ([t_k,T];Y^{m+1}) $$

for all $T>t_k$. Therefore, by applying the Aubin-Lions lemma (recall Lemma 7.7) and that $\tilde{\theta }$ is compactly supported, we deduce that

$$ \tilde{\theta }\in C([t_k,T];Y^{m}), $$

for all $$T>t_k$$.

### Baseline $L^2$ estimate

In this section, we improve the exponential decay of $\Vert \Theta ^{(q)}_k\Vert _{L^2}$. In the next sections, we will use this baseline estimate to address the $Y^m$-norm for the proof of Lemma 7.1.

#### Lemma 7.11

There exists C>0 such that

7.20 $$\begin{aligned} \Vert \Theta ^{(q)}_k(\tau )\Vert _{L^2}\le C e^{2\Re \lambda \tau }, \end{aligned}$$

for all $$-k\le \tau \le \min _{p\le q}\tau _k^{(p)}$$.

#### Proof

The solution to (7.6) satisfies the Duhamel formula

$$ \Theta _k^{(q)}(\tau ) =-\int _{-k}^{\tau }e^{(\tau -\tau ')L_b}\mathcal {F}_k^{(q)}(\tau ')\,\textrm{d}\tau '. $$

By applying (7.14) and (7.16), we estimate

$$ \Vert \mathcal {F}_k^{(q)}\Vert _{L^2} \le \Vert V^{\text {lin}}+\epsilon V_k^{(q-1)}\Vert _{L^\infty } \Vert \nabla (\Theta ^{\text {lin}}+\epsilon \Theta _k^{(q)})\Vert _{L^2} \le C\Vert \Theta ^{\text {lin}}+\epsilon \Theta _k^{(q)}\Vert _{Y^m}^2. $$

Thus, by applying Lemma 7.9 for $\Theta ^{\text {lin}}$, and the bound (7.8) for $\Theta _k^{(q)}$, we get

$$ \Vert \mathcal {F}_k^{(q)}(\tau )\Vert _{L^2} \le Ce^{{2\Re \lambda \tau }},$$

for all $-k\le \tau \le \min _{p\le q}\tau _k^{(p)}$. Finally, by Proposition 6.2, for some fixed $0<\delta \le \frac{\Re \lambda }{2}$ and $C=C_\delta \ge 1$, we have

7.21 $$\begin{aligned} \Vert \Theta ^{(q)}_k(\tau )\Vert _{L^2}\le C e^{(\Re \lambda +\delta )\tau }\int _{-k}^{\tau } e^{{(\Re \lambda -\delta )s}}\,\textrm{d}s\le C e^{{2\Re \lambda \tau }}, \end{aligned}$$

for all $$-k\le \tau \le \min _{p\le q}\tau _k^{(p)}$$. □

### Inductive Energy Estimates

In this section we deal with the weighted norms appearing in the $Y^m$-norm for the proof of Lemma 7.1. We start by rewriting (7.6) as

7.22 $$\begin{aligned} \partial _\tau \Theta -b(a-\alpha )\Theta +(\bar{V}-bX+\tilde{V})\cdot \nabla \Theta +V\cdot \nabla \bar{\Theta }+\tilde{V}\cdot \nabla \Theta ^{\text {lin}}=0, \end{aligned}$$

where we have abbreviated

7.23 $$\begin{aligned} \Theta =\Theta _k^{(q)}, \quad \quad V=V_k^{(q)}, \end{aligned}$$

and also

7.24 $$\begin{aligned} \tilde{\Theta }=\epsilon \Theta ^{\text {lin}}+\epsilon ^2 \Theta _k^{(q-1)}, \quad \quad \tilde{V}=\epsilon V^{\text {lin}}+\epsilon ^2 V_k^{(q-1)}. \end{aligned}$$

Notice that Θ and *V* correspond to step *q*, while $\tilde{\Theta }$ and $\tilde{V}$ refer to step q-1. When it is not clear from the context, we will use the superscript *q* again.

We start by proving that it is possible to improve the bound of the weighted $L^2$ norm in $Y^m$ by using the baseline estimate in $L^2$.

#### Lemma 7.12

There exists C>0 such that

7.25 $$\begin{aligned} \Vert \Theta (\tau )\omega \Vert _{L^2}\le C e^{2\Re \lambda \tau }, \end{aligned}$$

for all $$-k\le \tau \le \min _{p\le q}\tau _k^{(p)}$$.

#### Proof

By applying that $X=Re_R$, $\bar{V}=\bar{V}_\phi e_\phi$ and $\nabla \omega =2Re_R$, we deduce that

$$\begin{aligned}&(\bar{V}-bX+\tilde{V})\cdot \nabla \Theta \omega -(\bar{V}-bX+\tilde{V})\cdot \nabla (\Theta \omega ) =(\bar{V}-bX+\tilde{V})\cdot \\&\quad \nabla \omega \Theta =(-bR+\tilde{V}_R)2R\Theta . \end{aligned}$$

Hence, multiplying (7.22) by ω, we deduce that

7.26 $$\begin{aligned} \partial _\tau \Theta \omega -b\left( a-\alpha +\frac{2R^2}{\omega }\right) \Theta \omega +(\bar{V}-bX+\tilde{V})\cdot \nabla (\Theta \omega )+\mathcal {H}=0, \end{aligned}$$

where $$\mathcal {H}=\bar{\mathcal {H}}+\mathcal {H}_1+\mathcal {H}_2$$ with

$$ \bar{\mathcal {H}} =V\cdot \nabla \bar{\Theta }\omega , \quad \quad \mathcal {H}_1 =\frac{\tilde{V}_R}{R}\frac{2R^2}{\omega }\Theta \omega , \quad \quad \mathcal {H}_2 =\tilde{V}\cdot \nabla \Theta ^{\text {lin}}\omega . $$

Multiplying (7.26) by $\Theta \omega$ and integrating over $\mathbb {R}^2$, we deduce that

$$\begin{aligned} \frac{1}{2}\partial _\tau \Vert \Theta \omega \Vert _{L^2}^2&\le b(2+a-(1+\alpha ))\Vert \Theta \omega \Vert _{L^2}^2 +\left| \int _{\mathbb {R}^2}\mathcal {H}\Theta \omega \,\textrm{d}X\right| \\&\le 2b\Vert \Theta \omega \Vert _{L^2}^2 +\Vert \mathcal {H}\Vert _{L^2}\Vert \Theta \omega \Vert _{L^2}, \end{aligned}$$

where we have applied Proposition 7.1, that the velocities are divergence free, $\textrm{div}X=2$, and that $0<a<\varepsilon <1+\alpha$ by (2.8). Therefore, the Grönwall inequality implies that

$$ \Vert \Theta \omega \Vert _{L^2} \le \int _{-k}^{\tau } e^{2b(\tau -\tau ')}\Vert \mathcal {H}(\tau ')\Vert _{L^2}\,\textrm{d}\tau '. $$

For the first term, since $\bar{\Theta }$ is smooth and supported in a ball of radius $\bar{R}=r_2+\varepsilon$, by applying Lemmas 6.2 and 7.11, we get

$$ \Vert \bar{\mathcal {H}}\Vert _{L^2} \le \Vert \nabla \bar{\Theta }\omega \Vert _{L^\infty }\Vert V\Vert _{L^2(B_{\bar{R}})} \lesssim \Vert \Theta \Vert _{L^2} \lesssim e^{2\Re \lambda \tau }. $$

For the quadratic term $\mathcal {H}_1$, by applying Lemmas 6.3, 7.4 and 7.5, and the bound (7.8), we get

$$ \Vert \mathcal {H}_1\Vert _{L^2} \le 2\left\| \frac{\tilde{V}_R}{R}\right\| _{L^\infty }\Vert \Theta \omega \Vert _{L^2} \lesssim \Vert \Theta \Vert _{Y^m}^2 \lesssim e^{2\Re \lambda \tau }. $$

Similarly for the other quadratic term $\mathcal {H}_2$, by applying also (7.16) and Lemma 7.9, we get

$$ \Vert \mathcal {H}_2\Vert _{L^2} \lesssim \Vert \tilde{V}\Vert _{L^\infty } \Vert \nabla \Theta ^{\text {lin}}\omega \Vert _{L^2} \lesssim e^{2\Re \lambda \tau }. $$

We conclude the proof by applying (7.1). □

We continue by improving the bound of the weighted partial derivatives in $Y^m$. In general, we want to estimate terms of the form

$$ \left\| \frac{\partial ^J\Theta }{R^{m-j_1}}\omega \right\| _{L^2}, $$

where $J=(j_1,j_2)$ is a multi-index and $\partial ^J$ is the corresponding partial derivative in polar coordinates

$$ \partial ^J =\partial _R^{j_1}\partial _\phi ^{j_2}. $$

To simplify the notation, we will omit the subscript (R,ϕ) when working with partial derivatives in polar coordinates. The subscript *X* will be used for partial derivatives in Cartesian coordinates to distinguish them from the previous case.

In the next proposition, we prove an energy estimate that holds for all multi-indices *J*.

#### Proposition 7.2

(Energy estimate) For any multi-index *J* with |J|>0, it holds that

$$ \left\| \frac{\partial ^J\Theta (\tau )}{R^{m-j_1}}\omega \right\| _{L^2}^2 \le 2\int _{-k}^{\tau }e^{2b(m+2)(\tau -\tau ')} \left| \int \mathcal {H}_{J}\frac{\partial ^J\Theta }{R^{m-j_1}}\omega \,\textrm{d}X\right| (\tau ')\,\textrm{d}\tau ', $$

for all $-k\le \tau \le \tau _k^{(q)}$, where we split

$$ \mathcal {H}_{J} =\mathcal {H}_J^{\text {rad}} +\mathcal {H}_{J}^{\text {com}} +\mathcal {H}_J^{\text {quad}}, $$

into the radial term

$$ \mathcal {H}_J^{\text {rad}} =\frac{\omega }{R^{m-j_1}}\sum _{0<i_1\le j_1}\left( {\begin{array}{c}j_1\\ i_1\end{array}}\right) \partial ^{i_1}_R\left( \frac{\bar{V}_\phi }{R}\right) \partial ^{j_1-i_1}_R\partial _\phi ^{j_2+1}\Theta , $$

the commutator term

$$ \mathcal {H}_{J}^{\text {com}} =\frac{\partial ^J(V\cdot \nabla \bar{\Theta })}{R^{m-j_1}}\omega , $$

and the quadratic term

$$ \mathcal {H}_J^{quad} =\frac{\partial ^J(\tilde{V}\cdot \nabla \Theta )}{R^{m-j_1}}\omega -\tilde{V}\cdot \nabla \left( \frac{\partial ^J\Theta }{R^{m-j_1}}\omega \right) +\frac{\partial ^J(\tilde{V}\cdot \nabla \Theta ^{\text {lin}})}{R^{m-j_1}}\omega . $$

#### Proof

Firstly, recall that

$$ X\cdot \nabla \Theta =R\partial _R\Theta . $$

By applying the Leibniz rule on the radial variable, we get

7.27 $$\begin{aligned} \partial ^J(X\cdot \nabla \Theta ) =\sum _{i_1\le j_1}\left( {\begin{array}{c}j_1\\ i_1\end{array}}\right) \partial _R^{i_1}R\partial _R^{j_1-i_1+1}\partial _\phi ^{j_2}\Theta =X\cdot \nabla \partial ^J\Theta +j_1\partial ^J\Theta . \end{aligned}$$

The first term corresponds to $i_1=0$, and the second to $i_1=1$ (which equals zero when $j_1=0$). The remainder terms vanish because $\partial _R^{i_1}R=0$ for $i_1>1$.

The identity

7.28 $$\begin{aligned} \nabla \left( \frac{1}{R^{m-j_1}}\omega \right) =\left( \frac{2R}{R^{m-j_1}}-\frac{m-j_1}{R^{m-j_1+1}}\omega \right) e_R, \end{aligned}$$

combined with (7.27), implies that

$$ \frac{\partial ^J(X\cdot \nabla \Theta )}{R^{m-j_1}}\omega =X\cdot \nabla \left( \frac{\partial ^J\Theta }{R^{m-j_1}}\omega \right) +\left( m-\frac{2R^2}{\omega }\right) \frac{\partial ^J\Theta }{R^{m-j_1}}\omega . $$

Secondly, recall that

$$ \bar{V}\cdot \nabla \Theta =\frac{\bar{V}_\phi }{R}\partial _\phi \Theta . $$

By again applying the Leibniz rule on the radial variable, we get

$$\begin{aligned} \partial ^J(\bar{V}\cdot \nabla \Theta ) =\sum _{i_1\le j_1} \left( {\begin{array}{c}j_1\\ i_1\end{array}}\right) \partial _R^{i_1} \left( \frac{\bar{V}_\phi }{R}\right) \partial _R^{j_1-i_1}\partial _\phi ^{j_1+1}\Theta . \end{aligned}$$

Therefore, splitting into the term with $i_1=0$ and the terms with $i_1>0$, we get

$$ \frac{\partial ^J(\bar{V}\cdot \nabla \Theta )}{R^{m-j_1}}\omega =\bar{V}\cdot \nabla \left( \frac{\partial ^J\Theta }{R^{m-j_1}}\omega \right) +\mathcal {H}_J^{\text {rad}}. $$

Therefore, we deduce from (7.22) that the following equation holds

$$\begin{aligned}&\partial _\tau \left( \frac{\partial ^J\Theta }{R^{m-j_1}}\omega \right) -b\left( m-\frac{2R^2}{\omega }+a-\alpha \right) \frac{\partial ^J\Theta }{R^{m-j_1}}\omega +(\bar{V}-bX+\tilde{V})\\&\quad \cdot \nabla \left( \frac{\partial ^J\Theta }{R^{m-j_1}}\omega \right) +\mathcal {H}_J=0. \end{aligned}$$

Multiplying it by $\frac{\partial ^J\Theta }{R^{m-j_1}}\omega$ and integrating over $\mathbb {R}^2$, we deduce that

$$ \frac{1}{2}\partial _\tau \left\| \frac{\partial ^J\Theta }{R^{m-j_1}}\omega \right\| _{L^2}^2 \le b(m+2+a-(1+\alpha ))\left\| \frac{\partial ^J\Theta }{R^{m-j_1}}\omega \right\| _{L^2}^2 +\left| \int _{\mathbb {R}^2}\mathcal {H}_J\frac{\partial ^J\Theta }{R^{m-j_1}}\omega \,\textrm{d}X\right| , $$

where we have applied Proposition 7.1, that the velocities are divergence free and that $\textrm{div}X=2$. Finally, recall that $0<a<\varepsilon <1+\alpha$ by (2.8). □

In addition to the standard notation I≤J for $i_1\le j_1$ and $i_2 \le j_2$, we define the following well-ordering of multi-indices:

$$\begin{aligned} I\prec J \ \ \Longleftrightarrow \ \ |I|<|J|, \ \text { or } \ |I|=|J| \text { and } i_1<j_1. \end{aligned}$$

In fact, it is possible to number these multi-indices by

$$ \text {ind}(J):=\frac{|J|(|J|+1)}{2}+j_1, $$

that is, I≺J if and only if $\text {ind}(I)<\text {ind}(J)$. We define that

$$ c_J:=2^{-\text {ind}(J)}. $$

#### Proposition 7.3

(Inductive energy estimate) Let *J* be a multi-index with 0<|J|≤m. Assume that for all $(0,0)\ne I\prec J$ there exists $C_I>0$ such that

7.29 $$\begin{aligned} \left\| \frac{\partial ^I\Theta (\tau )}{R^{m-i_1}}\omega \right\| _{L^2} \le C_I e^{(1+c_I)\Re \lambda \tau }, \end{aligned}$$

for all $-k\le \tau \le \min _{p\le q}\tau _k^{(p)}$. Then, there exists $D_J>0$ such that

7.30 $$\begin{aligned} \left| \int \mathcal {H}_{J}\frac{\partial ^J\Theta }{R^{m-j_1}}\omega \,\textrm{d}X\right| (\tau ) \le D_J e^{2(1+c_J)\Re \lambda \tau }, \end{aligned}$$

for all $-k\le \tau \le \min _{p\le q}\tau _k^{(p)}$. As a consequence, the bound (7.29) is also satisfied for *J*. In particular, no assumption is needed to obtain (7.29) for J=(0,1).

#### Proof

We prove the bound (7.30) for $\mathcal {H}^{\text {rad}}$, $\mathcal {H}^{\text {com}}$ and $\mathcal {H}^{\text {quad}}$ in Sections 7.7, 7.8 and 7.9, respectively. Assuming that we have already proven (7.30), we show that (7.29) is also satisfied for *J*. By applying Proposition 7.2 and the bound (7.30), together with (7.1), we get

7.31 $$\begin{aligned} \begin{aligned} \left\| \frac{\partial ^J\Theta }{R^{m-j_1}}\omega \right\| _{L^2}^2&\le D_J\int _{-k}^{\tau } e^{2b(m+2)(\tau -\tau ')+2(1+c_J)\Re \lambda \tau '}\,\textrm{d}\tau '\\&\le \frac{1}{2}\frac{D_J}{(1+c_J)\Re \lambda -b(m+2)} e^{2(1+c_J)\Re \lambda \tau }, \end{aligned} \end{aligned}$$

for all $$-k\le \tau \le \min _{p\le q}\tau _k^{(p)}$$. □

### Proof of Lemma 7.1

We apply the inductive energy estimate (Proposition 7.3) on the multi-indices *J*. Notice that for the first multi-index J=(0,1) there are no $(0,0)\ne I\prec J$, thus Proposition 7.3 immediately implies (7.29) for J=(0,1). We now make the induction hypothesis. Given a multi-index J≻(0,1), suppose that (7.29) is satisfied for any multi-index *I* with I≺J. Then, we can apply Proposition 7.3 to deduce that (7.29) is also satisfied for *J*. Once we have reached the last multi-index J=(m,0), we have proven that (7.29) is satisfied for any multi-index *J* with 0<|J|≤m. Since $c_J$ is decreasing with respect to the ordering ≺, we deduce (7.9) and thus Lemma 7.1 as well. Recall that the $L^2$ part of the $Y^m$ norm was estimated in Lemma 7.12.

### Radial term

In this section we prove the bound (7.30) for

$$\begin{aligned} \mathcal {H}_{J}^{\text {rad}}&=\sum _{0<i_1\le j_1}\left( {\begin{array}{c}j_1\\ i_1\end{array}}\right) R^{i_1}\partial ^{i_1}_R\left( \frac{\bar{V}_\phi }{R}\right) \frac{\partial ^{j_1-i_1}_R\partial _\phi ^{j_2+1}\Theta }{R^{m-(j_1-i_1)}}\omega . \end{aligned}$$

Recall that Θ corresponds to step *q* according to (7.23). Notice that $\mathcal {H}_{J}^{\text {rad}}=0$ if $j_1=0$. Otherwise, since $(j_1-i_1,j_2+1)\prec J$ for every $0<i_1\le j_1$ we can apply the assumption (7.29) which, combined with Lemma 5.8, yields

$$\begin{aligned} \big \Vert \mathcal {H}_{J}^{\text {rad}}\big \Vert _{L^2}&\lesssim \sum _{0<i_1\le j_1}\left\| R^{i_1}\partial ^{i_1}_R\left( \frac{\bar{V}_\phi }{R}\right) \right\| _{L^\infty }\left\| \frac{\partial ^{j_1-i_1}_R\partial _\phi ^{j_2+1}\Theta }{R^{m-(j_1-i_1)}}\omega \right\| _{L^2}\\&\lesssim \sum _{0<i_1\le j_1}e^{(1+c_{(j_1-i_1,j_2+1)})\Re \lambda \tau }\lesssim e^{(1+2c_{J})\Re \lambda \tau }, \end{aligned}$$

where we used that $c_L\ge 2c_J$ whenever L≺J. Finally, by applying (7.8), we get

$$ \left| \int \mathcal {H}_{J}^{\text {rad}}\frac{\partial ^J\Theta }{R^{m-j_1}}\omega \,\textrm{d}X\right| \le \Vert \mathcal {H}_{J}^{\text {rad}}\Vert _{L^2} \Vert \Theta \Vert _{Y^m} \lesssim e^{2(1+c_J)\Re \lambda \tau }. $$

### Commutator term

In this section we prove the bound (7.30) for $\mathcal {H}_{J}^{\text {com}}$. Recall that Θ and *V* are both at step *q* according to (7.23). We have to estimate the integral

$$ A=\int \frac{\partial ^J(V\cdot \nabla \bar{\Theta })}{R^{m-j_1}}\frac{\partial ^J\Theta }{R^{m-j_1}}\omega ^2 . $$

By rewriting polar into Cartesian coordinates (Lemma 7.3) we get

$$\begin{aligned} A&=\int \frac{\omega ^2}{R^{2(m-j_1)}} \Bigg \lbrace \sum _{\begin{array}{c} 0<|K|\le |J| \\ j_1\le |K| \end{array}} q_K^J R^{|K|-j_1}\partial _X^K(V\cdot \nabla \bar{\Theta })\Bigg \rbrace \Bigg \lbrace \sum _{\begin{array}{c} 0<|L|\le |J| \\ j_1\le |L| \end{array}} q_L^J R^{|L|-j_1}\partial _X^L\Theta \Bigg \rbrace \\&=\sum _{\begin{array}{c} 0<|K|\le |J| \\ j_1\le |K| \end{array}} \sum _{\begin{array}{c} 0<|L|\le |J| \\ j_1\le |L| \end{array}} \int \frac{\omega ^2 q_K^J q_L^J}{R^{2m-(|K|+|L|)}} \partial _X^K(V\cdot \nabla \bar{\Theta })\partial _X^L\Theta . \end{aligned}$$

We remark that the weights $\frac{\omega ^2}{R^{2m-(|K|+|L|)}}$ are negligible because $\nabla \bar{\Theta }$ is smooth and supported in an annulus. The terms for which either |*K*| or |*L*| are less than |*J*| can be bounded easily. By applying the Leibniz rule in the term $\partial _X^K(V\cdot \nabla \bar{\Theta })$, all the terms for which some derivatives hit $\nabla \bar{\Theta }$ can also be bounded easily. Taking into account all these observations we can decompose

A=B+G,

into a bad term

$$ B =\sum _{|K|=|L|=|J|} \int \frac{\omega ^2 q_K^J q_L^J}{R^{2m-(|K|+|L|)}} (\partial _X^KV\cdot \nabla \bar{\Theta })\partial _X^L\Theta , $$

and a good term *G* satisfying the bound

7.32 $$\begin{aligned} |G|\lesssim \Vert \Theta \Vert _{H^{|J|-1}}\Vert \Theta \Vert _{H^{|J|}}. \end{aligned}$$

We remark that we have used that the $L^2$-norm of the velocity in $B_{\bar{R}}$ with $\bar{R}=r_2+\varepsilon$ is bounded by $\Vert \Theta \Vert _{L^2}$ (Lemma 6.2). To estimate *B*, it is crucial that the function

$$ H_{K,L}^J:=\frac{\omega ^2 q_K^J q_L^J}{R^{2m-(|K|+|L|)}}\nabla \bar{\Theta } \in C_c^\infty , $$

and that $H_{L,K}^J=H_{K,L}^J$. By writing $V=-R^\perp _\alpha \Theta$ (denote $R_\alpha =\nabla (-\Delta )^{\nicefrac {\alpha }{2}-1}$), we can express *B* by,

$$\begin{aligned} B =&-\sum _{|K|=|L|=|J|} \int R^\perp _\alpha (\partial _X^K\Theta )\cdot (H_{K,L}^J\partial _X^L\Theta )\\ =&\sum _{|K|=|L|=|J|} \int (\partial _X^K\Theta ) \cdot R^\perp _\alpha (H_{K,L}^J\partial _X^L\Theta )\\ =&\sum _{|K|=|L|=|J|} \int (H_{K,L}^J\partial _X^K\Theta )\cdot R^\perp _\alpha (\partial _X^L\Theta )\\&+\sum _{|K|=|L|=|J|} \int \partial _X^K\Theta \left\{ R^\perp _\alpha (H_{K,L}^J\partial _X^L\Theta ) -H_{K,L}^J\cdot R^\perp _\alpha (\partial _X^L\Theta ) \right\} . \end{aligned}$$

where, for the second equality, we have used that $R_\alpha ^{\perp }$ is skew adjoint and in the last equality we have added and subtracted $\int (H_{K,L}^J\partial _X^K\Theta )\cdot R^\perp _\alpha (\partial _X^L\Theta )$. Notice that, by exchanging *K* and *L*, it follows that the first term equals -B. Therefore, 2*B* is indeed equal to the last term. It is easier to express it in terms of a commutator. That is,

$$ B=\frac{1}{2} \sum _{|K|=|L|=|J|} \int \partial _X^K\Theta \left[ R^\perp _\alpha ,H_{K,L}^J\right] \partial _X^L\Theta . $$

As a consequence, using the smoothing properties of the commutator, we obtain that (see e.g. [123])

7.33 $$\begin{aligned} |B| \le C\Vert \Theta \Vert _{H^{|J|-1}}\Vert \Theta \Vert _{H^{|J|}}, \end{aligned}$$

where *C* only depends on *m* and $\bar{\Theta }$. Finally, recall that by (7.14), $Y^m\hookrightarrow H^m$. Therefore, we can use (7.32) (7.33) to obtain the first inequality below. For the second inequality we use the hypothesis (7.29) (the weights are irrelevant because of the support of Θ) together with (7.8). Thus,

$$ |A|\lesssim \Vert \Theta \Vert _{H^{|J|-1}}\Vert \Theta \Vert _{H^{|J|}} \lesssim e^{(1+c_{(0,|J|-1)})\Re \lambda \tau }e^{\Re \lambda \tau } \le e^{2(1+c_J)\Re \lambda \tau }, $$

and the term $\mathcal {H}_{J}^{\text {com}}$ is dealt with.

### Quadratic Term

In this section we prove the bound (7.30) for $\mathcal {H}_{J}^{\text {quad}}$; is a corollary of the following lemma:

#### Lemma 7.13

It holds that

$$ \Vert \mathcal {H}_J^{\text {quad}}\Vert _{L^2} \lesssim e^{2\Re \lambda \tau } $$

for all $$-k\le \tau \le \min _{p\le q}\tau _k^{(p)}$$.

We will prove this lemma in the next subsections. We split

$$ \mathcal {H}_J^{\text {quad}} =\mathcal {H}_J^{\text {quad},1} +\mathcal {H}_J^{\text {quad},2} +\mathcal {H}_J^{\text {quad},3}, $$

where

$$\begin{aligned} \mathcal {H}_J^{\text {quad},1}&=\frac{\partial ^J(\tilde{V}\cdot \nabla \Theta )}{R^{m-j_1}}\omega -\tilde{V}\cdot \nabla \left( \frac{\partial ^J\Theta }{R^{m-j_1}}\omega \right) ,\\ \mathcal {H}_J^{\text {quad},2}&=\frac{\partial ^J(\tilde{V}\cdot \nabla \Theta ^{\text {lin}})}{R^{m-j_1}}\omega -\tilde{V}\cdot \nabla \left( \frac{\partial ^J\Theta ^{\text {lin}}}{R^{m-j_1}}\omega \right) ,\\ \mathcal {H}_J^{\text {quad},3}&=\tilde{V}\cdot \nabla \left( \frac{\partial ^J\Theta ^{\text {lin}}}{R^{m-j_1}}\omega \right) . \end{aligned}$$

Recall that Θ corresponds to step *q*, and $\tilde{V}$ corresponds to step q-1, according to (7.23).

#### Quadratic Term 1

##### Lemma 7.14

It holds that

7.34 $$\begin{aligned} \begin{aligned} \mathcal {H}_J^{\text {quad},1} =&\frac{\omega }{R^{m-j_1}}\sum _{(0,0)\ne I\le J}\left( {\begin{array}{c}J\\ I\end{array}}\right) \left( \partial ^{I}\tilde{V}_R\partial ^{J-I}\partial _R\Theta +\partial ^{I}\left( \frac{\tilde{V}_\phi }{R}\right) \partial ^{J-I}\partial _\phi \Theta \right) \\&+\left( (m-j_1)+\frac{2R^2}{\omega }\right) \frac{\tilde{V}_R}{R}\frac{\partial ^J\Theta }{R^{m-j_1}}\omega . \end{aligned} \end{aligned}$$

##### Proof

Notice that since

$$ \tilde{V}\cdot \nabla \Theta =\tilde{V}_R\partial _R\Theta +\frac{\tilde{V}_\phi }{R}\partial _\phi \Theta , $$

by applying the Leibniz rule, we get that

$$ \partial ^J(\tilde{V}\cdot \nabla \Theta ) =\sum _{I\le J}\left( {\begin{array}{c}J\\ I\end{array}}\right) \left( \partial ^{I}\tilde{V}_R\partial ^{J-I}\partial _R\Theta +\partial ^{I}\left( \frac{\tilde{V}_\phi }{R}\right) \partial ^{J-I}\partial _\phi \Theta \right) . $$

We split this sum into the main term (I=0) and the reminder (I>0)

$$ \partial ^J(\tilde{V}\cdot \nabla \Theta ) =\tilde{V}\cdot \nabla \partial ^J\Theta +\sum _{(0,0)\ne I\le J}\left( {\begin{array}{c}J\\ I\end{array}}\right) \left( \partial ^{I}\tilde{V}_R\partial ^{J-I}\partial _R\Theta {+}\partial ^{I}\left( \frac{\tilde{V}_\phi }{R}\right) \partial ^{J-I}\partial _\phi \Theta \right) . $$

Multiplying both sides by ω and introducing the weight into the derivative of the main term (and absorbing the extra terms in the remainder), we get

$$ \frac{\omega }{R^{m-j_1}}(\tilde{V}\cdot \nabla \partial ^J\Theta ) =\tilde{V}\cdot \nabla \left( \frac{\partial ^J\Theta }{R^{m-j_1}}\omega \right) {+}\left( (m-j_1)+\frac{2R^2}{\omega }\right) \frac{\tilde{V}_R}{R}\frac{\partial ^J\Theta }{R^{m-j_1}}\omega , $$

as desired. □

##### Lemma 7.15

It holds that

$$ \Vert \mathcal {H}_J^{\text {quad},1}\Vert _{L^2} \lesssim e^{2\Re \lambda \tau }. $$

##### Proof

We want to bound the $L^2$ norm of the different terms appearing in (7.34). By Lemma 6.3 and (7.8) (which will be used repeatedly below), the last term in (7.34) can be bounded easily

$$ \left\| \frac{\tilde{V}_R}{R}\right\| _{L^\infty } \left\| \frac{\partial ^J\Theta }{R^{m-j_1}}\omega \right\| _{L^2} \lesssim \Vert \Theta \Vert _{Y^m}^2 \lesssim e^{2\Re \lambda \tau }. $$

Next, we proceed to bound the terms in (7.34) of the form

7.35 $$\begin{aligned} \frac{\omega }{R^{m-j_1}}\partial ^{I}\tilde{V}_R\partial ^{J-I}\partial _R\Theta , \end{aligned}$$

with $(0,0)\ne I\le J$. By applying the Leibniz rule on $\partial ^I(\tilde{V}\cdot e_R)$, we realize that, in order to bound (7.35), it is enough to control terms of the form

7.36 $$\begin{aligned} \frac{\omega }{R^{m-j_1}}\partial ^{\tilde{I}}\tilde{V}\partial ^{J-I}\partial _R\Theta , \end{aligned}$$

for any $(0,0)\ne \tilde{I}\le I$. If $|\tilde{I}|=0$, we distinguish two cases: $i_1=0$ and $i_1>0$. If $i_1=0$ then

$$ \left\| \frac{\tilde{V}}{R}\right\| _{L^\infty } \left\| \frac{\partial ^{J-I}\partial _R\Theta }{R^{m-(j_1+1)}}\omega \right\| _{L^2} \lesssim \Vert \Theta \Vert _{Y^m}^2 \lesssim e^{2\Re \lambda \tau }. $$

If $i_1>0$ then we use (7.16) in the first inequality and Lemma 7.5 in the second to obtain

$$ \Vert \tilde{V}\Vert _{L^\infty } \left\| \frac{\partial ^{J-I}\partial _R\Theta }{R^{m-j_1}}\omega \right\| _{L^2}\lesssim \Vert \Theta \Vert _{\bar{Y}^m}^2 \lesssim \Vert \Theta \Vert _{Y^m}^2 \lesssim e^{2\Re \lambda \tau }. $$

Here we have taken $r=j_1$ in the definition of $\bar{Y}_m$ since $|J|-|I|+1\le r+(j_2-i_2)\le m$.

For $|\tilde{I}|>0$, by rewriting polar into Cartesian coordinates (Lemma 7.3), we expand

$$\begin{aligned} (7.36)&=\frac{\omega }{R^{m-j_1}} \Bigg \lbrace \sum _{\begin{array}{c} 0<|K|\le |\tilde{I}| \\ \tilde{i}_1\le |K| \end{array}} q_K^{\tilde{I}} R^{|K|-\tilde{i}_1}\partial _X^K\tilde{V}\Bigg \rbrace \\&\quad \times \Bigg \lbrace \sum _{\begin{array}{c} 0<|L|\le |J-I|+1 \\ j_1-i_1+1\le |L| \end{array}} q_L^{J-I+(1,0)} R^{|L|-(j_1-i_1+1)}\partial _X^L\Theta \Bigg \rbrace \\&=\sum _{\begin{array}{c} 0<|K|\le |\tilde{I}| \\ \tilde{i}_1\le |K| \end{array}} \sum _{\begin{array}{c} 0<|L|\le |J-I|+1 \\ j_1-i_1+1\le |L| \end{array}} q_K^{\tilde{I}} q_L^{J-I+(1,0)} \partial _X^K\tilde{V} \frac{\partial _X^L\Theta }{R^{m-(i_1-\tilde{i}_1+|L+K|-1)}}\omega . \end{aligned}$$

If 0<|K|≤m-2, then by Lemma 7.5 and Sobolev embedding, we can bound the corresponding term by

$$ \Vert \partial _X^K\tilde{V}\Vert _{L^\infty } \left\| \frac{\partial _X^L\Theta }{R^{m-(i_1-\tilde{i}_1+|L+K|-1)}}\omega \right\| _{L^2}\lesssim \Vert \partial _X^K\tilde{V}\Vert _{H^2}\Vert \Theta \Vert _{\bar{Z}^m}\lesssim \Vert \Theta \Vert _{Y^m}^2 \lesssim e^{2\Re \lambda \tau }, $$

because $$|L|\le i_1-\tilde{i}_1+|L+K|-1\le |J|\le m$$.

If $m-1\le |K|\le m$, we instead bound the corresponding term by

$$ \Vert \partial _X^K\tilde{V}\Vert _{L^2} \left\| \frac{\partial _X^L\Theta }{R^{m-(i_1-\tilde{i}_1+|L+K|-1)}}\omega \right\| _{L^\infty }\lesssim e^{\Re \lambda \tau } \left\| \frac{\partial _X^L\Theta }{R^{m-(i_1-\tilde{i}_1+|L+K|-1)}}\omega \right\| _{L^\infty }. $$

We estimate the remaining term

$$\left\| \frac{\partial _X^L\Theta }{R^{m-(i_1-\tilde{i}_1+|L+K|-1)}}\omega \right\| _{L^\infty } $$

by considering separate cases. In the following arguments, we rely on the inequalities: $\tilde{I}\le I\le J$, $|K|\le |\tilde{I}|$, 0<|L|≤|J-I|+1 and |J|≤m. If |K|=m then necessarily $\tilde{I}=I=J$ and |L|=1, so that $m-(i_1-\tilde{i}_1+|L+K|-1)=0$. Hence, in order to control such term we have to estimate $D_X\Theta \,\omega$ in $L^\infty$. Suppose now that |K|=m-1, then we have that either |L|=1 or |L|=2. If |L|=2 then |I|=m-1 and thus $\tilde{I}=I$, so $m-(i_1-\tilde{i}_1+|L+K|-1)=0$ and therefore for this we have to estimate $D_X^2\Theta \,\omega$ in $L^\infty$. Finally, if |L|=1 then we deduce that $m-(i_1-\tilde{i}_1+|L+K|-1)$ is either 0 or 1, so we have to control $D_X\Theta \,\omega$ and $(D\Theta /R)\,\omega$ in $L^\infty$. In brief, we need to bound $D_X\Theta \,\omega$, $(D_X\Theta /R)\,\omega$ and $D_X^2\Theta \,\omega$ in $L^\infty$.

By applying the Sobolev embedding and Lemmas 7.4-7.5, we get

$$ \Vert D_X\Theta \,\omega \Vert _{L^\infty } \lesssim \Vert D_X\Theta \,\omega \Vert _{H^2} \lesssim \Vert \Theta \Vert _{Z^3}\simeq \Vert \Theta \Vert _{Y^3}\lesssim e^{\Re \lambda \tau } $$

and

$$ \Vert D_X^2\Theta \, \omega \Vert _{L^\infty } \lesssim \Vert D_X^2\Theta \,\omega \Vert _{H^2} \lesssim \Vert \Theta \Vert _{Z^4}\simeq \Vert \Theta \Vert _{Y^4}\lesssim e^{\Re \lambda \tau }. $$

Finally, by Lemma 6.3 and the Sobolev embedding,

$$\begin{aligned}  &   \left\| \frac{D_X\Theta }{R}\omega \right\| _{L^\infty } \lesssim \left\| \frac{D_X\Theta }{R}\right\| _{L^\infty (B)}+\left\| D_X\Theta \, \omega \right\| _{L^\infty (B^c)} \\  &   \quad \lesssim \Vert \Theta \Vert _{H^m}+\Vert D_X\Theta \,\omega \Vert _{H^2} \lesssim \Vert \Theta \Vert _{Y^m}\lesssim e^{\Re \lambda \tau }. \end{aligned}$$

We are left with the terms in (7.34) of the form

$$\begin{aligned} \frac{\omega }{R^{m-j_1}}\partial ^{I}\left( \frac{\tilde{V}_\phi }{R}\right) \partial ^{J-I}\partial _\phi \Theta , \end{aligned}$$

with $(0,0)\ne I\le J$. These terms can be treated similarly to (7.35) by applying the Leibniz rule to $\partial ^I((\tilde{V}\cdot e_\phi )/R)$ and then using analogous bounds. □

#### Quadratic Term 2

##### Lemma 7.16

It holds that

$$ \Vert \mathcal {H}_J^{\text {quad},2}\Vert _{L^2} \lesssim e^{2\Re \lambda \tau }. $$

##### Proof

Since the only difference between $\mathcal {H}_J^{\text {quad},2}$ and $\mathcal {H}_J^{\text {quad},1}$ is the replacement of Θ with $\Theta ^{\text{ l }in}$, the proof follows in an entirely analogous manner. □

#### Quadratic Term 3

##### Lemma 7.17

It holds that

$$ \Vert \mathcal {H}_J^{\text {quad},3}\Vert _{L^2} \lesssim e^{2\Re \lambda \tau }. $$

##### Proof

We have

$$ \tilde{V}\cdot \nabla \left( \frac{\partial ^J\Theta ^{\text {lin}}}{R^{m-j_1}}\omega \right) =\tilde{V}_R\partial _R\left( \frac{\partial ^J\Theta ^{\text {lin}}}{R^{m-j_1}}\omega \right) +\frac{\tilde{V}_\phi }{R}\partial _\phi \left( \frac{\partial ^J\Theta ^{\text {lin}}}{R^{m-j_1}}\omega \right) . $$

This can be directly bounded by the $Y^{m+1}$-norm of $\Theta ^{\text {lin}}$ and the $L^\infty$-norm of $\tilde{V}/R$.

□

## Appendix A.

### A.1. Preliminaries in Operator theory

In this section we prove Proposition 6.2. We start by recalling some classical definitions and results in Operator theory. In general, we will consider a linear operator $A:D(A)\subset H\rightarrow H$ acting on some Hilbert space *H*, where *D*(*A*) is the domain of *A*. For a fixed *H*, we will denote $\mathcal {L}$ and $\mathcal {K}$ by the space of bounded and compact operators on *H* respectively. The *spectrum* of *A* is defined as

$$ \sigma (A)=\{\lambda \in \mathbb {C}\,:\, (A-\lambda )\text { is not invertible}\}. $$

Let us suppose that *A* is a bounded operator. Then, *A* is called a *Fredholm Operator* if both the kernel $\text {Ker}(A)$ and the cokernel $H/\text {Im}(A)$ are finite dimensional. In this case, the *index* of *A* is the integer

$$ \text {Ind}(A) =\dim (\text {Ker}(A)) -\dim (H/\text {Im}(A)). $$

We recall a classical result in Spectral theory: the stability of the index of Fredholm operators with respect to compact perturbations (see e.g. [100, Theorem 5.26, Chapter IV]).

#### Proposition A.1

Let $A\in \mathcal {L}$ be a Fredholm operator and $K\in \mathcal {K}$. Then, $A+K\in \mathcal {L}$ is a Fredholm operator with $\text {Ind}(A+K)=\text {Ind}(A)$.

Next, we recall several classical results in Semigroup theory. The first one gives a characterization of strongly continuous semigroups (see e.g. [68, Corollary 3.6, Chapter II]).

#### Proposition A.2

Given $w\in \mathbb {R}$ and a linear operator *A*, the following are equivalent:

(i) *A* generates a strongly continuous semigroup satisfying $\Vert e^{\tau A}\Vert _{\mathcal {L}}\le e^{w\tau }$ for all $\tau \ge 0$.
(ii) *A* is closed, densely defined, and for any $\lambda \in \mathbb {C}$ with $\Re \lambda >w$, it holds that
  $$ \Vert (A-\lambda )^{-1}\Vert _{\mathcal {L}}\le \frac{1}{\Re \lambda - w}. $$

#### Definition A.1

A linear operator *A* generates a *contraction semigroup* $e^{\tau A}$ if it satisfies the conditions in Proposition A.2 for w=0.

Next, we recall that, given a strongly continuous semigroup $e^{\tau A}$ generated by an operator *A*, its *growth bound* is defined as

A.1 $$\begin{aligned}  &   \omega _0(A) =\inf \{w\in \mathbb {R}\,:\,\text {there exists }C_w\ge 1\text { such that }\nonumber \\  &   \Vert e^{\tau A}\Vert _{\mathcal {L}} \le C_w e^{w\tau }\text { for all }\tau \ge 0\}. \end{aligned}$$

The second result from Semigroup theory that we need (see e.g. [68, Corollary 2.11, Chapter IV]) relates (A.1) with the *essential bound*

$$ \omega _{\text {ess}}(A) =\inf _{\tau >0}\frac{1}{\tau }\log \Vert e^{\tau A}\Vert _{\mathcal {L}/\mathcal {K}}, $$

where $\mathcal {L}/\mathcal {K}$ is the quotient space (the so-called Calkin algebra), and the *spectral bound*

$$ s(A) =\sup \{\Re \lambda \,:\,\lambda \in \sigma (A)\}. $$

#### Proposition A.3

Given an operator *A* generating a strongly continuous semigroup, it holds that

$$ \omega _0(A) =\max \{\omega _{\text {ess}}(A),s(A)\}. $$

Moreover, $\sigma (A)\cap \{\Re \lambda \ge w\}$ is finite for any $w>\omega _{\text {ess}}(A)$.

The third result in Semigroup theory that we need is the stability of the essential bound with respect to compact perturbations (see e.g. [68, Proposition 2.12, Chapter IV]).

#### Proposition A.4

Given an operator *A* generating a strongly continuous semigroup, and $K\in \mathcal {K},$ it holds that

$$ \omega _{\text {ess}}(A+K) =\omega _{\text {ess}}(A). $$

#### A.1.1. Proof of Proposition 6.2

Before embarking on the proof, we present four auxiliary lemmas.

##### Lemma A.1

The resolvent map

A.2 $$\begin{aligned} \begin{aligned} (A_b-\lambda )^{-1}: [0,\infty )\times \mathbb {C}_+&\rightarrow \mathcal {L} \\ (b,\lambda )&\mapsto \left( \Theta \mapsto -\int _0^\infty e^{\tau (A_b-\lambda )}\Theta \,\textrm{d}\tau \right) \end{aligned} \end{aligned}$$

is well defined with

A.3 $$\begin{aligned} \Vert (A_b-\lambda )^{-1}\Vert _{\mathcal {L}}\le \frac{1}{\Re \lambda }. \end{aligned}$$

For any $\Theta \in H$, the map $(b,\lambda )\mapsto (A_b-\lambda )^{-1}\Theta$ is continuous.

##### Proof

The first statement follows directly from assumption (1) and Proposition A.2. In fact, a simple integration by parts shows that (A.2) is the inverse of $(A_b-\lambda )$. Alternatively, (A.2) is the Laplace transform of the semigroup $e^{\tau A_b}$.

For the second statement, let $\Theta \in H$. Given $(b_0,\lambda _0)\in [0,\infty )\times \mathbb {C}_+$, we want to show that the following limit holds in *H*

$$ (A_b-\lambda )^{-1}\Theta \rightarrow (A_{b_0}-\lambda _0)^{-1}\Theta , $$

as $(b,\lambda )\rightarrow (b_0,\lambda _0)$. We have

$$\begin{aligned} \Vert ((A_b-\lambda )^{-1}- (A_{b_0}-\lambda _0)^{-1})\Theta \Vert _H&\le \int _0^\infty \Vert (e^{\tau (A_b-\lambda )}-e^{\tau (A_{b_0}-\lambda _0)})\Theta \Vert _H\,\textrm{d}\tau \\&\le \int _0^\infty |e^{-\lambda \tau }-e^{-\lambda _0\tau }| \Vert e^{\tau A_b}\Theta \Vert _H\,\textrm{d}\tau =:I\\&+\int _0^\infty e^{-\lambda _0\tau } \Vert (e^{\tau A_b}-e^{\tau A_{b_0}})\Theta \Vert _H\,\textrm{d}\tau =:J, \end{aligned}$$

where we have added and subtracted $e^{\tau (A_b-\lambda _0)}\Theta$. By applying that the semigroup is contractive we show that

$$ I\le \left| \frac{1}{\lambda }-\frac{1}{\lambda _0}\right| \Vert \Theta \Vert _H \rightarrow 0 $$

as $\lambda \rightarrow \lambda _0$. For the second term we can apply the dominated convergence theorem and assumption (1) to deduce that J→0 as $b\rightarrow b_0$. □

##### Lemma A.2

For any $\lambda \in \mathbb {C}_+$, the operator $(L_b-\lambda )$ is Fredholm with index zero. Therefore, $\sigma (L_b)\cap \mathbb {C}_+$ consists of eigenvalues with finite multiplicity.

##### Proof

We split

A.4 $$\begin{aligned} L_b-\lambda =(A_b-\lambda )+C. \end{aligned}$$

Since $\Re \lambda >0$, the first term is invertible by Lemma A.1. In particular, it is a Fredholm operator with index zero. Since *C* is compact, the operator (A.4) is also Fredholm with index zero by Proposition A.1. Thus, the second claim follows. □

##### Lemma A.3

The map

A.5 $$\begin{aligned} \begin{aligned} Q: [0,\infty )\times \mathbb {C}_+&\rightarrow \mathcal {K} \\ (b,\lambda )&\mapsto (A_b-\lambda )^{-1}\circ C \end{aligned} \end{aligned}$$

is well defined and continuous.

##### Proof

By applying Lemma A.1 and that *C* is compact, we deduce that *Q* is well-defined. The continuity of *Q* in the operator norm follows from combining the fact that the resolvent map (A.2) is continuous for any fixed Θ, and that *C* can be approximated by finite rank operators. □

##### Lemma A.4

The essential bound satisfies

$$ \omega _{\text {ess}}(L_b)\le 0.$$

Therefore, $\sigma (L_b)\cap \{\Re \lambda >w\}$ is finite for any w>0.

##### Proof

By applying assumption (1), that *C* is compact, and Proposition A.4, we get that

$$ \omega _{\text {ess}}(A_b+C) =\omega _{\text {ess}}(A_b) \le 0. $$

The second part of the statement follows from Proposition A.3. □

With these preparations, we are ready to begin the proof Proposition 6.2. Since $L=L_0$, we know from Theorem 3.3 that there exists an eigenvalue $\lambda _0\in \sigma (L)\cap \mathbb {C}_+$ and an eigenfunction $0\ne W_0\in \text {Ker}(L-\lambda _0)$. We claim that there exists $0<b<b_0$ satisfying

A.6 $$\begin{aligned} \sigma (L_b)\cap \left\{ \Re \lambda >\frac{\Re \lambda _0}{2}\right\} \ne \emptyset . \end{aligned}$$

We will prove by contradiction that necessarily (A.6) is true for some $0<b\le b_0$. Without loss of generality we assume that $b_0\le \frac{\Re \lambda _0}{4}$.

Let us suppose that (A.6) is false. Thus, $(L_b-\lambda )$ is invertible for all $\lambda \in \mathbb {C}_+$ and $0<b\le b_0$. We will first prove that in fact $(L_b-\lambda )^{-1}$ is continuous as a function of (b,λ) restricted to a suitable compact domain.

By applying Lemma A.4 to b=0, we can take a ↺-oriented circle $\Gamma :\mathbb {T}\rightarrow \mathbb {C}_+\setminus \sigma (L)$ of radius strictly less than $b_0$ surrounding $\lambda _0$. By applying Lemma A.1 coupled with (A.5) and the decomposition

A.7 $$\begin{aligned} L_b-\lambda =(A_b-\lambda )\circ (I+Q(b,\lambda )), \end{aligned}$$

our hypothesis implies that I+Q maps continuously the compact set $[0,b_0]\times \Gamma (\mathbb {T})$ into

$$ \{A\in \mathcal {L}\,:\,A\text { invertible}\} =\bigcup _{N\in \mathbb {N}} \{A\in \mathcal {L}\,:\,\Vert A^{-1}\Vert _{\mathcal {L}}< N\}. $$

Since the last union forms an open cover, we deduce that there exists $N\in \mathbb {N}$ such that

$$ \Vert (I+Q(b,\lambda ))^{-1}\Vert _{\mathcal {L}}\le N, $$

uniformly in $(b,\lambda )\in [0,b_0]\times \Gamma (\mathbb {T})$, and thus, by applying (A.3) and (A.7), also

$$ \Vert (L_b-\lambda )^{-1}\Vert _{\mathcal {L}} \le \frac{N}{\Re \lambda }. $$

By applying these bounds and the resolvent identity

$$\begin{aligned}&(I+Q(b,\lambda ))^{-1}-(I+C(b',\lambda '))^{-1}\\&\quad =(I+Q(b,\lambda ))^{-1}\circ (Q(b,\lambda )-C(b',\lambda '))\circ (I+C(b',\lambda '))^{-1}, \end{aligned}$$

it follows that the map $(b,\lambda )\mapsto (I+Q(b,\lambda ))^{-1}$ is continuous from $[0,b_0]\times \Gamma (\mathbb {T})$ into $\mathcal {L}$. As a consequence, the same can be deduce for the map $(b,\lambda )\mapsto (L_b-\lambda )^{-1}$.

Once continuity is obtained, we can consider the Riesz projection

$$ P_{b,\Gamma }=-\frac{1}{2\pi i}\int _\Gamma (L_b-\lambda )^{-1}\,\textrm{d}\lambda . $$

and take limits under the integral sign by the dominated convergence theorem. Therefore, we have

$$ P_{b,\Gamma }(W_0) \rightarrow P_{0,\Gamma }(W_0), $$

as b→0. However, $P_{b,\Gamma }(W_0)=0$ for any $0<b\le b_0$ by hypothesis, while $P_{0,\Gamma }(W_0)=W_0\ne 0$, which is a contradiction.

Therefore, there exists $\lambda _b\in \sigma (L_b)\cap \{\Re \lambda >b_0\}$ for some $0<b\le b_0$. By Lemma A.2, there exists $0\ne W_b\in \textrm{Ker}(L_b-\lambda _b)$. This concludes the proof.

### A.2. Interchanging polar and Cartesian derivatives

#### Lemma A.5

(From Cartesian to Polar derivatives) For any multi-index $K=(k_1,k_2)$ with |K|>0, it holds that

A.8 $$\begin{aligned} \partial _X^Kf =\sum _{0<|J|\le |K|}p_J^K\frac{\partial _R^{j_1}\partial _\phi ^{j_2}f}{R^{|K|-j_1}} \end{aligned}$$

where $p_J^K=p_J^K(\cos \phi ,\sin \phi )$ is a |*K*|-homogeneous polynomial. Moreover, defining $p_{(0,0)}^{(0,0)}:=1$ and $p_J^K:=0$ if $j_1<0$, $j_2<0$ or |J|>|K| we obtain the following recursive formulae for $p_J^K$:

A.9 $$\begin{aligned} p_J^{(k_1+1,k_2)}&=-\sin \phi \,\partial _\phi p_J^K+\cos \phi \, p_{(j_1-1,j_2)}^K-\sin \phi \, p_{(j_1,j_2-1)}^K,\end{aligned}$$

A.10 $$\begin{aligned} p_J^{(k_1,k_2+1)}&=\cos \phi \,\partial _\phi p_J^K+\sin \phi \, p_{(j_1-1,j_2)}^K+\cos \phi \, p_{(j_1,j_2-1)}^K, \end{aligned}$$

for (0,0)≤J and |J|≤|K|+1.

#### Proof

We will prove (A.8) by induction on |*K*|. If |K|=1 we have the following

$$\begin{aligned} \partial _x f&=\cos \phi \,\partial _{R} f-\frac{1}{R}\sin \phi \,\partial _{\phi } f,\\ \partial _y f&=\sin \phi \,\partial _{R} f+\frac{1}{R}\cos \phi \,\partial _{\phi } f. \end{aligned}$$

Now, suppose that the formula holds for every |K|≤n. Then, given |K|=n,

$$\begin{aligned}&\partial _x^{k_1+1}\partial _y^{k_2}f=\partial _x\left( \partial _X^Kf\right) =\partial _x\left( \sum _{0<|J|\le |K|}p_J^K\frac{\partial _R^{j_1}\partial _\phi ^{j_2}f}{R^{|K|-j_1}}\right) \\&\quad =-\sum _{0<|J|\le |K|}\sin \phi \,\partial _\phi p_J^K\frac{\partial _R^{j_1}\partial _\phi ^{j_2}f}{R^{|K|+1-j_1}} \\&\quad +\sum _{1<|J|+1\le |K|+1}p_J^K\left( \cos \phi \,\frac{\partial _R^{j_1+1}\partial _\phi ^{j_2}f}{R^{|K|+1-(j_1+1)}} -\sin \phi \,\frac{\partial _R^{j_1}\partial _\phi ^{j_2+1}f}{R^{|K|+1-j_1}}\right) \end{aligned}$$

from where (A.9) is derived. In the other case,

$$\begin{aligned}&\partial _x^{k_1}\partial _y^{k_2+1}f=\partial _y\left( \partial _X^Kf\right) =\partial _x\left( \sum _{0<|J|\le |K|}p_J^K\frac{\partial _R^{j_1}\partial _\phi ^{j_2}f}{R^{|K|-j_1}}\right) \\&\quad =\sum _{0<|J|\le |K|}\cos \phi \,\partial _\phi p_J^K\frac{\partial _R^{j_1}\partial _\phi ^{j_2}f}{R^{|K|+1-j_1}} \\&\quad +\sum _{1<|J|+1\le |K|+1}p_J^K\left( \sin \phi \,\frac{\partial _R^{j_1+1}\partial _\phi ^{j_2}f}{R^{|K|+1-(j_1+1)}} +\cos \phi \,\frac{\partial _R^{j_1}\partial _\phi ^{j_2+1}f}{R^{|K|+1-j_1}}\right) , \end{aligned}$$

from hence (A.10) is derived. □

#### Lemma A.6

(From Polar to Cartesian derivatives) For any multi-index $J=(j_1,j_2)$ with |J|>0, it holds that

A.11 $$\begin{aligned} \partial _R^{j_1}\partial _\phi ^{j_2}f =\sum _{\begin{array}{c} 0<|K|\le |J| \\ j_1\le |K| \end{array}} q_K^J R^{|K|-j_1}\partial _X^K f, \end{aligned}$$

where $q_K^J=q_K^J(\cos \phi ,\sin \phi )$ is a |*K*|-homogeneous polynomial. Moreover, defining $q_{(0,0)}^{(0,0)}:=1$ and $q_K^J:=0$ if $k_1<0$, $k_2<0$ or |K|>|J| we obtain the following recursive formulae for $q_K^J$:

A.12 $$\begin{aligned} q_K^{(j_1+1,j_2)}&=(|K|-j_1)q_K^J+\cos \phi \, q_{(k_1-1,k_2)}^J+\sin \phi \, q_{(k_1,k_2-1)}^J, \end{aligned}$$

A.13 $$\begin{aligned} q_K^{(j_1,j_2+1)}&=\partial _\phi q_K^J-\sin \phi \, q_{k_1-1,k_2}^J+\cos \phi \, q_{k_1,k_2-1}^J, \end{aligned}$$

for (0,0)≤K and |K|≤|J|+1.

#### Proof

We will prove (A.11) by induction on |*J*|. If |J|=1 we have the following formulae:

$$\begin{aligned} \partial _R f&=\cos \phi \,\partial _{x} f+\sin \phi \,\partial _{y} f=\frac{x}{R}\cdot \nabla f,\\ \partial _\phi f&=-R\sin \phi \, \partial _{x} f+R\cos \phi \, \partial _{y} f=R\frac{x^\perp }{R} \cdot \nabla f. \end{aligned}$$

Now, suppose that the formula holds for every |J|≤n. Then, given |J|=n,

$$\begin{aligned} \partial _R^{j_1+1}\partial _\phi ^{j_2}f=&\,\partial _R\left( \partial _R^{j_1}\partial _\phi ^{j_2}f\right) =\partial _R\left( \sum _{\begin{array}{c} 0<|K|\le |J| \\ j_1\le |K| \end{array}} q_K^J R^{|K|-j_1}\partial _X^K f\right) \\ =&\,\sum _{\begin{array}{c} 0<|K|\le |J| \\ j_1+1\le |K| \end{array}} q_K^J (|K|-j_1) R^{|K|-(j_1+1)}\partial _X^K f\\&\quad + \sum _{\begin{array}{c} 1<|K|+1\le |J|+1 \\ j_1+1\le |K|+1 \end{array}}q_K^J R^{|K|+1-(j_1+1)}\\&\quad \times (\cos \phi \,\partial _x^{k_1+1}\partial _y^{k_2} f+\sin \phi \,\partial _x^{k_1}\partial _y^{k_2+1}f), \end{aligned}$$

from which one deduces (A.12). In the other case,

$$\begin{aligned} \partial _R^{j_1}\partial _\phi ^{j_2+1}f=&\,\partial _\phi \left( \partial _R^{j_1}\partial _\phi ^{j_2}f\right) =\partial _\phi \left( \sum _{\begin{array}{c} 0<|K|\le |J| \\ j_1\le |K| \end{array}} q_K^J R^{|K|-j_1}\partial _X^K f\right) \\ =&\,\sum _{\begin{array}{c} 0<|K|\le |J| \\ j_1\le |K| \end{array}} \partial _\phi q_K^J R^{|K|-j_1}\partial _X^K f \\&\quad + \sum _{\begin{array}{c} 1<|K|+1\le |J|+1 \\ j_1+1\le |K|+1 \end{array}}q_K^J R^{|K|+1-j_1}(-\sin \phi \,\partial _x^{k_1+1}\partial _y^{k_2} f{+}\cos \phi \,\partial _x^{k_1}\partial _y^{k_2+1}f), \end{aligned}$$

from which we deduce (A.13). □
